# Cobalt-catalyzed regioselective stereoconvergent Markovnikov 1,2-hydrosilylation of conjugated dienes†
†Electronic supplementary information (ESI) available. See DOI: 10.1039/c7sc04002d


**DOI:** 10.1039/c7sc04002d

**Published:** 2017-11-27

**Authors:** Hui Leng Sang, Songjie Yu, Shaozhong Ge

**Affiliations:** a Department of Chemistry , National University of Singapore , 3 Science Drive 3 , Singapore 117543 , Singapore . Email: chmgsh@nus.edu.sg

## Abstract

We report the first stereoconvergent Markovnikov 1,2-hydrosilylation of conjugated dienes using catalysts generated from bench-stable Co(acac)_2_ and phosphine ligands. A wide range of *E*/*Z*-dienes underwent this Markovnikov 1,2-hydrosilylation in a stereoconvergent manner, affording (*E*)-allylsilanes in high isolated yields with high stereoselectivities (*E*/*Z* = >99 : 1) and high regioselectivities (*b*/*l* up to > 99 : 1). Mechanistic studies revealed that this stereoconvergence stems from a σ–π–σ isomerization of an allylcobalt species generated by the 1,4-hydrometalation of *Z*-dienes. In addition, a cobalt catalyst that can only catalyze the hydrosilylation of the *E*-isomer of an (*E*/*Z*)-diene was identified, which allows the separation of the (*Z*)-isomer from an isomeric mixture of (*E*/*Z*)-dienes. Furthermore, asymmetric hydrosilylation of (*E*)-1-aryl-1,3-dienes was studied with Co(acac)_2_/(*R*)-difluorphos and good enantioselectivities (er up to 90 : 10) were obtained.

## Abstract 2

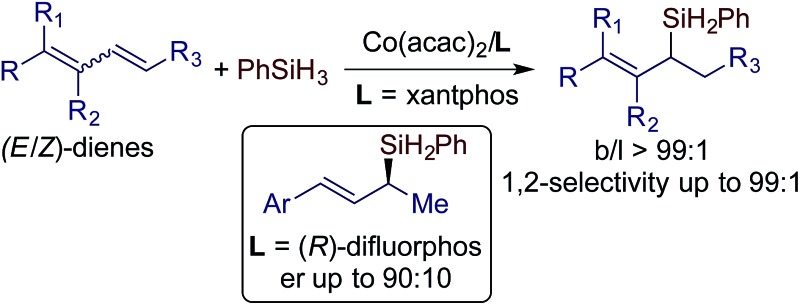
The first transition metal-catalyzed stereoconvergent Markovnikov 1,2-hydrosilylation of (*E*/*Z*)-dienes was effectively achieved with excellent *E*-selectivities using a cobalt catalyst.

## Introduction

Allylsilanes are synthetically valuable building blocks due to their non-toxicity, high stability and versatile applications in organic synthesis and material science.^1^ Among the methods for allylsilane synthesis,^2^ transition metal-catalyzed hydrosilylation of conjugated dienes is the most straightforward approach to prepare these synthetically valuable allylsilanes.^3^ Recently, the hydrosilylation of alkenes^4^ and alkynes^5^ has been extensively studied with cobalt catalysts,^6^ particularly due to their higher abundance and lower toxicity compared to platinum catalysts for hydrosilylation reactions.^7^ More importantly, recent studies indicate that cobalt catalysts can offer a precise control of regio- and stereoselectivities.^5^ However, highly selective cobalt catalysts for the hydrosilylation of conjugated dienes still remain rare and are under development.3c,^8^

**Scheme 1:**
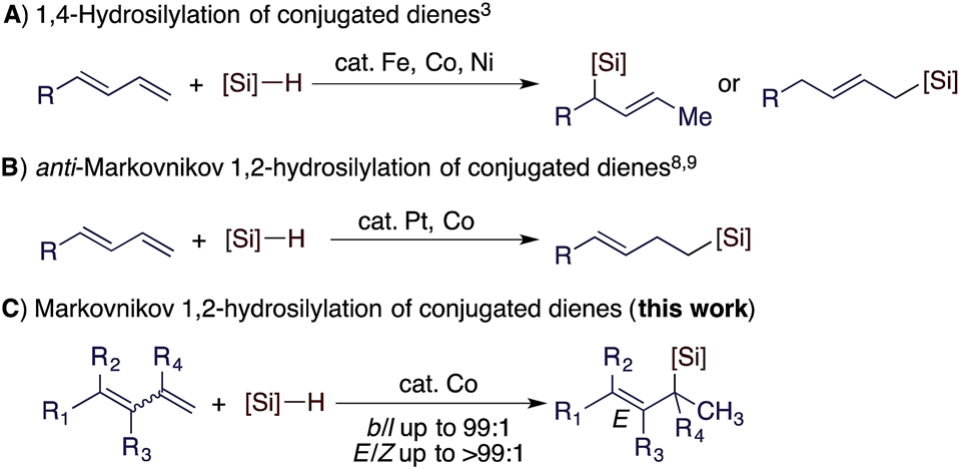
Transition metal-catalyzed hydrosilylation of conjugated dienes.

Conjugated dienes can undergo 1,4- and 1,2-hydrosilylation and the selectivity is dependent on the catalyst employed. The majority of the catalysts based on Fe, Co, and Ni show high selectivity towards 1,4-hydrosilylation (Scheme 1A).^3^ For example, Hilt reported a highly selective cobalt catalyst for the 1,4-hydrosilylation of isoprene in the presence of P(*n*-Bu)_3_,3*c* and Ritter reported a well-defined Fe(0) complex ligated by 2-iminopyridines for the 1,4-hydrosilylation of 1,3-dienes.3*d* In contrast, the 1,2-hydrosilylation of conjugated dienes has been barely studied, and two catalysts based on Pt and Co have been used to catalyze this 1,2-hydrosilylation.^8^,^9^ In addition, these two catalysts show high selectivity towards anti-Markovnikov hydrosilylation (Scheme 1B). However, transition metal catalysts for Markovnikov 1,2-hydrosilylation of conjugated dienes, especially for stereoconvergent Markovnikov 1,2-hydrosilylation of *E*/*Z*-dienes, still remain unknown. Driven by our research interest in developing base metal catalysts for transformations of unsaturated organic molecules,4f,5f,^10^ herein we report the highly selective cobalt-catalyzed stereoconvergent Markovnikov 1,2-hydrosilylation of a wide range of functionalized conjugated (*E*/*Z*)-dienes (Scheme 1C). In addition, we also identified a cobalt catalyst that selectively catalyzes the hydrosilylation of the (*E*)-isomer of an (*E*/*Z*)-diene with the (*Z*)-isomer unreacted. This discovery would represent a convenient protocol to purify (*Z*)-dienes from (*E*/*Z*)-isomeric dienes, which are generally more accessible than stereodefined (*Z*)- or (*E*)-dienes.

## Results and discussion

We chose the reaction of (*E*)-1-phenyl-1,3-butadiene with PhSiH_3_ to evaluate the reaction conditions for this Co-catalyzed hydrosilylation of conjugated dienes. We tested this reaction with cobalt catalysts generated from Co(acac)_2_ and various nitrogen- and phosphine-based ligands. The selected examples are summarized in Table 1. In general, these reactions were conducted with 3 mol% of Co(acac)_2_ and 3 mol% ligand at 50 °C for 3 h.

**Table 1:** Evaluation of the conditions for the Co-catalyzed hydrosilylation of 1-phenyl-1,3-butadiene^*a*^

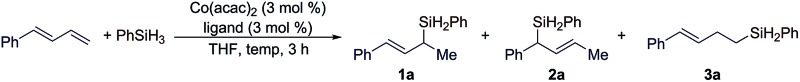					
Entry	Ligand	Temperature	Conversion^*b*^ (%)	Yield of **1a**^*b*^ (%)	**1a**/**2a**/**3a**^*b*^
1	^Ph^PDI	50 °C	>99	27	31 : — : 69
2	^TF^APDI	50 °C	>99	33	35 : — : 65
3	PyBox	50 °C	>99	49	56 : 4 : 40
4	dppm	50 °C	28	23	>99 : — : —
5	dppe	50 °C	78	77	>99 : — : —
6	dppbz	50 °C	9	5	>99 : — : —
7	binap	50 °C	93	76	>99 : — : —
8	xantphos	50 °C	>99	81	>99 : — : —
9^*c*^	xantphos	rt	>99	87	>99 : — : —
10^*c*^ ^,^^*d*^	xantphos	rt	>99	83	96 : 4 : —
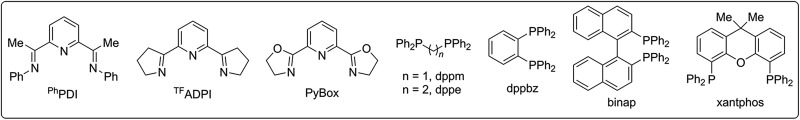					

^*a*^Conditions: (*E*)-diene (0.400 mmol), PhSiH_3_ (0.500 mmol), Co(acac)_2_ (12.0 μmol), ligand (12.0 μmol), and THF (1 mL) for 3 h.

^*b*^The conversion of diene, yield of **1a**, and the ratio of products **1a**, **2a**, and **3a** were determined by GC analysis with tridecane as the internal standard.

^*c*^Catalyst (1 mol%).

^*d*^A mixture of (*E*/*Z*)-1-phenyl-1,3-butadiene (*E*/*Z* = 45 : 55) was used.

Cobalt catalysts generated from the combination of Co(acac)_2_ and nitrogen-based ligands, such as ^Ph^PDI, ^TF^ADPI or PyBox, did catalyze the 1,2-hydrosilylation of (*E*)-1-phenyl-1,3-butadiene to full conversions, but these reactions produced a mixture of products **1a** and **3a** with low selectivities (entries 1–3 in Table 1).^8^ The reactions conducted with a combination of Co(acac)_2_ and bisphosphine ligands, such as dppm, dppe, or dppbz, proceeded to low to modest conversions, but with complete selectivities (>99%) for Markovnikov 1,2-hydrosilylation (entries 4–6 in Table 1). In particular, the reactions catalyzed by the combination of Co(acac)_2_ and binap or xantphos proceeded to high or full conversions with excellent selectivities to branched allylsilane **1a** (entries 7 and 8 in Table 1). As dienes are thermally less stable and can undergo polymerization, we tested this hydrosilylation at lower temperatures. The reaction conducted with 1 mol% of Co(acac)_2_ and 1 mol% of xantphos at room temperature proceeded to full conversion and afforded the desired allylsilane **1a** in an increased yield with an excellent Markovnikov selectivity (entry 9 in Table 1).

As (*E*/*Z*)-isomeric mixtures of dienes are synthetically more accessible than stereodefined (*E*)- or (*Z*)-dienes, we tested this hydrosilylation with a mixture of (*E*/*Z*)-isomeric 1-phenyl-1,3-butadiene to check the stereoconvergency for this reaction. To our delight, both (*Z*)- and (*E*)-1-phenyl-1,3-dienes underwent this cobalt-catalyzed Markovnikov 1,2-hydrosilylation and produced branched allylsilane **1a** in high yield with high selectivity (entry 10 in Table 1). Different from the hydrosilylation of (*E*)-1-phenyl-1,3-diene (entry 9 in Table 1), the reaction with a mixture of (*E*/*Z*)-1-phenyl-1,3-butadienes also generated a detectable amount (4%) of branched allylsilane **2a**, a product resulting from the 1,4-hydrosilylation of a diene.

Under the identified conditions (entry 9 in Table 1), we studied the scope of conjugated *trans*-dienes for this reaction. These results are summarized in Table 2. In general, a wide range of conjugated *trans*-dienes reacted smoothly with PhSiH_3_ in the presence of 1 mol% of Co(acac)_2_ and xantphos at room temperature, affording the corresponding (*E*)-allylsilanes (**1a–1n** in Table 2) in high yields (64–92%) with excellent regioselectivities (*b*/*l* = >99 : 1). The scope of these *trans*-dienes encompassed aryl-substituted (**1a–1h** in Table 2), alkyl-substituted (**1i–1k** in Table 2), and multiple-substituted dienes (**1a–1h** in Table 2). The GC-MS analysis of the crude mixtures of these reactions revealed that organosilane products from either 1,4-hydrosilylation or anti-Markovnikov 1,2-hydrosilylation of these *trans*-dienes were not formed during the Co-catalyzed Markovnikov 1,2-hydrosilylation. In addition, we also tested this hydrosilylation with secondary hydrosilanes (Ph_2_SiH_2_ and PhMeSiH_2_), and these reactions proceeded smoothly to afford tertiary cinnamylsilanes (**1a′** and **1a′′** in Table 2) in high isolated yields. However, this Co-catalyzed hydrosilylation did not occur with dialkylsilane (Et_2_SiH_2_) or tertiary hydrosilanes, such as (EtO)_3_SiH and (EtO)_2_MeSiH.

**Table 2:** Scope of *trans*-dienes for the Co-catalyzed Markovnikov 1,2-hydrosilylation^*a*^

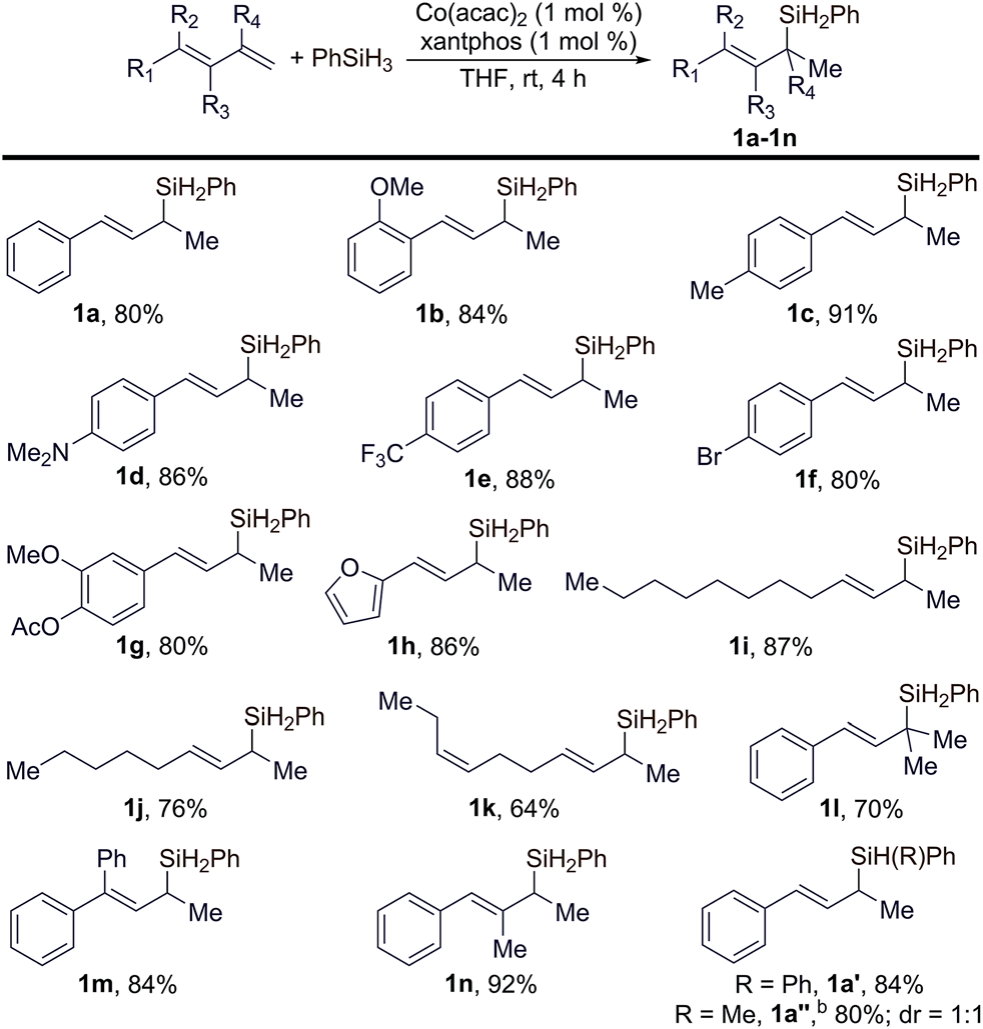

^*a*^Conditions: (*E*)-diene (0.400 mmol), PhSiH_3_ (0.500 mmol), Co(acac)_2_ (4.0 μmol), ligand (4.0 μmol), and THF (1 mL) for 3 h.

^*b*^3 mol% catalyst.

Subsequently, we studied the scope of conjugated dienes containing a mixture of (*E*/*Z*)-isomeric 1,3-dienes for this hydrosilylation reaction, and the results are summarized in Table 3. Generally, a wide range of (*E*/*Z*)-dienes, with *E*/*Z* ratios given in the brackets in Table 3, underwent this Markovnikov 1,2-hydrosilylation in a stereoconvergent manner with full conversions, affording the corresponding (*E*)-allylsilanes (**1a–1z** in Table 3) in high isolated yields with high regio- and stereoselectivities (*b*/*l* = >99 : 1; *E*/*Z* = >99 : 1). The GC-MS analysis of the crude reaction mixtures indicated that these reactions also produced small amounts of 1,4-hydrosilylation products, and the ratios of the products from 1,2- and 1,4-hydrosilylation are listed in Table 3 and abbreviated as 1,2/1,4 ratios.

**Table 3:** Scope of (*E*/*Z*)-dienes for stereoconvergent hydrosilylation reactions^*a*^

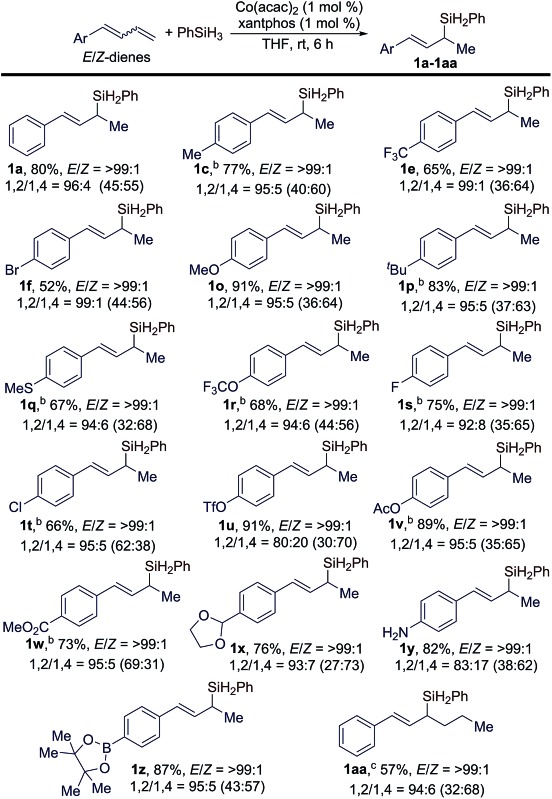

^*a*^Conditions: (*E*/*Z*)-diene (0.400 mmol), PhSiH_3_ (0.500 mmol), Co(acac)_2_ (4.0 μmol), xantphos (4.0 μmol), and THF (1 mL) at rt for 6 h; yield of isolated product; 1,2/1,4 ratios refer to 1,2-/1,4-hydrosilylation, the ratios in brackets are the *E*/*Z* ratios of the conjugated butadiene reagents.

^*b*^Reactions were conducted at 5 °C for 24 h.

^*c*^3 mol% catalyst at rt for 48 h.

The data in Table 3 indicate that the electronic properties of the aryl substituents do not have a significant influence on the stereoconvergence and regioselectivity of these hydrosilylation reactions (*e.g.***1a**, **1e**, and **1o** in Table 3). This Co-catalyzed transformation can tolerate various reactive groups, such as trifluoromethyl ether (**1r**), halogens (**1f**, **1s**, and **1t**), triflate (**1u**), ester (**1v** and **1w**), acetal (**1x**), unprotected primary aniline (**1y**), and pinacol boronic ester (**1z**). In addition, ((1*E*/*Z*,3*E*)-hexa-1,3-dien-1-yl)benzene, an internal 1,3-diene, also underwent this stereoconvergent hydrosilylation to afford allylsilane **1aa** in high isolated yield with high stereoselectivity (*E*/*Z* = >99 : 1).

As both the Co(acac)_2_ and xantphos used for this hydrosilylation reaction are bench-stable, we tested the hydrosilylation of 1-(buta-1,3-dien-1-yl)-4-methoxybenzene with PhSiH_3_ on a 10 mmol scale with 1 mol% of Co(acac)_2_/xantphos weighed on the benchtop without using a dry box. This reaction proceeded to the full conversion of the diene substrate and afforded **1o** in 87% isolated yield (eqn (1)).1
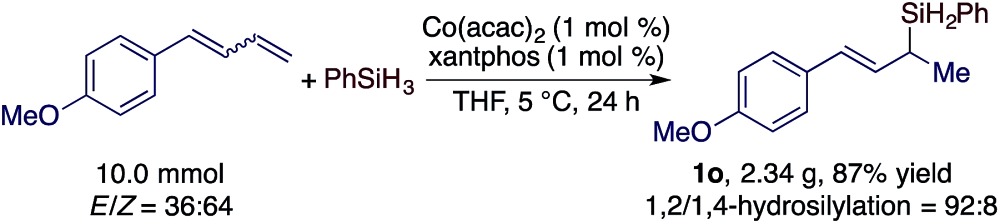



**Scheme 2:**
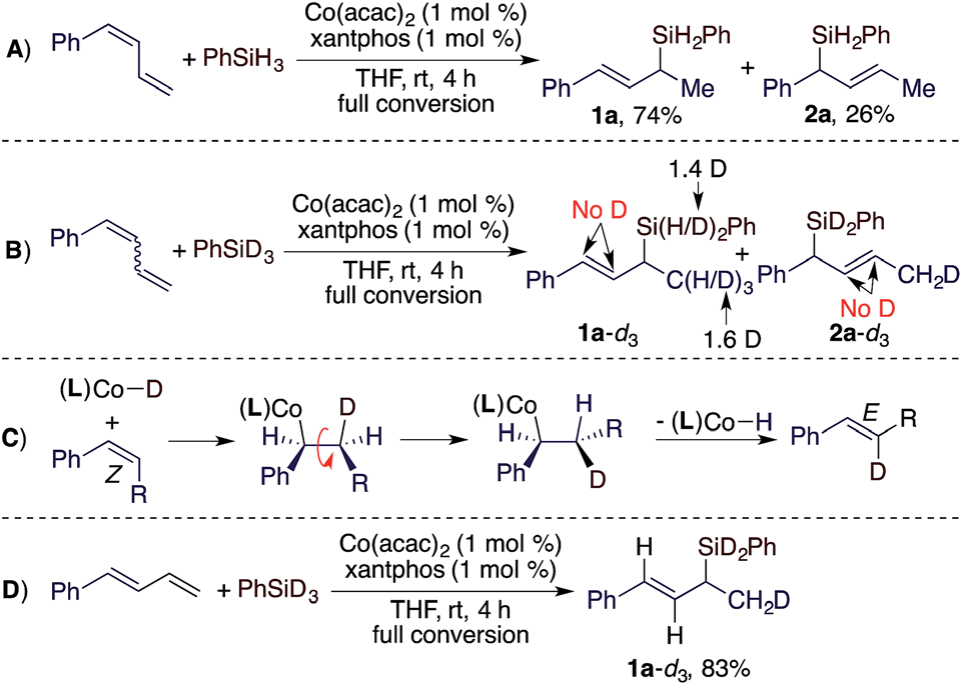
Hydrosilylation of stereodefined diene using Co(acac)_2_/xantphos.

To understand the stereoconvergence of this hydrosilylation of (*E*/*Z*)-dienes, we analyzed the reaction of (*E*/*Z*)-1-phenyl-1,3-butadiene and found that the (*E*)-isomer was consumed at a significantly higher rate than the (*Z*)-isomer.^11^ In addition, the hydrosilylation of (*Z*)-1-phenyl-1,3-butadiene was studied, and this reaction afforded the 1,2-hydrosilylation product **1a** together with a significant amount of the 1,4-hydrosilylation product **2a** (**1a**:**2a** = 74 : 26, Scheme 2A). The results of this reaction and the reactions of the *E*-isomer (entry 9 in Table 1) and the mixture of the (*E*/*Z*)-isomers (entry 10 in Table 1) suggest that allylsilane **2a** was formed by 1,4-hydrosilylation of the (*Z*)-isomer. To provide insight into the isomerization of the internal *Z*-alkene in the diene to the *E*-alkene in product **1a**, we subsequently conducted a deuterium-labelling experiment using the (*E*/*Z*)-isomers and PhSiD_3_ (Scheme 2B) and found that deuterium was solely incorporated into the methyl groups of **1a** and **2a**. This lack of deuterium incorporation onto the internal vinylic carbons suggests that this *E*/*Z*-isomerization through migratory insertion of the *Z*-alkene into a Co–H species followed by β-H elimination,^12^ as indicated in Scheme 2C, is unlikely. Furthermore, we also tested the reaction of the (*E*)-isomer with PhSiD_3_ and the same deuterium incorporation was observed (Scheme 2D).

**Scheme 3:**
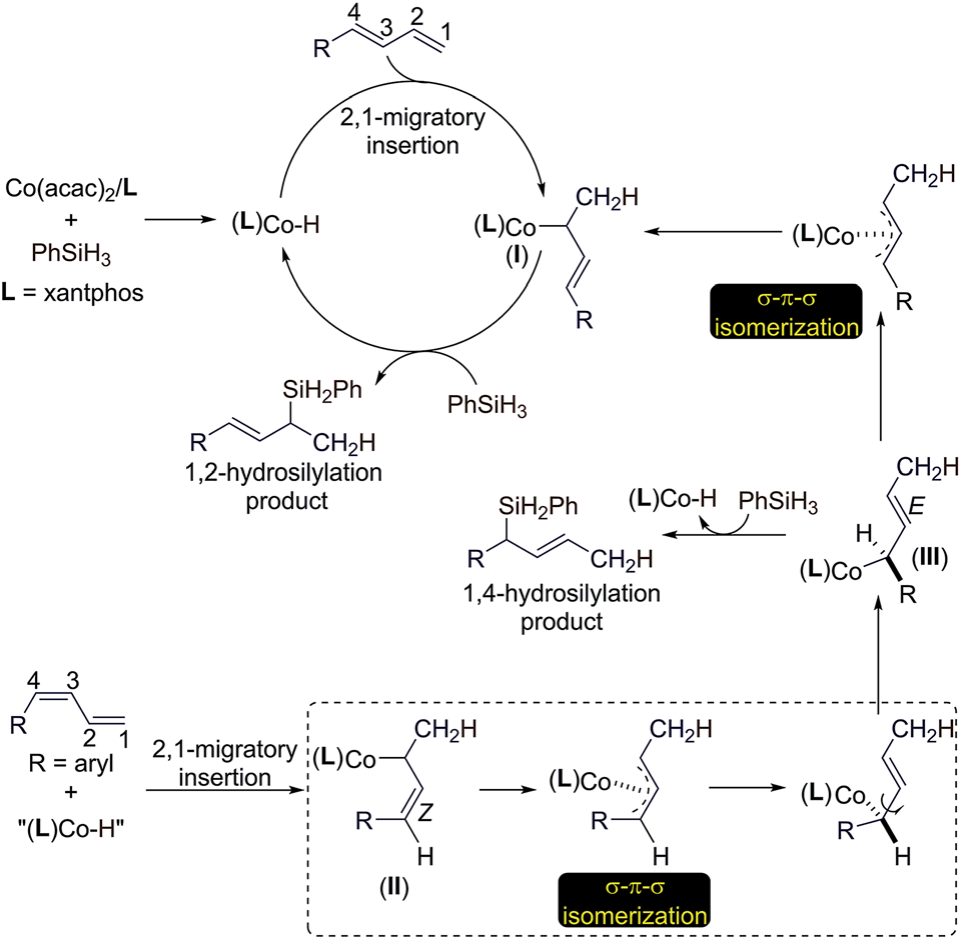
Proposed catalytic pathways for the Co-catalyzed stereoconvergent Markovnikov hydrosilylation of dienes.

Based on the results of the experiments in Scheme 2 and the precedent of Co-catalyzed hydrosilylation of alkenes,4f,^6^ we proposed a hydrometalation pathway with a Co(i)–H intermediate for this Co-catalyzed Markovnikov hydrosilylation of conjugated dienes (Scheme 3). 2,1-Migratory insertion of the (*E*)-diene into a Co–H species forms an allylcobalt intermediate **I**, which turns over with PhSiH_3_ to release the allylsilane product and regenerate the Co–H species.

For the hydrosilylation of (*Z*)-dienes, 2,1-migratory insertion of (*Z*)-dienes occurs to generate an allylcobalt species **II**, which undergoes σ–π–σ isomerization to form the allylcobalt intermediate **III**.^13^ This allylcobalt species reacts with PhSiH_3_ to give a 1,4-hydrosilylation product. In addition, the allylcobalt species **III** also undergoes σ–π–σ isomerization to generate the allylcobalt intermediate **I**, and this explains the observed stereoconvergency of the (*Z*/*E*)-diene hydrosilylation. Both allylcobalt species **I** and **III** can react with PhSiH_3_ to generate allylsilane products and the difference in the sterics around the Co–C bonds in these two allylcobalt species may account for the product ratio observed for the reaction listed in Scheme 2A.

**Scheme 4:**
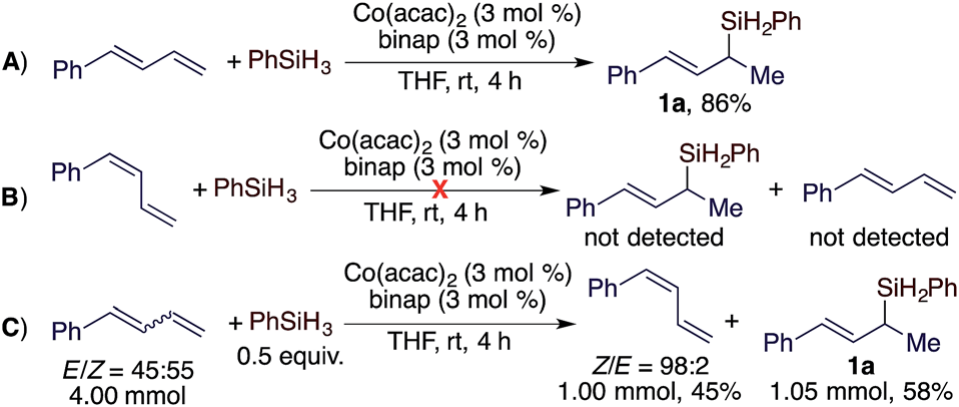
Hydrosilylation of an *E*/*Z*-mixture of dienes using Co(acac)_2_/binap.

After developing this stereoconvergent hydrosilylation reaction, we rationalized that the separation of a (*Z*)-diene from *Z*/*E*-diene mixtures could be achieved if we could identify a cobalt catalyst that can convert only the (*E*)-isomer of dienes. We tested various cobalt catalysts generated from the combination of Co(acac)_2_ and bisphosphine ligands for this purpose. To our delight, we found that the cobalt complex from Co(acac)_2_/binap was active for Markovnikov 1,2-hydrosilylation of (*E*)-1-phenyl-1,3-diene (Scheme 4A) but did not catalyze the hydrosilylation or the isomerization of (*Z*)-1-phenyl-1,3-diene (Scheme 4B). Then, we conducted this hydrosilylation reaction with a *E*/*Z*-mixture of 1-phenyl-1,3-diene (Scheme 4C). As expected, this reaction afforded (*E*)-allylsilane **1a** in 58% isolated yield and (*Z*)-1-phenyl-1,3-diene was recovered in 45% isolated yield with a *Z*/*E* ratio of 98 : 2.

We subsequently studied cobalt catalysts generated from Co(acac)_2_ and chiral bisphosphine ligands in order to develop the asymmetric Markovnikov 1,2-hydrosilylation of conjugated dienes.^14^ After evaluating various chiral phosphine ligands (see ESI† for details), we found that the hydrosilylation of *trans*-diene with PhSiH_3_ conducted with Co(acac)_2_ and (*R*)-difluorphos proceeded smoothly to afford the corresponding allylsilanes in good yield and good er (eqn (2)).2
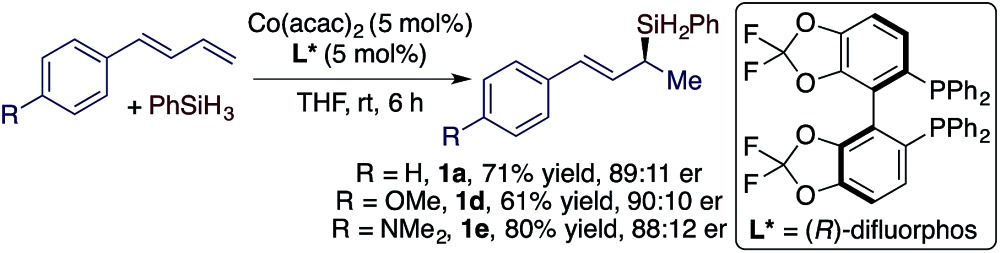



## Conclusions

In summary, we have developed the first Co-catalyzed Markovnikov 1,2-hydrosilylation of conjugated dienes with a catalyst generated from Co(acac)_2_ and xantphos. A broad scope of *trans*-dienes underwent this Markovnikov hydrosilylation to afford (*E*)-allylsilanes in high isolated yields and with excellent regioselectivities (*b*/*l* = >99 : 1). In addition, (*E*/*Z*)-isomeric 1,3-dienes reacted in a stereoconvergent manner to form (*E*)-allylsilanes with good to excellent regioselectivity (ratios of 1,2/1,4-hydrosilylation up to 99 : 1). This stereoconvergence resulted from a σ–π–σ isomerization of the allylcobalt intermediate. In particular, we also identified a cobalt catalyst, Co(acac)_2_/binap, for selectively converting the (*E*)-isomer of a mixture of (*E*/*Z*)-isomers, and this allows the separation of (*Z*)-dienes from a mixture of (*E*/*Z*)-dienes.

## Experimental details

### General procedures for stereoconvergent hydrosilylation of (*E*/*Z*)-dienes

In an Ar-filled glovebox, a mixture of Co(acac)_2_ (4.0 μmol) and xantphos (4.0 μmol) in THF (1 mL) was added into a 4 mL screw-capped vial containing a magnetic stirring bar. The resulting mixture was stirred for 2 min prior to adding phenylsilane (0.500 mmol) and (*E*/*Z*)-1,3-dienes (0.400 mmol) successively. The vial was removed from the glove box, and the mixture was stirred at room temperature for 6 hours. After that, the crude reaction mixture was concentrated under vacuum and the residue was purified by flash column chromatography using a mixture of ethyl acetate and hexane as an eluent. The conditions for the flash chromatography and the data for the characterization of the products are listed in the ESI.†

## Conflicts of interest

The authors declare no conflict of interest.

## Supplementary Material

Supplementary information
