# Supplementary material for: Cobalt-catalyzed regioselective stereoconvergent Markovnikov 1,2-hydrosilylation of conjugated dienes
Source: Chem Sci. 2017 Nov 27;9(4):973–8. doi: 10.1039/c7sc04002d (PMC5873224; doi:10.1039/c7sc04002d)
Supplement: Supplementary file 1 [file SC-009-C7SC04002D-s001.pdf]

Electronic Supplementary Material (ESI) for Chemical Science.
This journal is © The Royal Society of Chemistry 2017

*Supporting Information for*

# Cobalt-Catalyzed Regioselective Stereoconvergent Markovnikov 1,2-Hydrosilylation of Conjugated Dienes

**Hui Leng Sang, Songjie Yu and Shaozhong Ge***

Department of Chemistry, National University of Singapore, 3 Science Drive 3, Singapore 117543, Singapore

Email: chmgsh@nus.edu.sg

**Table of Contents**

**General Remarks** S2

**Reaction Screening of Silanes for Hydrosilylation of (*E*)-1-phenyl-1,3-butadiene** S3

**Reaction Screening of Ligands for Asymmetric Hydrosilylation of (*E*)-1-phenyl-1,3-butadiene** S4

**General procedure for Co-catalyzed Markovnikov 1,2-hydrosilylation of *trans*-1,3-dienes & Characterization Data of Isolated Products** S5

**General procedure for Co-catalyzed Markovnikov 1,2-hydrosilylation of *trans/cis*-1,3-dienes mixture & Characterization Data of Isolated Products** S12

**General procedure for Separation of *cis*-1,3-dienes via hydrosilylation reaction & Characterization Data of Isolated Products** S19

**General procedure for Co-catalyzed Markovnikov 1,2-hydrosilylation of trans/cis 1,3-dienes (Outside glovebox under nitrogen protection)** S20

**General procedure for gram-scale Co-catalyzed Markovnikov 1,2-hydrosilylation of *trans/cis*-1,3-dienes mixture** S20

**General procedure for asymmetric Co-catalyzed Markovnikov 1,2-hydrosilylation of *trans*-1,3-dienes & Characterization Data of Isolated Products** S21

**Mechanistic studies:** S27

**Procedure for Deuterium-labeling Experiments** S27

**Reaction Monitoring** S26

**Reference** S26

**NMR Spectra ($^1H$, $\{^1H\}^{13}C$ and $^{19}F$)** S27

## General Remarks

All the manipulations were performed in an argon-filled glovebox, unless mentioned otherwise. THF, toluene, and hexane were purified by passing the degassed solvents ($N_2$) through a column of activated alumina (solvent purification system purchased from Innovative Technologies, Newburyport, MA). The following chemicals were purchased and used as received: $Co(acac)_2$ (99%, Sigma-Aldrich), $CoCl_2$ (99.7%, Sigma-Aldrich), $CoBr_2$ (99%, Sigma-Aldrich), $Co(OAc)_2$ (99.99%, Sigma-Aldrich), $PhSiH_3$ (97%, Sigma-Aldrich), $Ph_2SiH_2$ (97%, Sigma-Aldrich), $MePhSiH_2$ (98%, Sigma-Aldrich), $Me_2PhSiH$ (>98%, Sigma-Aldrich), $Et_2SiH_2$ (99%, Sigma-Aldrich), $(EtO)_3SiH$ (95%, Sigma-Aldrich). PhPDI, $^{TF}$ADPI, PyBox were prepared according to previously reported procedures.[1] (*E*)-1,3-dienes were prepared by Wittig olefination[2] of the corresponding enals[3]. *E*/*Z*-1,3-dienes were prepared by Wittig olefination of the corresponding aldehydes with allyltripheylphosphonium bromide according to previously reported procedures.[4] All other reagents and solvents were purchased from commercial sources and used without purification.

$^1$H and $^{13}$C spectra were recorded using Bruker 300 MHz, 400 MHz, or 500 MHz NMR spectrometers. $^1$H NMR and $^{13}$C NMR spectra were referenced to resonances of the residual signals of the deuterated solvents. As such, the $^1$H and $^{13}$C signals of $CDCl_3$ were calibrated to 7.26 ppm (singlet) and 77.16 ppm (triplet) respectively. Multiplicities are recorded as: s = singlet, d = doublet, t = triplet, dd = doublet of doublets, dt = doublet of triplets and m = multiplet. GC analysis was acquired on Agilent 6850 gas chromatograph equipped with a flame-ionization detector. HR-MS analyses were performed using Thermo Scientific Exactive (APCI). GC-MS analysis was performed on Shimadzu GC-2010 gas chromatograph coupled to a Shimadzu QP2010 mass selective detector. The details of the methods were illustrated as below.

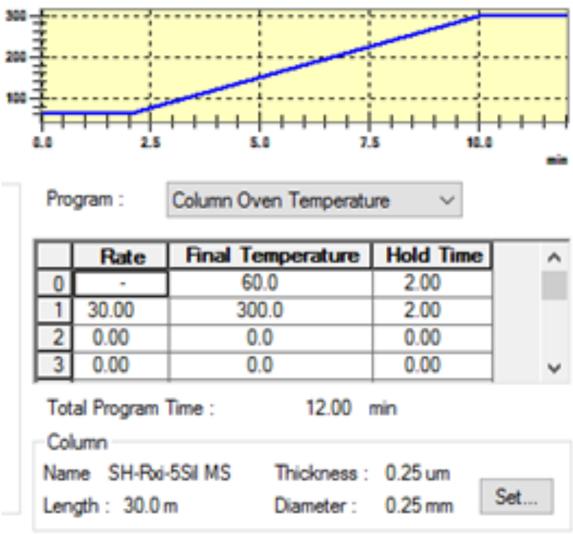


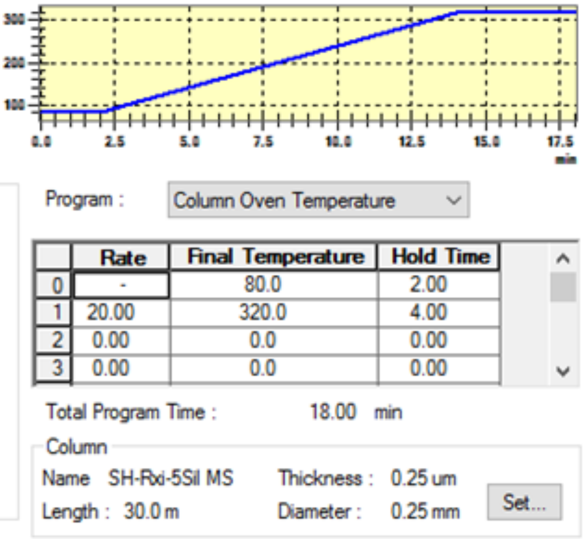

**Reaction Screening of Silane for Hydrosilylation of (*E*)-1-phenyl-1,3-butadiene**[a]

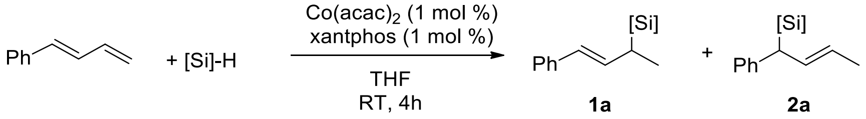


| entry | [Si]-H | Yield of **1a** (%) | **1a/4a** |
|---|---|---|---|
| 1 | $PhSiH_3$ | 80 | >99:- |
| 2 | $Ph_2SiH_2$ | 84 | >99:- |
| 3 | $Et_2SiH_2$ | - | - |
| 4[b] | $Et_2SiH_2$ | - | - |
| 5 | $MePhSiH_2$ | Trace | - |
| 6[c] | $MePhSiH_2$ | 80 | >99:1 |
| 7 | $(EtO)_3SiH$ | - | - |
| 8[b] | $(EtO)_3SiH$ | - | - |
| 9 | $Me_2PhSiH$ | - | - |
| 10[b] | $Me_2PhSiH$ | - | - |

[a]Conditions: (*E*)- 1-phenylbutadiene (0.200 mmol), [Si]-H (0.250 mmol), $Co(acac)_2$ (2.0 μmol), xantphos (2.0 μmol), THF (0.500 mL), 4 h; yield of isolated product. [b]Reactions were conducted at 50 °C instead. [c]3 mol % catalyst loading was used.

**Reaction Screening of Ligands for Asymmetric Hydrosilylation of 1-phenylbutadiene[a]**

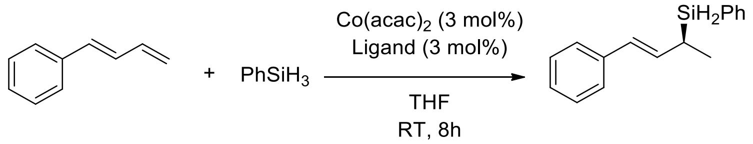


| Entry | Ligand | Conversion (%)[b] | Enantiomeric Ratio[b] |
|---|---|---|---|
| 1 | (R,R)-BDPP | >98 | 59.5:40.5 |
| 2 | (S,S)-Chiraphos | 0 | - |
| 3 | (R)-Synphos | >98 | 85:15 |
| 4 | (R)-(+)-Segphos | >98 | 86:14 |
| 5 | R-Ph-Garphos | >98 | 86:14 |
| 6 | (R)-MeO-BIPHEP | >98 | 82.5:17.5 |
| 7 | (S,S)-Norphos | 0 | - |
| 8 | (R)-(S)-Josiphos | >98 | 69:31 |
| 9 | (R,R,S,S)-Duanphos | >98 | 65:35 |
| 10 | (R)-difluorophos | 77 | 89.5:10.5 |
| 11[c] | (R)-difluorophos | >98 | 89:11 |

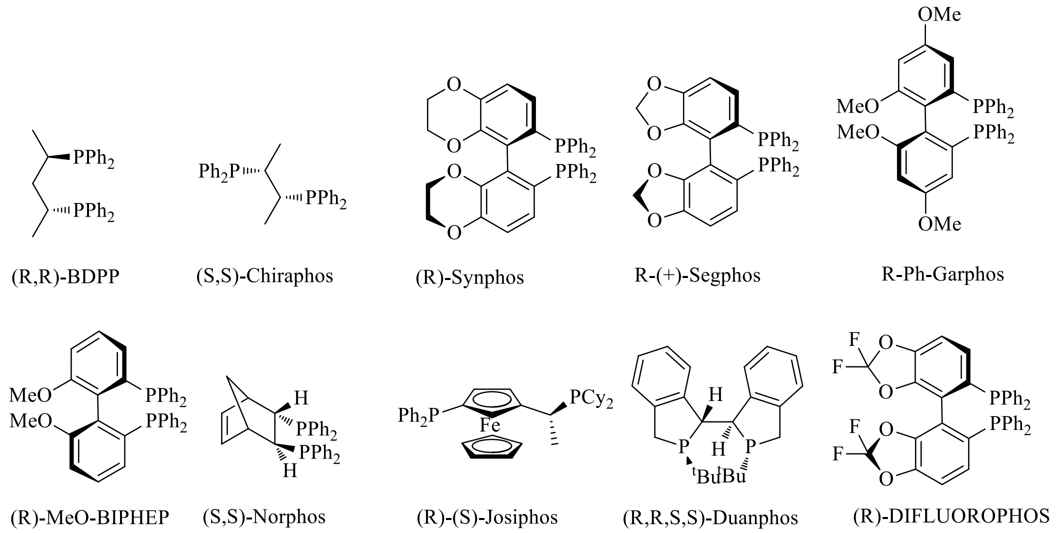


[a]Conditions: ($E$)-diene (0.200 mmol), $PhSiH_3$ (0.250 mmol), $Co(acac)_2$ (6.0 μmol), ligand (6.0 μmol), THF (0.5 mL), 8 h; [b]Conversion of diene were determined by GC analysis with tridecane as the internal standard; Enantiomeric ratio was determined by chiral HPLC. [c]Catalyst (5 mol %), 6 hours at room temperature.

## General procedure for Co-catalyzed Markovnikov 1,2-hydrosilylation of *trans*-1,3-dienes

In an Ar-filled glovebox, a mixture of $Co(acac)_2$ (1.0 mg, 4.0 $\mu$mol) and xantphos (2.3 mg, 4.0 $\mu$mol) in THF (1 mL) was added into a 4-mL screw-capped vial containing a magnetic stirring bar. The resulting mixture was stirred for 2 mins prior adding phenylsilane (54.1 mg, 0.500 mmol) and *trans*-1,3-dienes (0.400 mmol) successively. The vial was removed from the glove box, and the mixture was stirred at room temperature for 4 hours. After that, GC-MS analysis was conducted to determine the selectivity of the crude reaction mixture prior concentrating it under vacuum. Subsequently, the residue was purified by flash column chromatography using a mixture of ethyl acetate and hexane as eluent. The details and characterization data of the products are stated below.

### (*E*)-phenyl(4-phenylbut-3-en-2-yl)silane (1a)

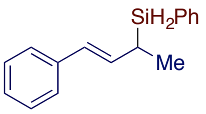


The title compound was isolated (76.3 mg, 80%, *E*/*Z* = >99:1) as colourless oil after chromatography on silica gel (100:1 hexane/EtOAc). $^1H$ NMR (400 MHz, $CDCl_3$) δ 7.63-7.58 (m, 2H), 7.47–7.36 (m, 3H), 7.34–7.28 (m, 4H), 7.23–7.17 (m, 1H), 6.35 (dd, *J* = 15.9, 6.8 Hz, 1H), 6.29 (d, *J* = 16.0 Hz, 1H), 4.35 – 4.28 (m, 2H), 2.35 – 2.23 (m, 1H), 1.32 (d, *J* = 7.2 Hz, 3H). $\{^1H\}^{13}C$ NMR (101 MHz, $CDCl_3$) δ 138.2, 135.9, 133.2, 131.30, 123.0, 128.6, 128.1, 127.4, 126.7, 125.9, 23.0, 15.3. HR-MS (APCI$^+$) *m*/*z* calcd for $C_{16}H_{18}Si$, [M+H$^+$]: 239.1256, Found: 239.1258.

The GC trace for the crude mixture of the reaction to make **1a**.

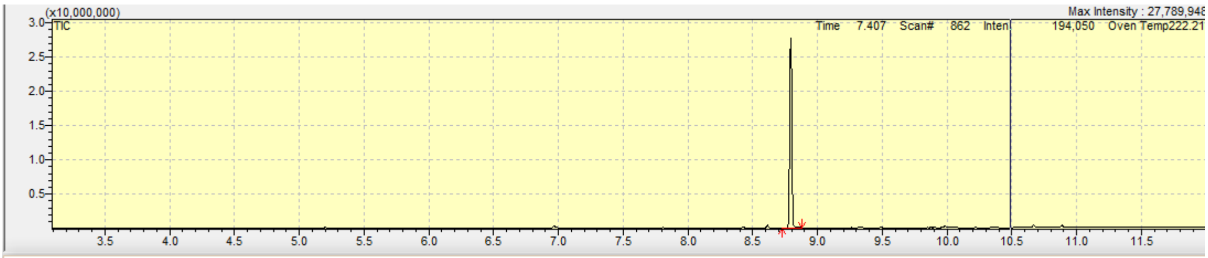


Qualitative Table

Print Edit View Similarity Search

| Peak# | Ret.Time | Start Tm | End Tm | m/z | Area | Area% | Height | Height% | A/H | Mark | Name |
|---|---|---|---|---|---|---|---|---|---|---|---|
| 1 | 8.794 | 8.725 | 8.880 | TIC | 30275580 | 100.00 | 27722943 | 100.00 | 1.09 | MI | |

### (*E*)-(4-(2-methoxyphenyl)but-3-en-2-yl)(phenyl)silane (1b)

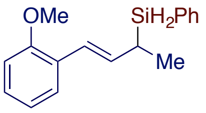


The title compound was isolated (90.2 mg, 84%, *E*/*Z* = >99:1) as colourless oil after chromatography on silica gel (20:1 hexane/EtOAc). $^1H$ NMR (400 MHz, $CDCl_3$) δ 7.63–7.58 (m, 2H), 7.44–7.35 (m, 4H), 7.20–7.14 (m, 1H), 6.94–6.84 (m, 2H), 6.64 (dd, *J* = 16.0, 1.2 Hz, 1H), 6.33 (dd, *J* = 16.0, 7.8 Hz, 1H), 4.35–4.29 (m, 2H), 3.84 (s, 3H), 2.36–2.24 (m, 1H), 1.32 (d, *J* = 7.2 Hz, 3H). $\{^1H\}^{13}C$ NMR (101 MHz, $CDCl_3$) δ 156.4, 135.9, 133.8, 131.5, 123.0, 128.1, 127.7, 127.3, 126.3, 122.1, 120.8, 111.0, 55.6, 23.5, 15.4. HR-MS (APCI$^+$) *m*/*z* calcd for $C_{17}H_{20}OSi$, [M+H$^+$]: 269.1362, Found: 269.1355.

The GC trace for the crude mixture of the reaction to make **1b**.

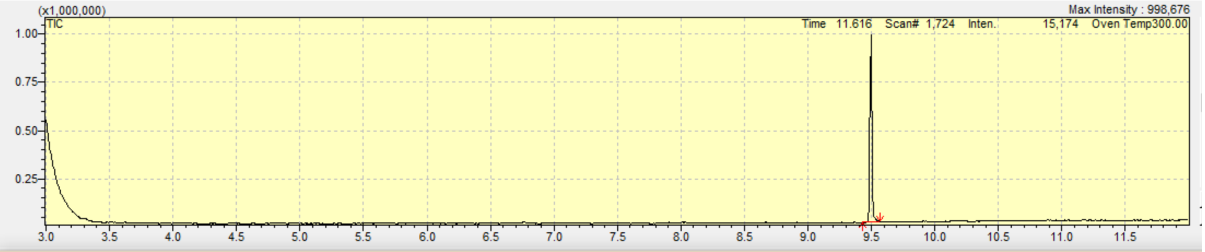


Qualitative Table

Print Edit View Similarity Search

| Peak# | Ret.Time | Start Tm | End Tm | m/z | Area | Area% | Height | Height% | A/H | Mark | Name |
|---|---|---|---|---|---|---|---|---|---|---|---|
| 1 | 9.495 | 9.430 | 9.570 | TIC | 1099705 | 100.00 | 974479 | 100.00 | 1.13 | MI | |

**(*E*)-phenyl(4-(p-tolyl)but-3-en-2-yl)silane (1c)**

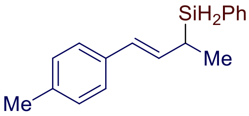


The title compound was isolated (91.9 mg, 91%, *E*/*Z* = >99:1) as colourless oil after chromatography on silica gel (100:1 hexane/EtOAc). $^{1}$H NMR (400 MHz, $CDCl_3$) δ 7.68–7.53 (m, 2H), 7.46–7.35 (m, 3H), 7.23 (d, *J* = 8.1 Hz, 2H), 7.11 (d, *J* = 7.9 Hz, 2H), 6.37–6.18 (m, 2H), 4.40–4.21 (m, 2H), 2.35 (s, 3H), 2.31–2.22 (m, 1H), 1.31 (d, *J* = 7.2 Hz, 3H). {$^{1}$H}$^{13}$C NMR (101 MHz, $CDCl_3$) δ 136.4, 135.9, 135.4, 132.1, 131.4, 129.9, 129.3, 128.1, 127.3, 125.8, 22.9, 21.3, 15.3. HR-MS (APCI$^{+}$) *m*/*z* calcd for $C_{17}H_{20}Si$, [M+H$^{+}$]: 253.4341, Found: 253.1408.

The GC trace for the crude mixture of the reaction to make **1c**.

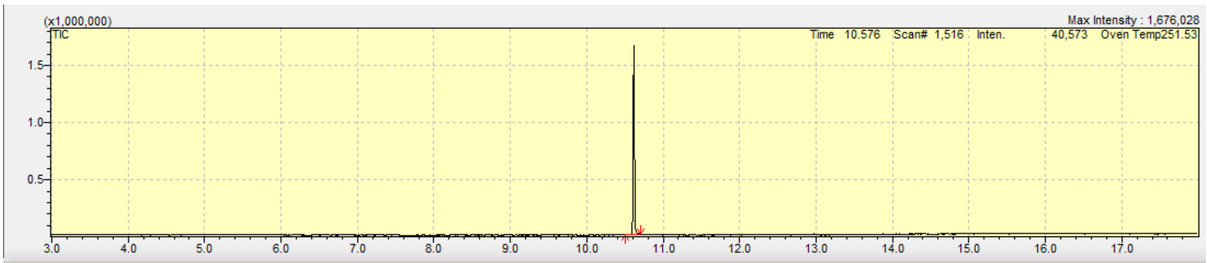


Qualitative Table

Print Edit View Similarity Search

| Peak# | Ret.Time | Start Tm | End Tm | m/z | Area | Area% | Height | Height% | A/H | Mark | Name |
|---|---|---|---|---|---|---|---|---|---|---|---|
| 1 | 10.617 | 10.505 | 10.705 | TIC | 2027429 | 100.00 | 1656446 | 100.00 | 1.22 | MI | |

**(*E*)-N,N-dimethyl-4-(3-(phenylsilyl)but-1-en-1-yl)aniline (1d)**

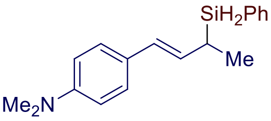


The title compound was isolated (96.7 mg, 86%, *E*/*Z* = >99:1) as colourless oil after chromatography on silica gel (20:1 hexane/EtOAc). $^{1}$H NMR (400 MHz, $CDCl_3$) δ 7.45 (dd, *J* = 7.8, 1.4 Hz, 2H), 7.29–7.17 (m, 3H), 7.08 (d, *J* = 8.7 Hz, 2H), 6.54 (d, *J* = 8.8 Hz, 2H), 6.08 (d, *J* = 15.9 Hz, 1H), 5.98 (dd, *J* = 15.8, 7.5 Hz, 1H), 4.23–4.09 (m, 2H), 2.79 (s, 6H), 2.15–2.03 (m, 1H), 1.15 (d, *J* = 7.2 Hz, 3H). {$^{1}$H}$^{13}$C NMR (101 MHz, $CDCl_3$) δ 149.7, 135.9, 131.7, 129.8, 128.9, 128.0, 127.3, 127.1, 126.7, 112.8, 40.8, 22.7, 15.5. HR-MS (APCI$^{+}$) *m*/*z* calcd for $C_{18}H_{24}NSi$, [M+H$^{+}$]: 282.1678, Found: 282.1681.

The GC trace for the crude mixture of the reaction to make **1d**.

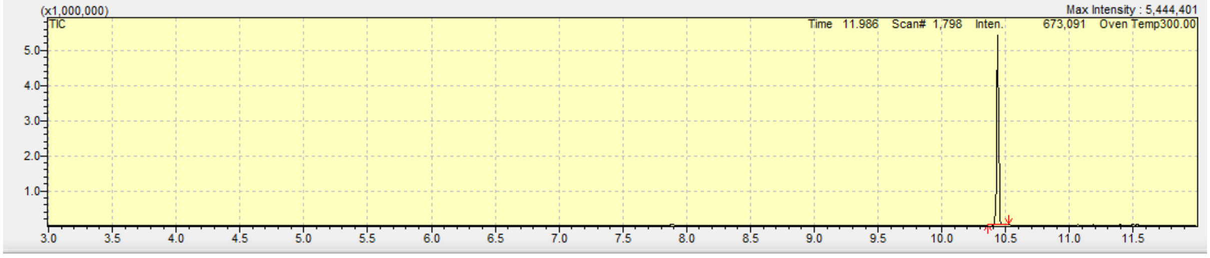


Qualitative Table

Print Edit View Similarity Search

| Peak# | Ret.Time | Start Tm | End Tm | m/z | Area | Area% | Height | Height% | A/H | Mark | Name |
|---|---|---|---|---|---|---|---|---|---|---|---|
| 1 | 10.437 | 10.360 | 10.525 | TIC | 5886890 | 100.00 | 5413328 | 100.00 | 1.09 | MI | |

**(*E*)-phenyl(4-(4-(trifluoromethyl)phenyl)but-3-en-2-yl)silane (1e)**

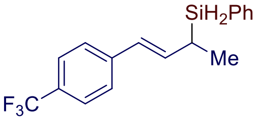


The title compound was isolated (108 mg, 88%, *E*/*Z* = >99:1) as colourless oil after chromatography on silica gel (50:1 hexane/EtOAc). $^{1}$H NMR (400 MHz, $CDCl_3$) δ 7.62–7.58 (m, 2H), 7.55 (d, *J* = 8.2 Hz, 2H), 7.48–7.43 (m, 1H), 7.43–7.37 (m, 4H), 6.47 (dd, *J* = 15.9, 7.7 Hz, 1H), 6.31 (d, *J* = 16.3 Hz, 1H), 4.35 (d, *J* = 2.9 Hz, 2H), 2.39–2.29 (m, 1H), 1.35 (d, *J* = 7.1 Hz, 3H). {$^{1}$H}$^{13}$C NMR (101 MHz, $CDCl_3$) δ 141.7 (d, $J_{C\text{-}F}$ = 1.2 Hz), 136.2, 135.8, 130.9, 130.1, 128.5 (q, $J_{C\text{-}F}$ = 32.4 Hz), 128.2, 126.2, 126.0, 125.6 (q, $J_{C\text{-}F}$ = 3.8 Hz), 124.5 (q, $J_{C\text{-}F}$ = 271.7 Hz), 23.4, 15.1. $^{19}$F NMR (377 MHz, $CDCl_3$) δ -62.30. GC-MS (EI) *m*/*z* calcd for $C_{17}H_{18}F_3Si$ [M]$^{+}$: 306.40, Found: 306.15

The GC trace for the crude mixture of the reaction to make **1e**.

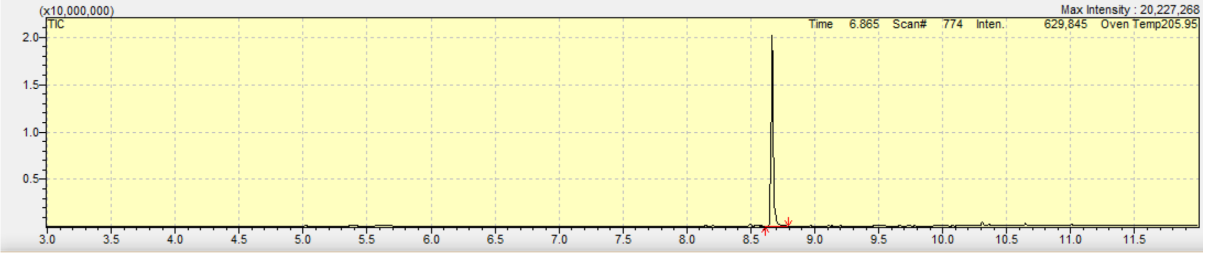


Qualitative Table

Print Edit View Similarity Search

| Peak# | Ret.Time | Start Tm | End Tm | m/z | Area | Area% | Height | Height% | A/H | Mark | Name |
|---|---|---|---|---|---|---|---|---|---|---|---|
| 1 | 8.665 | 8.610 | 8.795 | TIC | 24923902 | 100.00 | 20140224 | 100.00 | 1.24 | MI | |

**(*E*)-(4-(4-bromophenyl)but-3-en-2-yl)(phenyl)silane (1f)**

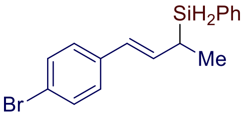


The title compound was isolated (101 mg, 80%, *E*/*Z* = >99:1) as colourless oil after chromatography on silica gel (100:1 hexane/EtOAc). $^1$H NMR (400 MHz, $CDCl_3$) δ 7.62–7.54 (m, 2H), 7.47–7.31 (m, 5H), 7.21–7.10 (m, 2H), 6.33 (dd, $J$ = 15.9, 7.6 Hz, 1H), 6.20 (d, $J$ = 16.0 Hz, 1H), 4.39–4.22 (m, 2H), 2.41–2.17 (m, 1H), 1.31 (d, $J$ = 7.2 Hz, 3H). {$^1$H}$^{13}$C NMR (101 MHz, $CDCl_3$) δ 137.1, 135.8, 134.1, 131.7, 131.1, 130.0, 128.1, 127.4, 126.2, 120.3, 23.2, 15.1. HR-MS (APCI$^+$) *m*/*z* calcd for $C_{16}H_{18}BrSi$, [M+H$^+$]: 317.0361, Found: 317.0357.

The GC trace for the crude mixture of the reaction to make **1f**.

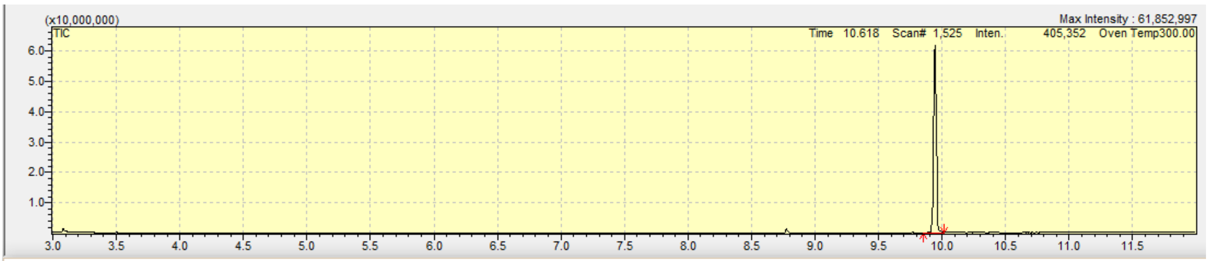


Qualitative Table

Print Edit View Similarity Search

| Peak# | Ret.Time | Start Tm | End Tm | m/z | Area | Area% | Height | Height% | A/H | Mark | Name |
|---|---|---|---|---|---|---|---|---|---|---|---|
| 1 | 9.944 | 9.850 | 10.015 | TIC | 98848339 | 100.00 | 61674826 | 100.00 | 1.60 | MI | |

**(*E*)-2-methoxy-4-(3-(phenylsilyl)but-1-en-1-yl)phenyl acetate (1g)**

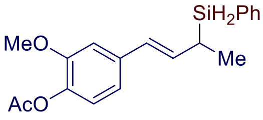


The title compound was isolated (104 mg, 80%, *E*/*Z* = >99:1) as colourless oil after chromatography on silica gel (10:1 hexane/EtOAc). $^1$H NMR (400 MHz, $CDCl_3$) δ 7.60–7.54 (m, 2H), 7.45–7.33 (m, 3H), 6.94 (d, $J$ = 8.3 Hz, 1H), 6.89–6.84 (m, 2H), 6.29–6.19 (m, 2H), 4.30 (d, $J$ = 2.9 Hz, 2H), 3.83 (s, 3H), 2.31 (s, 3H), 2.28–2.22 (m, 1H), 1.29 (d, $J$ = 7.2 Hz, 3H). {$^1$H}$^{13}$C NMR (101 MHz, $CDCl_3$) δ 169.3, 151.2, 138.7, 137.3, 135.9, 133.6, 131.2, 130.0, 128.1, 126.8, 122.8, 118.4, 109.8, 56.0, 23.0, 20.8, 15.2. GC-MS (EI) *m*/*z* calcd for $C_{19}H_{22}O_3Si$ [M]$^+$: 326.13, Found: 326.20

The GC trace for the crude mixture of the reaction to make **1g**.

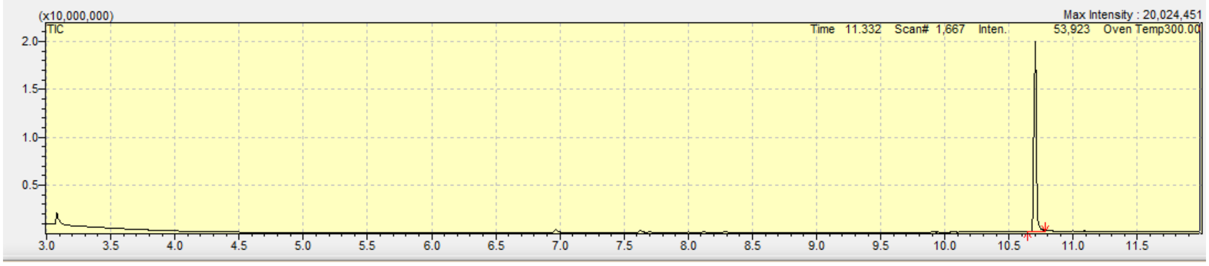


Qualitative Table

Print Edit View Similarity Search

| Peak# | Ret.Time | Start Tm | End Tm | m/z | Area | Area% | Height | Height% | A/H | Mark | Name |
|---|---|---|---|---|---|---|---|---|---|---|---|
| 1 | 10.702 | 10.645 | 10.780 | TIC | 27168811 | 100.00 | 19850716 | 100.00 | 1.37 | MI | |

**(*E*)-(4-(furan-2-yl)but-3-en-2-yl)(phenyl)silane (1h)**

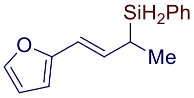


The title compound was isolated (78.6 mg, 86%, *E*/*Z* = >99:1) as pale yellow oil after chromatography on silica gel (100:1 hexane/EtOAc). $^{1}$H NMR (400 MHz, $CDCl_3$) δ 7.62–7.54 (m, 2H), 7.44–7.33 (m, 3H), 7.30 (d, *J* = 1.5 Hz, 1H), 6.38–6.25 (m, 2H), 6.14–6.03 (m, 2H), 4.29 (qd, *J* = 6.7, 3.0 Hz, 2H), 2.28–2.17 (m, 1H), 1.27 (d, *J* = 7.2 Hz, 3H). {$^{1}$H}$^{13}$C NMR (101 MHz, $CDCl_3$) δ 153.7, 141.2, 135.8, 132.2, 131.2, 123.0, 128.1, 116.2, 111.2, 105.7, 22.9, 15.0. GC-MS (EI) *m*/*z*: calcd for $C_{14}H_{16}OSi$ $[M]^+$: 228.36, Found: 228.10.

The GC trace for the crude mixture of the reaction to make **1h**.

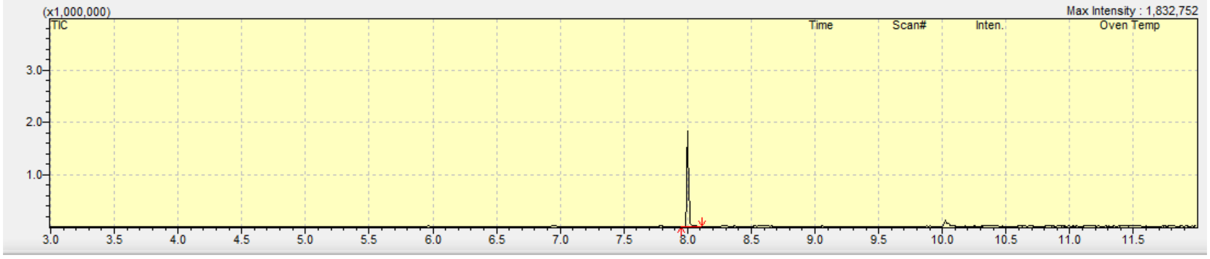


| Peak# | Ret.Time | Start Tm | End Tm | m/z | Area | Area% | Height | Height% | A/H | Mark | Name |
|---|---|---|---|---|---|---|---|---|---|---|---|
| 1 | 8.001 | 7.950 | 8.115 | TIC | 1895901 | 100.00 | 1819889 | 100.00 | 1.04 | MI | |

**(*E*)-dodec-3-en-2-yl(phenyl)silane (1i)**

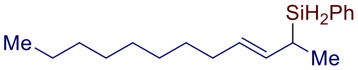


The title compound was isolated (48 mg, 87%, *E*/*Z* = >99:1) as colourless oil after chromatography on silica gel (100:0 hexane/EtOAc). $^{1}$H NMR (400 MHz, $CDCl_3$) δ 7.60–7.55 (m, 2H), 7.44–7.33 (m, 3H), 5.54–5.45 (m, 1H), 5.36–5.27 (m, 1H), 4.29–4.17 (m, 2H), 2.00 (m, 3H), 1.34–1.25 (m, 12H), 1.18 (d, *J* = 7.3 Hz, 3H), 0.91 (t, *J* = 6.9 Hz, 3H). {$^{1}$H}$^{13}$C NMR (101 MHz, $CDCl_3$) δ 135.8, 132.0, 131.9, 129.7, 128.5, 128.0, 32.9, 32.1, 30.0, 29.7, 29.5, 29.3, 22.9, 21.8, 15.6, 14.3. GC-MS (EI) *m*/*z*: calcd for $C_{18}H_{30}Si$ $[M]^+$: 274.21; Found: 274.30.

The GC trace for the crude mixture of the reaction to make **1i**.

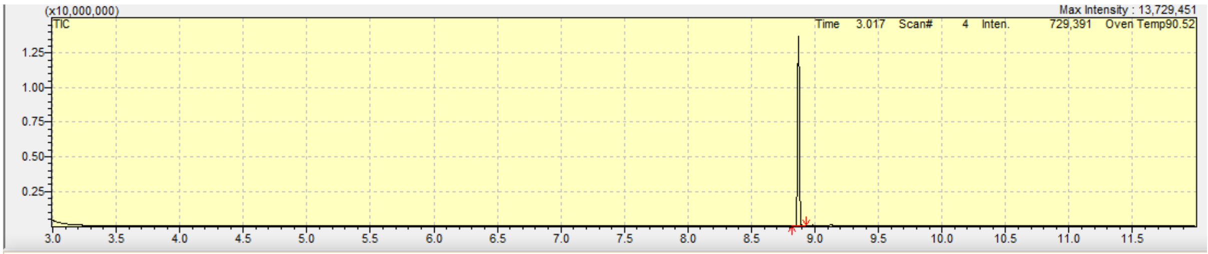


| Peak# | Ret.Time | Start Tm | End Tm | m/z | Area | Area% | Height | Height% | A/H | Mark | Name |
|---|---|---|---|---|---|---|---|---|---|---|---|
| 1 | 8.870 | 8.820 | 8.930 | TIC | 12272457 | 100.00 | 13701018 | 100.00 | 0.90 | MI | |

**(*E*)-non-3-en-2-yl(phenyl)silane (1j)**

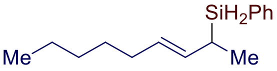


The title compound was isolated (71.0 mg, 76%, *E*/*Z* = >99:1) as colourless oil after chromatography on silica gel (100:0 hexane/EtOAc). $^{1}$H NMR (400 MHz, $CDCl_3$) δ 7.56 (dd, *J* = 7.8, 1.5 Hz, 2H), 7.42–7.33 (m, 3H), 5.52–5.45 (m, 1H), 5.34–5.27 (m, 1H), 4.24–4.19 (m, 2H), 2.05–1.97 (m, 3H), 1.35–1.23 (m, 6H), 1.17 (d, *J* = 7.3 Hz, 3H), 0.88 (d, *J* = 7.2 Hz, 3H). {$^{1}$H}$^{13}$C NMR (101 MHz, $CDCl_3$) δ 135.8, 132.0, 131.9, 129.7, 128.5, 128.0, 32.9, 31.5, 29.6, 22.7, 21.8, 15.6, 14.2. GC-MS (EI) *m*/*z*: calcd for $C_{15}H_{24}Si$ $[M]^+$: 232.16; Found: 232.20.

The GC trace for the crude mixture of the reaction to make **1j**.

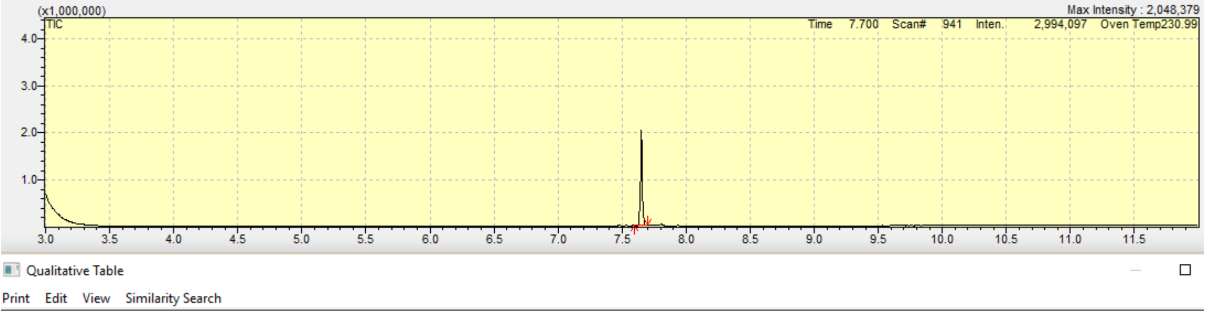


| Peak# | Ret.Time | Start Tm | End Tm | m/z | Area | Area% | Height | Height% | A/H | Mark | Name |
|---|---|---|---|---|---|---|---|---|---|---|---|
| 1 | 7.651 | 7.595 | 7.700 | TIC | 2017779 | 100.00 | 2015292 | 100.00 | 1.00 | MI | |

**(3*E*,7*Z*)-deca-3,7-dien-2-yl(phenyl)silane (1k)**

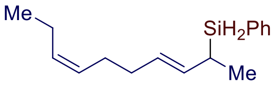


The title compound was isolated (62 mg, 64%, *E*/*Z* = >99:1) as colourless oil after chromatography on silica gel (100:0 hexane/EtOAc). $^{1}$H NMR (400 MHz, $CDCl_3$) δ 7.57 (dd, *J* = 7.8, 1.5 Hz, 2H), 7.44–7.33 (m, 3H), 5.53 (dd, *J* = 15.3, 7.4 Hz, 1H), 5.45–5.26 (m, 3H), 4.26–4.18 (m, 2H), 2.11–1.97 (m, 7H), 1.18 (d, *J* = 7.3 Hz, 3H), 0.97 (t, *J* = 7.5 Hz, 3H). {$^{1}$H}$^{13}$C NMR (101 MHz, $CDCl_3$) δ 135.8, 132.5, 132.0, 131.9, 129.8, 128.7, 128.0, 127.7, 33.1, 27.6, 21.8, 20.7, 15.5, 14.5. GC-MS (EI) *m*/*z*: calcd for $C_{16}H_{24}Si$ $[M]^{+}$: 244.16; Found: 244.20.

The GC trace for the crude mixture of the reaction to make **1k**.

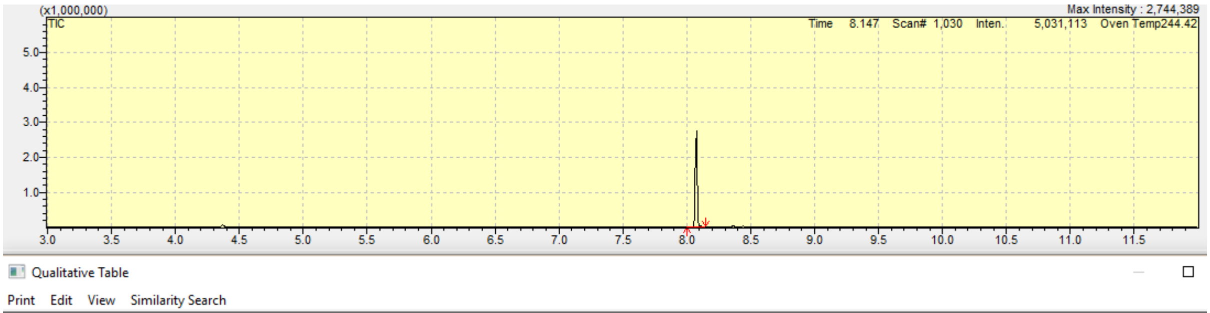


| Peak# | Ret.Time | Start Tm | End Tm | m/z | Area | Area% | Height | Height% | A/H | Mark | Name |
|---|---|---|---|---|---|---|---|---|---|---|---|
| 1 | 8.074 | 8.000 | 8.145 | TIC | 2750405 | 100.00 | 2728196 | 100.00 | 1.01 | MI | |

**(*E*)-(2-methyl-4-phenylbut-3-en-2-yl)(phenyl)silane (1l)**

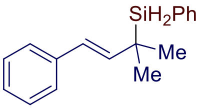


The title compound was isolated (71.0 mg, 70%, *E*/*Z* = >99:1) as colourless oil after chromatography on silica gel (100:1 hexane/EtOAc). $^{1}$H NMR (400 MHz, $CDCl_3$) δ 7.57 (dd, *J* = 7.9, 1.4 Hz, 2H), 7.47–7.24 (m, 7H), 7.22–7.16 (m, 1H), 6.34 (d, *J* = 16.1 Hz, 1H), 6.18 (d, *J* = 16.1 Hz, 1H), 4.25 (s, 2H), 1.27 (m, 6H). {$^{1}$H}$^{13}$C NMR (101 MHz, $CDCl_3$) δ 138.3, 138.3, 136.2, 131.4, 130.0, 128.6, 128.0, 126.8, 126.1, 126.0, 25.7, 24.1. HR-MS(APCI$^{+}$) *m*/*z* calcd for $C_{17}H_{20}Si$, $[M+H^{+}]$: 253.1413, Found:253.1416.

The GC trace for the crude mixture of the reaction to make **1l**.

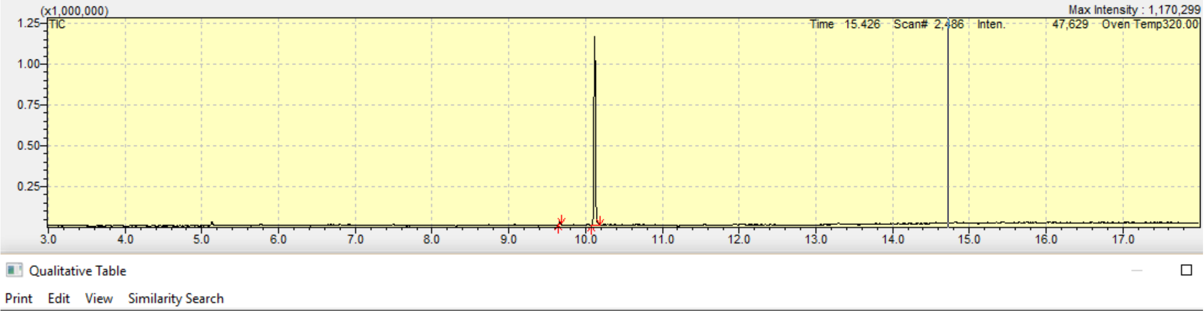


| Peak# | Ret.Time | Start Tm | End Tm | m/z | Area | Area% | Height | Height% | A/H | Mark | Name |
|---|---|---|---|---|---|---|---|---|---|---|---|
| 1 | 9.668 | 9.650 | 9.680 | TIC | 17402 | 1.22 | 16896 | 1.44 | 1.03 | MI | |
| 2 | 10.119 | 10.070 | 10.190 | TIC | 1409699 | 98.78 | 1157047 | 98.56 | 1.22 | MI | |

**(4,4-diphenylbut-3-en-2-yl)(phenyl)silane (1m)**

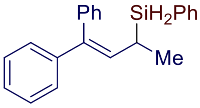


The title compound was isolated (106 mg, 84%, *E*/*Z* = >99:1) as colourless oil after chromatography on silica gel (100:1 hexane/EtOAc). $^{1}$H NMR (400 MHz, $CDCl_3$) δ 7.54–

7.49 (m, 2H), 7.45–7.27 (m, 6H), 7.27–7.16 (m, 5H), 7.08–7.02 (m, 2H), 6.02 (d, *J* = 11.5 Hz, 1H), 4.33 – 4.22 (m, 2H), 2.37 – 2.24 (m, 1H), 1.25 (d, *J* = 7.1 Hz, 3H). {$^1$H}$^{13}$C NMR (101 MHz, $CDCl_3$) δ 142.7, 140.4, 134.0, 135.9, 132.1, 131.4, 123.0, 129.9, 128.4, 128.2, 128.1, 127.1, 126.9, 126.8, 20.8, 16.9. HR-MS (APCI$^+$) *m/z* calcd for $C_{22}H_{23}Si$, [M+H$^+$]: 315.1569, Found: 315.1566.

The GC trace for the crude mixture of the reaction to make **1m**.

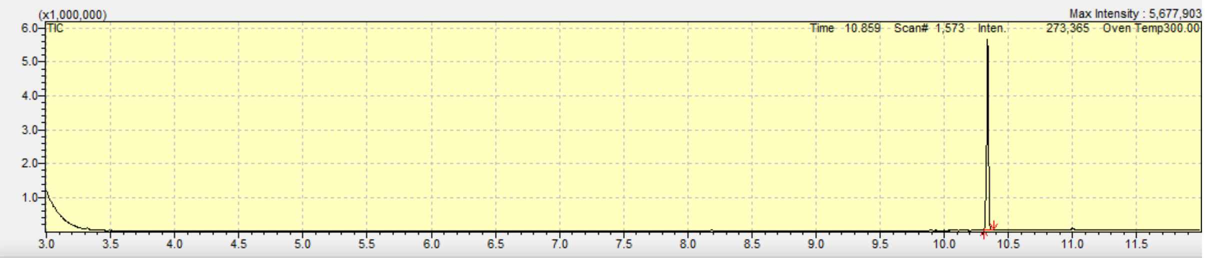


Qualitative Table

Print Edit View Similarity Search

| Peak# | Ret.Time | Start Tm | End Tm | m/z | Area | Area% | Height | Height% | A/H | Mark | Name |
|---|---|---|---|---|---|---|---|---|---|---|---|
| 1 | 10.337 | 10.310 | 10.385 | TIC | 6123524 | 100.00 | 5640650 | 100.00 | 1.09 | MI | |

**(*E*)-(3-methyl-4-phenylbut-3-en-2-yl)(phenyl)silane (1n)**

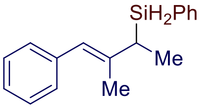


The title compound was isolated (93.0 mg, 92%, *E/Z* = >99:1) as colourless oil after chromatography on silica gel (100:1 hexane/EtOAc). $^1$H NMR (400 MHz, $CDCl_3$) δ 7.60 (d, *J* = 6.5 Hz, 2H), 7.46–7.27 (m, 5H), 7.19 (d, *J* = 6.9 Hz, 3H), 6.15 (s, 1H), 4.37 (m, 2H), 2.21–2.12 (m, 1H), 1.90 (s, 3H), 1.34 (d, *J* = 7.3 Hz, 3H). {$^1$H}$^{13}$C NMR (101 MHz, $CDCl_3$) δ 141.3, 139.0, 135.8, 132.0, 129.9, 129.0, 128.1, 128.1, 125.8, 123.5, 29.4, 18.8, 15.6. HR-MS (APCI$^+$) *m/z* calcd for $C_{17}H_{20}Si$, [M+H$^+$]: 253.1413, Found: 253.1413.

The GC trace for the crude mixture of the reaction to make **1n**.

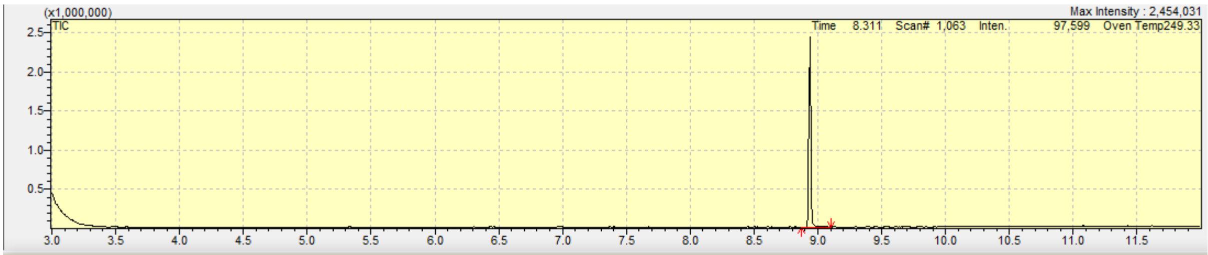


Qualitative Table

Print Edit View Similarity Search

| Peak# | Ret.Time | Start Tm | End Tm | m/z | Area | Area% | Height | Height% | A/H | Mark | Name |
|---|---|---|---|---|---|---|---|---|---|---|---|
| 1 | 8.936 | 8.870 | 9.105 | TIC | 2586876 | 100.00 | 2434940 | 100.00 | 1.06 | MI | |

**(*E*)-diphenyl(4-phenylbut-3-en-2-yl)silane (1a')**

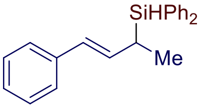


The title compound was isolated (106 mg, 84%, *E/Z* = >99:1) as colourless oil after chromatography on silica gel (100:1 hexane/EtOAc). $^1$H NMR (400 MHz, $CDCl_3$) δ 7.67–7.58 (m, 4H), 7.46–7.32 (m, 6H), 7.27 (m, 3H), 7.24–7.11 (m, 2H), 6.36 (dd, *J* = 15.9, 7.4 Hz, 1H), 6.25 (dd, *J* = 16.0, 0.9 Hz, 1H), 4.83 (d, *J* = 2.8 Hz, 1H), 2.57–2.44 (m, 1H), 1.33 (d, *J* = 7.2 Hz, 3H). {$^1$H}$^{13}$C NMR (101 MHz, $CDCl_3$) δ 138.4, 135.8, 135.7, 134.5, 133.2, 133.1, 133.0, 130.0, 128.6, 128.1, 128.1, 127.6, 126.6, 125.9, 24.37, 14.9. HR-MS (APCI$^+$) *m/z* calcd for $C_{22}H_{23}Si$, [M+H$^+$]: 315.1569, Found: 315.1555.

The GC trace for the crude mixture of the reaction to make **1a'**.

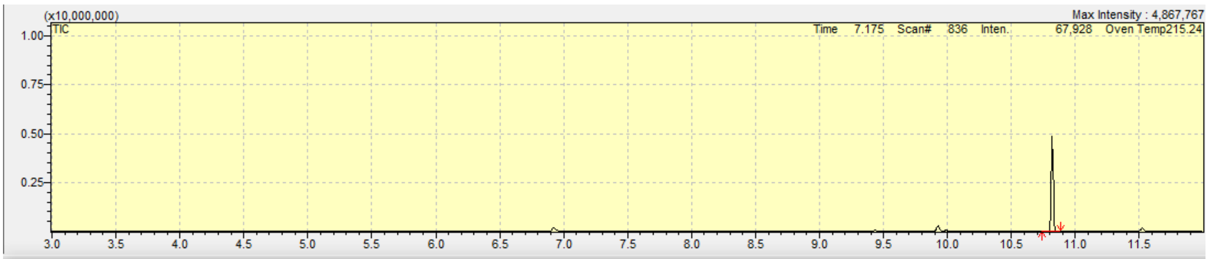


Qualitative Table

Print Edit View Similarity Search

| Peak# | Ret.Time | Start Tm | End Tm | m/z | Area | Area% | Height | Height% | A/H | Mark | Name |
|---|---|---|---|---|---|---|---|---|---|---|---|
| 1 | 10.820 | 10.740 | 10.885 | TIC | 5333527 | 100.00 | 4844821 | 100.00 | 1.10 | MI | |

**(*E*)-Methyl(phenyl)(4-phenylbut-3-en-2-yl)silane (1a'')**

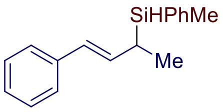


The reaction was conducted with 3 mol % catalyst loading at 0.2 mmol scale. The title compound was isolated (40.3 mg, 80%, *E/Z* = >99:1 dr = 1:1 determined by $^1$H NMR analysis) as colourless oil after chromatography on silica gel (100:0 hexane/EtOAc). $^1$H NMR (400 MHz, $CDCl_3$) δ 7.58–7.54 (m, 2H), 7.44–7.35 (m, 3H), 7.34–7.27 (m, 4H), 7.21–7.16 (m, 1H), 6.35–6.27 (m, 1H), 6.24 (d, *J* = 16.0 Hz, 1H), 4.38–4.30 (m, 1H), 2.20–2.10 (m, 1H), 1.27–1.22 (m, 3H), 0.40 (t, *J* = 3.6 Hz, 3H).{$^1$H}$^{13}$C NMR (101 MHz, $CDCl_3$) δ 138.4, 135.0, 135.0, 134.9, 134.8, 133.6, 133.5, 129.6, 128.6, 128.0, 128.0, 127.0, 126.9, 126.6, 125.8, 25.3, 25.2, 14.6, 14.2, -7.5, -7.7. GC-MS (EI) *m/z*: calcd for $C_{17}H_{20}Si$ $[M]^+$: 252.43; Found: 252.15.

The GC trace for the crude mixture of the reaction to make **1a''**.

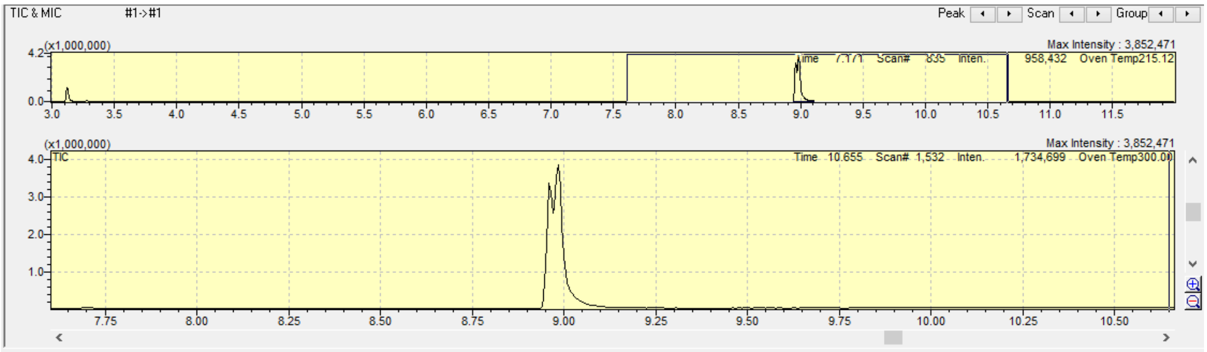


**(*E*)-(4-(4-methoxyphenyl)but-3-en-2-yl)(phenyl)silane**

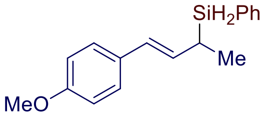


The title compound was isolated (96.0 mg, 90%, *E/Z* = >99:1.) as colourless oil after chromatography on silica gel (100:1 hexane/EtOAc). $^1$H NMR (400 MHz, $CDCl_3$) δ 7.62–7.51 (m, 2H), 7.45–7.31 (m, 3H), 7.26–7.22 (m, 2H), 6.85–6.79 (m, 2H), 6.23 (d, *J* = 16.0 Hz, 1H), 6.16 (dd, *J* = 15.9, 6.9 Hz, 1H), 4.35–4.19 (m, 2H), 3.80 (s, 3H), 2.28–2.19 (m, 1H), 1.28 (d, *J* = 7.2 Hz, 3H). {$^1$H}$^{13}$C NMR (101 MHz, $CDCl_3$) δ 158.7, 135.9, 131.5, 131.1, 131.0, 129.9, 128.1, 127.0, 126.8, 114.1, 55.5, 22.8, 15.4. HR-MS (APCI $^+$) *m/z* calcd for $C_{17}H_{21}OSi$, $[M+H^+]$: 269.1362, Found: 269.1354.

The GC trace for the crude mixture of the reaction to make this compound.

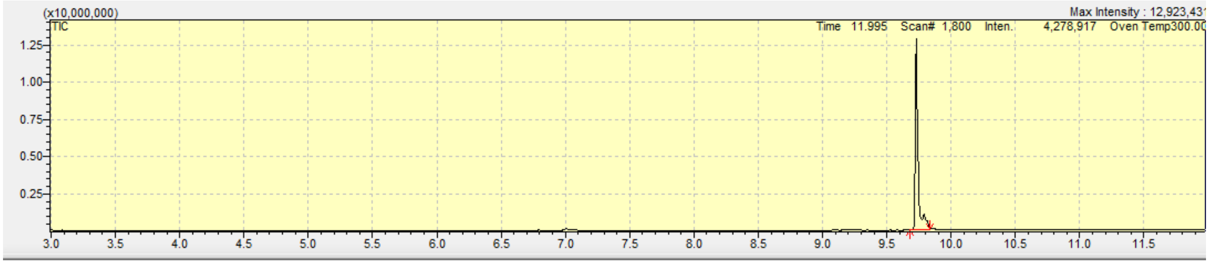


Qualitative Table

Print Edit View Similarity Search

| Peak# | Ret.Time | Start Tm | End Tm | m/z | Area | Area% | Height | Height% | A/H | Mark | Name |
|---|---|---|---|---|---|---|---|---|---|---|---|
| 1 | 9.730 | 9.680 | 9.835 | TIC | 19452923 | 100.00 | 12824234 | 100.00 | 1.52 | MI | |

**General procedure for Co-catalyzed Markovnikov 1,2-hydrosilylation of *trans/cis*-1,3-dienes mixture**

In an Ar-filled glovebox, a mixture of $Co(acac)_2$ (1.0 mg, 4.0 $\mu$mol) and xantphos (2.3 mg, 4.0 $\mu$mol) in THF (1 mL) was added into a 4-mL screw-capped vial containing a magnetic stirring bar. The resulting mixture was stirred for 2 mins prior adding phenylsilane (54.1 mg ,0.500 mmol) and *trans/cis*-1,3-dienes (0.400 mmol) successively. The vial was removed from the glove box, and the mixture was stirred at room temperature for 6 hours or 5 ºC for 24 hours. After that, GC-MS analysis was conducted to determine the selectivity of the crude reaction mixture prior concentrating it under vacuum. The residue was then purified by flash column chromatography using a mixture of ethyl acetate and hexane as eluent. The details and characterization data of the products are stated below.

**(*E*)-phenyl(4-phenylbut-3-en-2-yl)silane (1a)**

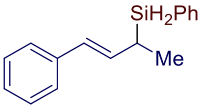


The reaction was stirred for 6 h at 24 ˚C. The title compound was isolated (76.3 mg, 80%, *E/Z* = >99:1, 1,2/1,4 = 95:5) as colourless oil after chromatography on silica gel (100:1 hexane/EtOAc). $^1$H NMR (400 MHz, $CDCl_3$) δ 7.63–7.58 (m, 2H), 7.47–7.36 (m, 3H), 7.34–7.28 (m, 4H), 7.23–7.17 (m, 1H), 6.35 (dd, *J* = 15.9, 6.8 Hz, 1H), 6.29 (d, *J* = 16.0 Hz, 1H), 4.35–4.28 (m, 2H), 2.35–2.23 (m, 1H), 1.32 (d, *J* = 7.2 Hz, 3H). {$^1$H}$^{13}$C NMR (101 MHz, $CDCl_3$) δ 138.2, 135.9, 133.2, 131.3, 123.0, 128.6, 128.1, 127.4, 126.7, 125.9, 23.0, 15.3. HR-MS (APCI $^+$) *m/z* calcd for $C_{16}H_{18}Si$, [M+H$^+$]: 239.1256, Found: 239.1258.

The GC trace for the crude mixture of the reaction to make **1a**.

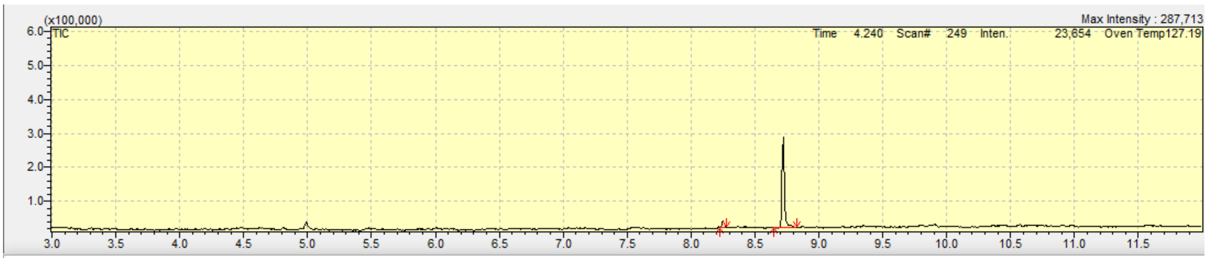


Qualitative Table

Print Edit View Similarity Search

| Peak# | Ret.Time | Start Tm | End Tm | m/z | Area | Area% | Height | Height% | A/H | Mark | Name |
|---|---|---|---|---|---|---|---|---|---|---|---|
| 1 | 8.249 | 8.225 | 8.275 | TIC | 19534 | 5.31 | 16449 | 5.86 | 1.19 | MI | |
| 2 | 8.720 | 8.645 | 8.825 | TIC | 348544 | 94.69 | 264461 | 94.14 | 1.32 | MI | |

**(*E*)-phenyl(4-(p-tolyl)but-3-en-2-yl)silane (1c)**

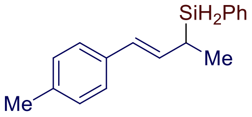


The reaction was stirred for 24 h at 5 ˚C. The title compound was isolated (77.7 mg, 77%, *E/Z* = >99:1, 1.2/1,4= 95:5) as colourless oil after chromatography on silica gel (100:1 hexane/EtOAc). $^1$H NMR (400 MHz, $CDCl_3$) δ 7.68–7.53 (m, 2H), 7.46–7.35 (m, 3H), 7.23 (d, *J* = 8.1 Hz, 2H), 7.11 (d, *J* = 7.9 Hz, 2H), 6.28 (m, 2H), 4.40–4.21 (m, 2H), 2.35 (s, 3H), 2.31–2.22 (m, 1H), 1.31 (d, *J* = 7.2 Hz, 3H). {$^1$H}$^{13}$C NMR (101 MHz, $CDCl_3$) δ 136.4, 135.9, 135.4, 132.1, 131.4, 129.9, 129.3, 128.1, 127.3, 125.8, 22.9, 21.3, 15.3. HR-MS (APCI$^+$) *m/z* calcd for $C_{17}H_{20}Si$, [M+H$^+$]: 253.4341, Found: 253.1408.

The GC trace for the crude mixture of the reaction to make **1c**.

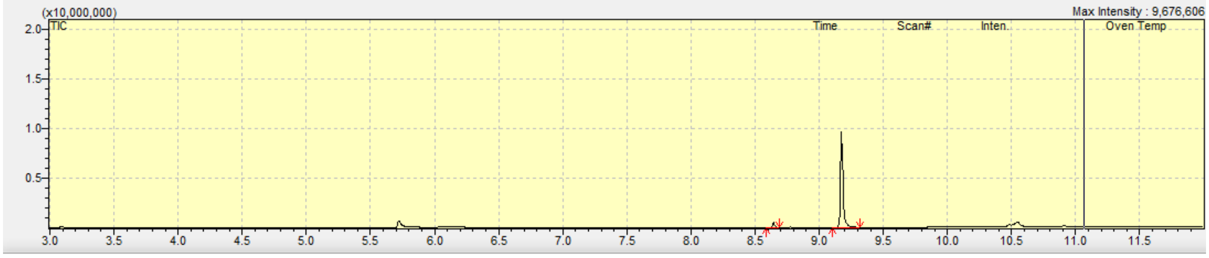


Qualitative Table

Print Edit View Similarity Search

| Peak# | Ret.Time | Start Tm | End Tm | m/z | Area | Area% | Height | Height% | A/H | Mark | Name |
|---|---|---|---|---|---|---|---|---|---|---|---|
| 1 | 8.645 | 8.585 | 8.690 | TIC | 703495 | 4.95 | 600521 | 5.88 | 1.17 | MI | |
| 2 | 9.174 | 9.100 | 9.320 | TIC | 13497263 | 95.05 | 9609546 | 94.12 | 1.40 | MI | |

**(*E*)-phenyl(4-(4-(trifluoromethyl)phenyl)but-3-en-2-yl)silane (1e)**

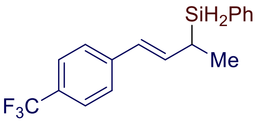


The reaction was stirred for 6 h at 24 ˚C. The title compound was isolated (79.0 mg, 65%, *E/Z* = >99:1, 1.2/1,4= 99:1) as colourless oil after chromatography on silica gel (50:1 hexane/EtOAc). $^1$H NMR (400 MHz, $CDCl_3$) δ 7.62–7.58 (m, 2H), 7.55 (d, *J* = 8.2 Hz, 2H), 7.48–7.43 (m, 1H), 7.43–7.37 (m, 4H), 6.47 (dd, *J* = 15.9, 7.7 Hz, 1H), 6.31 (d, *J* = 16.3 Hz, 1H), 4.35 (d, *J* = 2.9 Hz, 2H), 2.39 – 2.29 (m, 1H), 1.35 (d, *J* = 7.1 Hz, 3H). {$^1$H}$^{13}$C NMR (101 MHz, $CDCl_3$) δ 141.7 (d, $J_{C\text{-}F}$ = 1.2 Hz), 136.2, 135.8, 130.9, 130.1, 128.5 (q, $J_{C\text{-}F}$ = 32.4 Hz), 128.2, 126.2, 126.0, 125.6 (q, $J_{C\text{-}F}$ = 3.8 Hz), 124.5 (q, $J_{C\text{-}F}$ = 271.7 Hz), 23.4, 15.1. $^{19}$F NMR (377 MHz, $CDCl_3$) δ -62.30. GC-MS (EI) *m/z* calcd for $C_{17}H_{18}F_3Si$ [M]$^+$: 306.40, Found: 306.15

The GC trace for the crude mixture of the reaction to make **1e**.

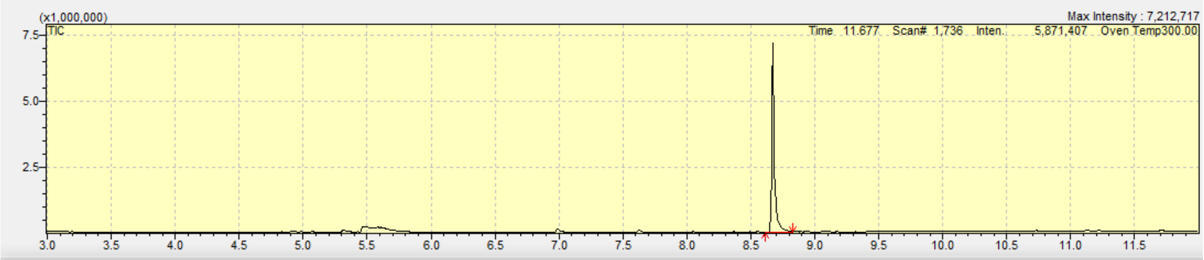


Qualitative Table

Print Edit View Similarity Search

| Peak# | Ret.Time | Start Tm | End Tm | m/z | Area | Area% | Height | Height% | A/H | Mark | Name |
|---|---|---|---|---|---|---|---|---|---|---|---|
| 1 | 8.671 | 8.610 | 8.825 | TIC | 10871644 | 100.00 | 7168690 | 100.00 | 1.52 | MI | |

**(*E*)-(4-(4-bromophenyl)but-3-en-2-yl)(phenyl)silane (1f)**

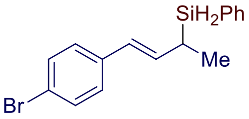


The reaction was stirred for 6 h at 24 ˚C. The title compound was isolated (66.0 mg, 52%, *E/Z* = >99:1. 1.2/1,4 = 99:1) as colourless oil after chromatography on silica gel (100:1 hexane/EtOAc). $^1$H NMR (400 MHz, $CDCl_3$) δ 7.62–7.54 (m, 2H), 7.47–7.31 (m, 5H), 7.21–7.10 (m, 2H), 6.33 (dd, *J* = 15.9, 7.6 Hz, 1H), 6.20 (d, *J* = 16.0 Hz, 1H), 4.39 – 4.22 (m, 2H), 2.41 – 2.17 (m, 1H), 1.31 (d, *J* = 7.2 Hz, 3H). {$^1$H}$^{13}$C NMR (101 MHz, $CDCl_3$) δ 137.1, 135.8, 134.1, 131.7, 131.1, 130.0, 128.1, 127.4, 126.2, 120.3, 23.2, 15.1. HR-MS (APCI$^+$) *m/z* calcd for $C_{16}H_{18}BrSi$, [M+H$^+$]: 317.0361, Found: 317.0357.

The GC trace for the crude mixture of the reaction to make **1f**.

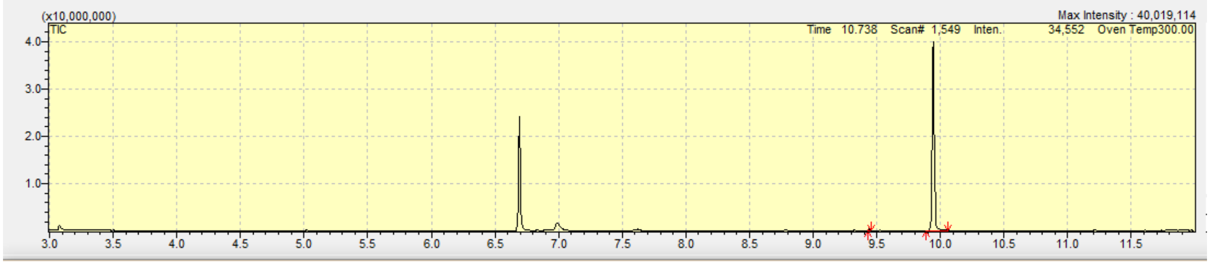


Qualitative Table

Print Edit View Similarity Search

| Peak# | Ret.Time | Start Tm | End Tm | m/z | Area | Area% | Height | Height% | A/H | Mark | Name |
|---|---|---|---|---|---|---|---|---|---|---|---|
| 1 | 9.442 | 9.430 | 9.460 | TIC | 340806 | 0.69 | 339798 | 0.84 | 1.00 | MI | |
| 2 | 9.943 | 9.885 | 10.060 | TIC | 49099775 | 99.31 | 39890559 | 99.16 | 1.23 | MI | |

**(*E*)-(4-(4-methoxyphenyl)but-3-en-2-yl)(phenyl)silane (1o)**

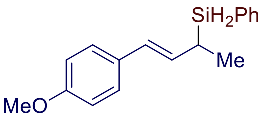


The reaction was stirred for 6 h at 24 ˚C. The title compound was isolated (97.6 mg, 91%, *E/Z* = >99:1. 1.2/1,4 = 95:5) as colourless oil after chromatography on silica gel (100:1 hexane/EtOAc). $^1$H NMR (400 MHz, $CDCl_3$) δ 7.62–7.51 (m, 2H), 7.45–7.31 (m, 3H), 7.26–7.22 (m, 2H), 6.85–6.79 (m, 2H), 6.23 (d, *J* = 16.0 Hz, 1H), 6.16 (dd, *J* = 15.9, 6.9 Hz, 1H), 4.35–4.19 (m, 2H), 3.80 (s, 3H), 2.28–2.19 (m, 1H), 1.28 (d, *J* = 7.2 Hz, 3H). {$^1$H}$^{13}$C NMR (101 MHz, $CDCl_3$) δ 158.7, 135.9, 131.5, 131.1, 131.0, 129.9, 128.1, 127.0, 126.8, 114.1, 55.5, 22.8, 15.4. HR-MS (APCI$^+$) *m/z* calcd for $C_{17}H_{21}OSi$, [M+H$^+$]: 269.1362, Found: 269.1354.

The GC trace for the crude mixture of the reaction to make **1o**.

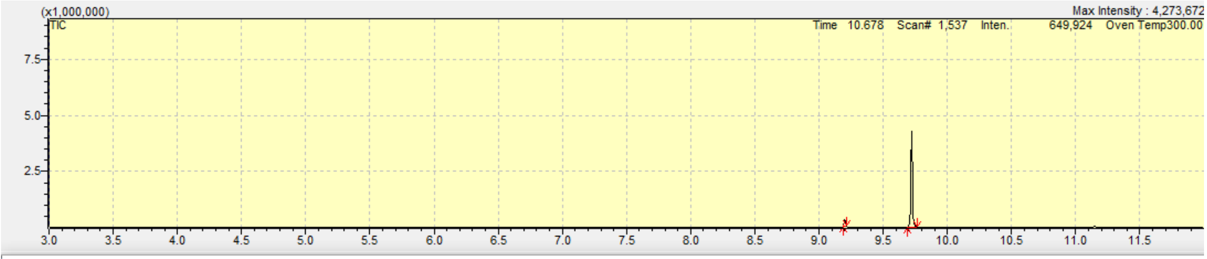


Qualitative Table

Print Edit View Similarity Search

| Peak# | Ret.Time | Start Tm | End Tm | m/z | Area | Area% | Height | Height% | A/H | Mark | Name |
|---|---|---|---|---|---|---|---|---|---|---|---|
| 1 | 9.202 | 9.190 | 9.215 | TIC | 237343 | 5.12 | 274429 | 6.06 | 0.86 | MI | |
| 2 | 9.721 | 9.690 | 9.765 | TIC | 4398767 | 94.88 | 4251134 | 93.94 | 1.03 | MI | |

**(*E*)-(4-(4-(tert-butyl)phenyl)but-3-en-2-yl)(phenyl)silane (1p)**

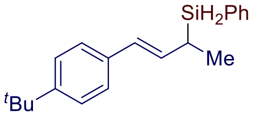


The reaction was stirred for 24 h at 5 ˚C. The title compound was isolated (98.1 mg, 83%, *E*/*Z* = >99:1, 1,2/1.4 = 95:5) as colourless oil after chromatography on silica gel (200:1 hexane/EtOAc). $^1$H NMR (400 MHz, $CDCl_3$) δ 7.51–7.45 (m, 2H), 7.34–7.27 (m, 2H), 7.25–7.13 (m, 5H), 6.21–6.17 (m, 2H), 4.26–4.15 (m, 2H), 2.22–2.10 (m, 1H), 1.25–1.18 (m, 12H). {$^1$H}$^{13}$C NMR (101 MHz, $CDCl_3$) δ 149.8, 135.9, 135.4, 132.4, 131.4, 129.9, 128.1, 127.2, 125.6, 125.5, 34.1, 31.5, 22.9, 15.3. HR-MS (APCI$^+$) *m/z* calcd for $C_{20}H_{26}Si$, [M+H$^+$]: 295.5139, Found: 295.1875.

The GC trace for the crude mixture of the reaction to make **1p**.

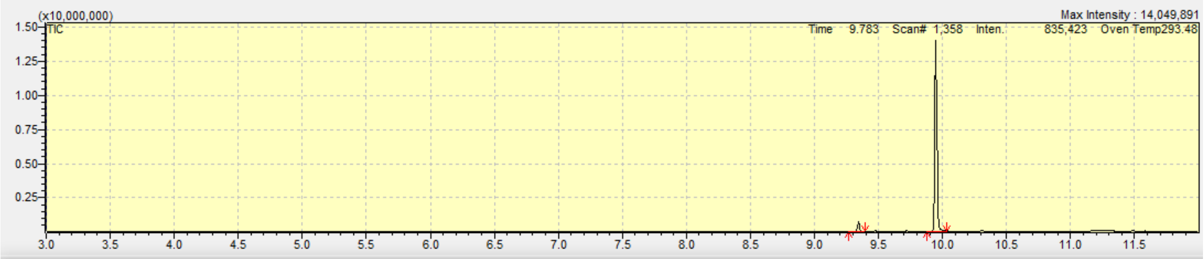


Qualitative Table

Print Edit View Similarity Search

| Peak# | Ret.Time | Start Tm | End Tm | m/z | Area | Area% | Height | Height% | A/H | Mark | Name |
|---|---|---|---|---|---|---|---|---|---|---|---|
| 1 | 9.344 | 9.270 | 9.395 | TIC | 842600 | 4.79 | 722508 | 4.91 | 1.17 | MI | |
| 2 | 9.947 | 9.880 | 10.030 | TIC | 16734523 | 95.21 | 14001945 | 95.09 | 1.20 | MI | |

**(*E*)-(4-(4-(methylthio)phenyl)but-3-en-2-yl)(phenyl)silane (1q)**

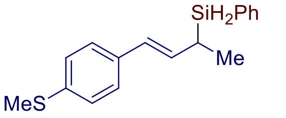


The reaction was stirred for 24 h at 5 ˚C. The title compound was isolated (76.7 mg, 67%, *E*/*Z* = >99:1, 1,2/1.4 = 94:6) as colourless oil after chromatography on silica gel (100:1 hexane/EtOAc). $^1$H NMR (400 MHz, $CDCl_3$) δ 7.62–7.55 (m, 2H), 7.47–7.34 (m, 3H), 7.28–7.17 (m, 4H), 6.31 (dd, *J* = 15.9, 7.1 Hz, 1H), 6.23 (d, *J* = 16.0 Hz, 1H), 4.35–4.27 (m, 2H), 2.49 (s, 3H), 2.32–2.22 (m, 1H), 1.31 (d, *J* = 7.2 Hz, 3H). {$^1$H}$^{13}$C NMR (101 MHz, $CDCl_3$) δ 136.5, 135.8, 135.4, 132.8, 131.3, 130.0, 128.1, 127.2, 126.8, 126.3, 23.0, 16.3, 15.2. GC-MS (EI) *m/z*: calcd for $C_{17}H_{20}OSSi$ [M]$^+$: 284.11; Found: 284.15.

The GC trace for the crude mixture of the reaction to make **1q**.

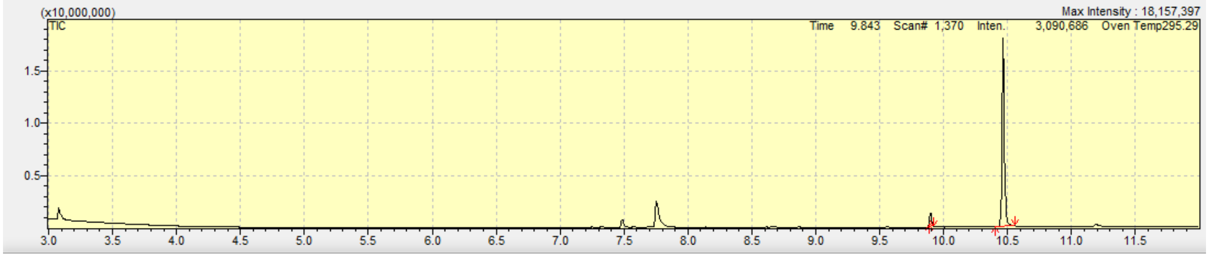


Qualitative Table

Print Edit View Similarity Search

| Peak# | Ret.Time | Start Tm | End Tm | m/z | Area | Area% | Height | Height% | A/H | Mark | Name |
|---|---|---|---|---|---|---|---|---|---|---|---|
| 1 | 9.898 | 9.885 | 9.920 | TIC | 1060878 | 4.22 | 1103987 | 5.78 | 0.96 | MI | |
| 2 | 10.464 | 10.405 | 10.560 | TIC | 24100792 | 95.78 | 18001022 | 94.22 | 1.34 | MI | |

**(*E*)-phenyl(4-(4-(trifluoromethoxy)phenyl)but-3-en-2-yl)silane (1r)**

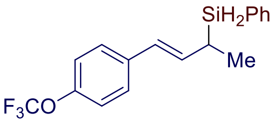


The reaction was stirred for 24 h at 5 ˚C. The title compound was isolated (87.7 mg, 68%, *E*/*Z* = >99:1, 1,2/1.4 = 94:6) as colourless oil after chromatography on silica gel (100:0 hexane/EtOAc). $^{1}$H NMR (400 MHz, $CDCl_3$) δ 7.58 (dd, *J* = 7.8, 1.4 Hz, 2H), 7.44–7.27 (m, 5H), 7.16–7.07 (m, 2H), 6.31 (dd, *J* = 15.9, 7.1 Hz, 1H), 6.24 (d, *J* = 16.0 Hz, 1H), 4.30 (d, *J* = 2.9 Hz, 2H), 2.33–2.18 (m, 1H), 1.30 (d, *J* = 7.2 Hz, 3H). {$^{1}$H}$^{13}$C NMR (101 MHz, $CDCl_3$) δ 148.0, 137.0, 135.8, 134.4, 131.1, 130.1, 128.2, 127.0, 126.0, 121.2, 120.7 (q, $J_{C\text{-}F}$ = 256.7 Hz), 23.2, 15.2. $^{19}$F NMR (377 MHz, $CDCl_3$) δ -57.88. GC-MS (EI) *m/z*: calcd for $C_{17}H_{17}F_3OSi$ $[M]^+$: 322.10; Found: 322.10.

The GC trace for the crude mixture of the reaction to make **1r**.

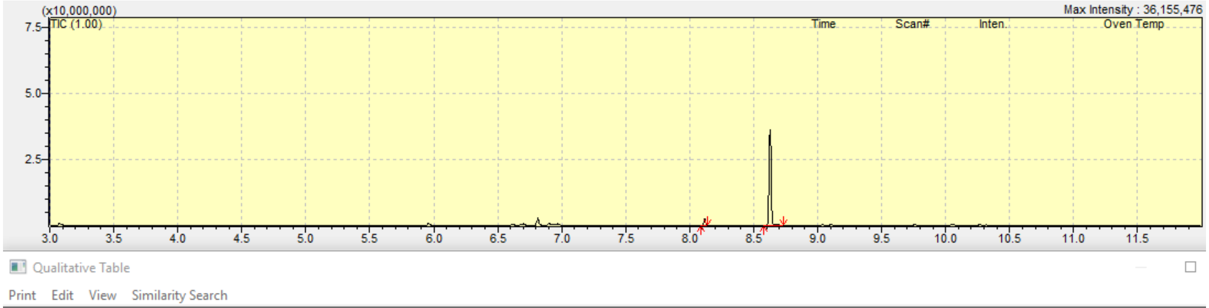


| Peak# | Ret.Time | Start Tm | End Tm | m/z | Area | Area% | Height | Height% | A/H | Mark | Name |
|---|---|---|---|---|---|---|---|---|---|---|---|
| 1 | 8.118 | 8.085 | 8.140 | TIC | 2536237 | 5.99 | 2403524 | 6.24 | 1.06 | MI | |
| 2 | 8.629 | 8.580 | 8.730 | TIC | 39771736 | 94.01 | 36103419 | 93.76 | 1.10 | MI | |

**(*E*)-(4-(4-fluorophenyl)but-3-en-2-yl)(phenyl)silane (1s)**

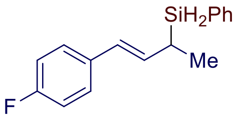


The reaction was stirred for 24 h at 5 ˚C. The title compound was isolated (76.7 mg, 75%, *E*/*Z* = >99:1, 1,2/1.4 = 92:8) as colourless oil after chromatography on silica gel (50:1 hexane/EtOAc). $^{1}$H NMR (400 MHz, $CDCl_3$) δ 7.64–7.56 (m, 2H), 7.44–7.34 (m, 3H), 7.29–7.23 (m, 2H), 7.03–6.95 (m, 2H), 6.25 (m, 2H), 4.41–4.27 (m, 2H), 2.34–2.20 (m, 1H), 1.31 (d, *J* = 7.2 Hz, 3H). {$^{1}$H}$^{13}$C NMR (101 MHz, $CDCl_3$) δ 161.9 (d, $J_{C\text{-}F}$ = 245.4 Hz), 135.8, 134.4 (d, $J_{C\text{-}F}$ = 3.3 Hz), 132.9 (d, $J_{C\text{-}F}$ = 2.2 Hz), 131.2, 130.0, 128.1, 127.3 (d, $J_{C\text{-}F}$ = 7.8 Hz), 126.3, 115.4 (d, $J_{C\text{-}F}$ = 21.5 Hz), 23.0, 15.3. $^{19}$F NMR (377 MHz, $CDCl_3$) δ -116.01. HR-MS (APCI $^+$) *m/z* calcd for $C_{16}H_{18}FSi$, $[M+H^+]$: 257.3980, Found: 257.1162.

The GC trace for the crude mixture of the reaction to make **1s**.

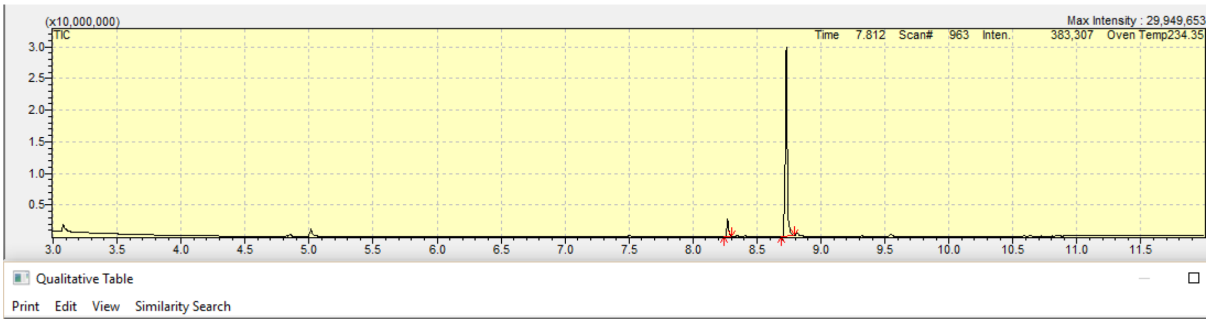


| Peak# | Ret.Time | Start Tm | End Tm | m/z | Area | Area% | Height | Height% | A/H | Mark | Name |
|---|---|---|---|---|---|---|---|---|---|---|---|
| 1 | 8.271 | 8.245 | 8.300 | TIC | 2903994 | 7.61 | 2725630 | 8.37 | 1.07 | MI | |
| 2 | 8.728 | 8.690 | 8.795 | TIC | 35240290 | 92.39 | 29833005 | 91.63 | 1.18 | MI | |

**(*E*)-(4-(4-chlorophenyl)but-3-en-2-yl)(phenyl)silane (1t)**

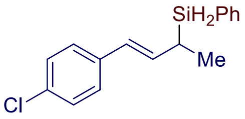


The reaction was stirred for 24 h at 5 ˚C. The title compound was isolated (71.5 mg, 66%, *E*/*Z* = >99:1, 1,2/1.4 = 95:5) as colourless oil after chromatography on silica gel (100:0 hexane/EtOAc). $^{1}$H NMR (400 MHz, $CDCl_3$) δ 7.62 (dd, *J* = 7.8, 1.4 Hz, 2H), 7.49–7.39 (m, 3H), 7.31–7.22 (m, 4H), 6.35 (dd, *J* = 15.9, 7.4 Hz, 1H), 6.25 (d, *J* = 16.1 Hz, 1H), 4.35 (d, *J* = 2.7 Hz, 2H), 2.36 – 2.24 (m, 1H), 1.34 (d, *J* = 7.2 Hz, 3H). {$^{1}$H}$^{13}$C NMR (101 MHz, $CDCl_3$) δ 136.7, 135.8, 134.0, 132.2, 131.1, 130.0, 128.7, 128.1, 127.1, 126.2, 23.1, 15.2. HR-MS (APCI$^+$) *m/z* calcd for $C_{16}H_{18}ClSi$, $[M+H^+]$: 273.8526, Found: 273.0860.

The GC trace for the crude mixture of the reaction to make **1t**.

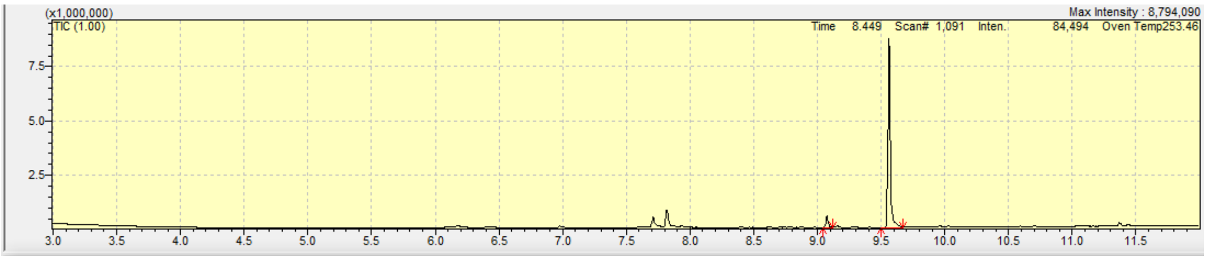


Qualitative Table

Print Edit View Similarity Search

| Peak# | Ret.Time | Start Tm | End Tm | m/z | Area | Area% | Height | Height% | A/H | Mark | Name |
|---|---|---|---|---|---|---|---|---|---|---|---|
| 1 | 9.075 | 9.045 | 9.120 | TIC | 664422 | 5.32 | 544480 | 5.89 | 1.22 | MI | |
| 2 | 9.561 | 9.500 | 9.670 | TIC | 11828229 | 94.68 | 8693870 | 94.11 | 1.36 | MI | |

**(*E*)-4-(3-(phenylsilyl)but-1-en-1-yl)phenyl trifluoromethanesulfonate (1u)**

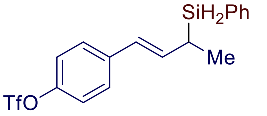


The reaction was stirred for 6 h at 24 ˚C. The title compound was isolated (140.8 mg, 91%, *E*/*Z* = >99:1, 1,2/1,4(and minor isomer) = 80:20) as colourless oil after chromatography on silica gel (10:1 hexane/EtOAc). $^{1}$H NMR (400 MHz, $CDCl_3$) δ 7.57 (dd, *J* = 7.8, 1.4 Hz, 2H), 7.43–7.33 (m, 4H), 7.20–7.12 (m, 3H), 6.35 (dd, *J* = 15.9, 7.5 Hz, 1H), 6.24 (d, *J* = 16.0 Hz, 1H), 4.30 (d, *J* = 2.9 Hz, 2H), 2.35 – 2.22 (m, 1H), 1.30 (d, *J* = 7.1 Hz, 3H). {$^{1}$H}$^{13}$C NMR (101 MHz, $CDCl_3$) δ 148.2, 138.6, 135.8, 130.9, 130.1, 129.2, 128.2, 127.3, 125.5, 122.1 (q, $J_{C\text{-}F}$ = 311.7 Hz), 121.5, 23.3, 15.1. $^{19}$F NMR (377 MHz, $CDCl_3$) δ -72.83. HR-MS (APCI$^{+}$) *m*/*z* calcd for $C_{17}H_{18}F_3O_3SSi$, [M+H$^{+}$]: 387.0698, Found: 387.0698.

**(*E*)-4-(1-(phenylsilyl)but-2-en-1-yl)phenyl trifluoromethanesulfonate (2u)**

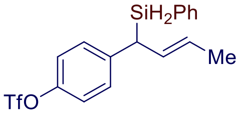


The title minor compound was isolated via chromatography on silica gel (10:1 hexane/EtOAc) together with the major 1,2-hydrosilylation product. $^{1}$H NMR (400 MHz, $CDCl_3$) δ 5.77 (ddq, *J* = 15.0, 8.6, 1.5 Hz, 1H), 5.58 – 5.48 (m, 1H).

The GC trace for the crude mixture of the reaction to make **1u**.

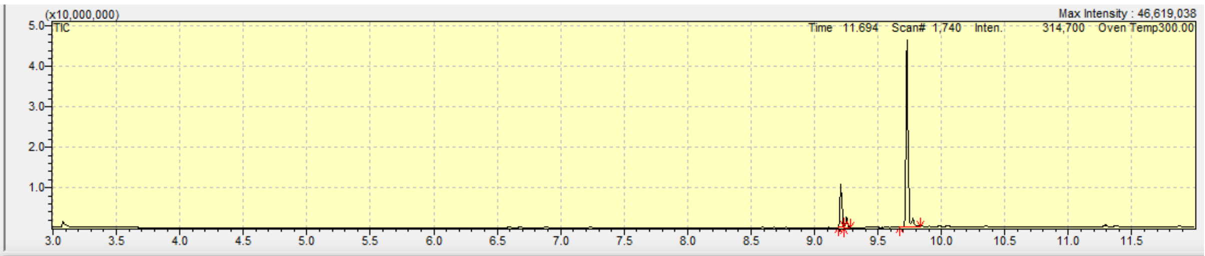


Qualitative Table

Print Edit View Similarity Search

| Peak# | Ret.Time | Start Tm | End Tm | m/z | Area | Area% | Height | Height% | A/H | Mark | Name |
|---|---|---|---|---|---|---|---|---|---|---|---|
| 1 | 9.208 | 9.190 | 9.230 | TIC | 11008410 | 15.53 | 10596435 | 17.82 | 1.04 | MI | |
| 2 | 9.252 | 9.235 | 9.280 | TIC | 2697206 | 3.80 | 2518149 | 4.24 | 1.07 | MI | |
| 3 | 9.729 | 9.670 | 9.835 | TIC | 57182240 | 80.67 | 46335812 | 77.94 | 1.23 | MI | |

**(*E*)-4-(3-(phenylsilyl)but-1-en-1-yl)phenyl acetate (1v)**

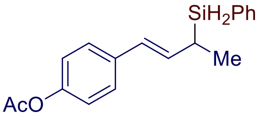


The reaction was stirred for 24 h at 5 ˚C. The title compound was isolated (106 mg, 89%, *E*/*Z* = >99:1, 1,2/1.4 = 95:5) as colourless oil after chromatography on silica gel (25:1 hexane/EtOAc). $^{1}$H NMR (400 MHz, $CDCl_3$) δ 7.57 (dd, *J* = 7.9, 1.5 Hz, 2H), 7.45–7.33 (m, 3H), 7.31–7.27 (m, 2H), 7.00 (d, *J* = 8.7 Hz, 2H), 6.31–6.21 (m, 2H), 4.29 (d, *J* = 2.9 Hz, 2H), 2.32–2.25 (m, 4H), 1.29 (d, *J* = 7.2 Hz, 3H). {$^{1}$H}$^{13}$C NMR (101 MHz, $CDCl_3$) δ 169.7, 149.5, 136.1, 135.8, 133.5, 131.2, 130. 0, 128.1, 126.8, 126.5, 121.7, 23.0, 21.3, 15.2. GC-MS (EI) *m*/*z*: calcd for $C_{18}H_{20}O_2Si$ [M]$^{+}$: 296.12; Found: 296.15.

The GC trace for the crude mixture of the reaction to make **1v**.

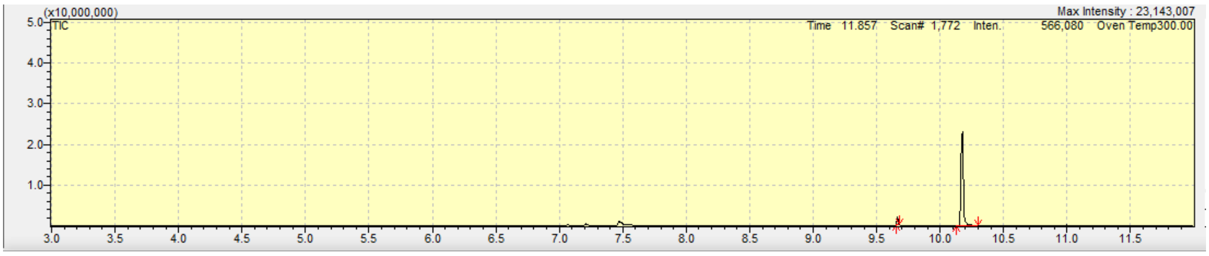


Qualitative Table

Print Edit View Similarity Search

| Peak# | Ret.Time | Start Tm | End Tm | m/z | Area | Area% | Height | Height% | A/H | Mark | Name |
|---|---|---|---|---|---|---|---|---|---|---|---|
| 1 | 9.664 | 9.650 | 9.680 | TIC | 1672307 | 5.48 | 1775098 | 7.15 | 0.94 | MI | |
| 2 | 10.173 | 10.130 | 10.295 | TIC | 28870718 | 94.52 | 23050286 | 92.85 | 1.25 | MI | |

**(*E*)-methyl 4-(3-(phenylsilyl)but-1-en-1-yl)benzoate (1w)**

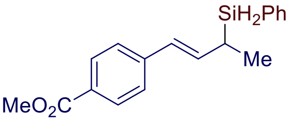


The reaction was stirred for 24 h at 5 ˚C with 0.2 mmol scale. The title compound was isolated (43.0 mg, 73%, *E/Z* = >99:1, 1,2/1.4 = 95:5) as colourless oil after chromatography on silica gel (25:1 hexane/EtOAc). $^1$H NMR (400 MHz, $CDCl_3$) δ 8.00–7.93 (m, 3H), 7.59–7.55 (m, 2H), 7.41–7.34 (m, 3H), 7.28–7.23 (m, 1H), 6.47 (dd, *J* = 15.9, 7.7 Hz, 1H), 6.29 (d, *J* = 16.6 Hz, 1H), 4.31 (dd, *J* = 2.9, 0.8 Hz, 2H), 3.91 (s, 3H), 2.36–2.26 (m, 1H), 1.31 (d, *J* = 7.1 Hz, 3H). {$^1$H}$^{13}$C NMR (101 MHz, $CDCl_3$) δ 167.2, 142.7, 136.4, 135.8, 130.9, 130.0, 129.8, 128.5, 128.2, 126.6, 125.7, 52.1, 23.5, 15.1. GC-MS (EI) *m/z*: calcd for $C_{18}H_{20}O_2Si$ $[M]^+$: 296.12; Found: 296.15.

The GC trace for the crude mixture of the reaction to make **1w**.

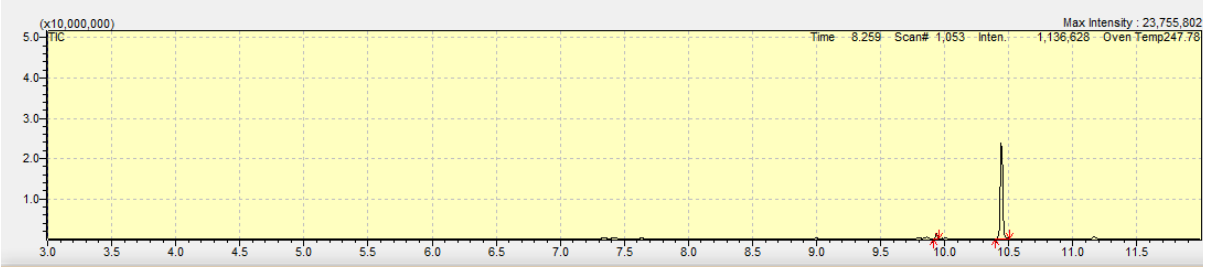


Qualitative Table

Print Edit View Similarity Search

| Peak# | Ret.Time | Start Tm | End Tm | m/z | Area | Area% | Height | Height% | A/H | Mark | Name |
|---|---|---|---|---|---|---|---|---|---|---|---|
| 1 | 9.936 | 9.915 | 9.955 | TIC | 1476599 | 4.61 | 1396243 | 5.59 | 1.06 | MI | |
| 2 | 10.442 | 10.395 | 10.510 | TIC | 30534573 | 95.39 | 23601477 | 94.41 | 1.29 | MI | |

**(*E*)-(4-(4-(1,3-dioxolan-2-yl)phenyl)but-3-en-2-yl)(phenyl)silane (1x)**

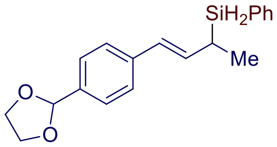


The reaction was stirred for 6 h at 24 ˚C. The title compound was isolated (94.8 mg, 76%, *E/Z* = >99:1, 1,2/1.4 = 93:7) as colourless oil after chromatography on silica gel (10:1 hexane/EtOAc). $^1$H NMR (400 MHz, $CDCl_3$) δ 7.61–7.55 (m, 2H), 7.42–7.32 (m, 7H), 6.37 (dd, *J* = 15.9, 7.3 Hz, 1H), 6.28 (d, *J* = 16.0 Hz, 1H), 5.81 (s, 1H), 4.32 (d, *J* = 2.9 Hz, 2H), 4.16–4.11 (m, 2H), 4.06–4.01 (m, 2H), 2.34–2.24 (m, 1H), 1.31 (d, *J* = 7.2 Hz, 3H). {$^1$H}$^{13}$C NMR (101 MHz, $CDCl_3$) δ 139.1, 136.2, 135.8, 133.9, 131.2, 130.0, 128.1, 127.0, 126.8, 125.8, 103.8, 65.4, 23.1, 15.2. GC-MS (EI) *m/z*: calcd for $C_{19}H_{22}O_2Si$ ($[M]^+$): 310.14; Found: 309.15.

The GC trace for the crude mixture of the reaction to make **1x**.

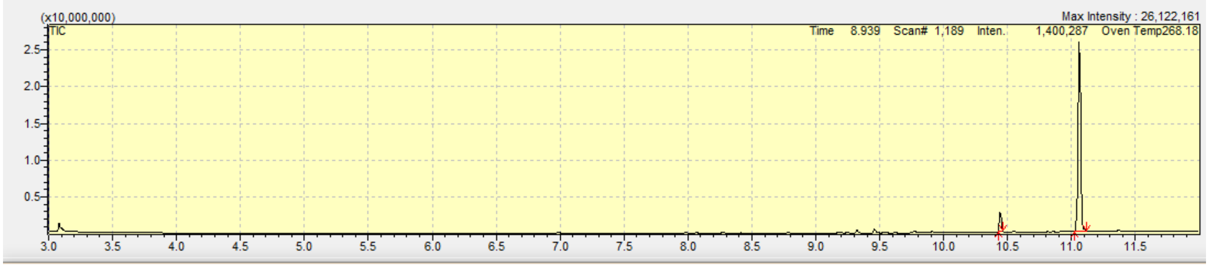


Qualitative Table

Print Edit View Similarity Search

| Peak# | Ret.Time | Start Tm | End Tm | m/z | Area | Area% | Height | Height% | A/H | Mark | Name |
|---|---|---|---|---|---|---|---|---|---|---|---|
| 1 | 10.442 | 10.425 | 10.465 | TIC | 2922499 | 7.45 | 2628635 | 9.25 | 1.11 | MI | |
| 2 | 11.060 | 11.025 | 11.115 | TIC | 36290521 | 92.55 | 25791573 | 90.75 | 1.41 | MI | |

**(*E*)-4-(3-(phenylsilyl)but-1-en-1-yl)aniline (1y)**

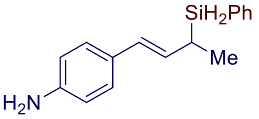


The reaction was stirred for 6 h at 24 ˚C. The title compound was isolated (83.0 mg, 82%, *E*/*Z* = >99:1, 1,2/1.4 = 83:17) as colourless oil after chromatography on silica gel (5:1 hexane/EtOAc). $^{1}$H NMR (400 MHz, $CDCl_3$) δ 7.61–7.56 (m, 2H), 7.42–7.34 (m, 3H), 7.15–7.07 (m, 2H), 6.63–6.60 (m, 2H), 6.19 (d, *J* = 16.0 Hz, 1H), 6.11 (dd, *J* = 15.9, 7.3 Hz, 1H), 4.32–4.22 (m, 2H), 3.61 (s, 2H), 2.26–2.17 (m, 1H), 1.27 (d, *J* = 7.1 Hz, 3H). {$^{1}$H}$^{13}$C NMR (101 MHz, $CDCl_3$) δ 145.3, 135.9, 131.6, 129.9, 129.5, 128.7, 128.1, 127.2, 127.0, 115.3, 22.7, 15.4. GC-MS (EI) *m*/*z*: calcd for $C_{16}H_{19}NSi$ $[M]^+$: 253.41; Found: 253.15.

The GC trace for the crude mixture of the reaction to make **1y**.

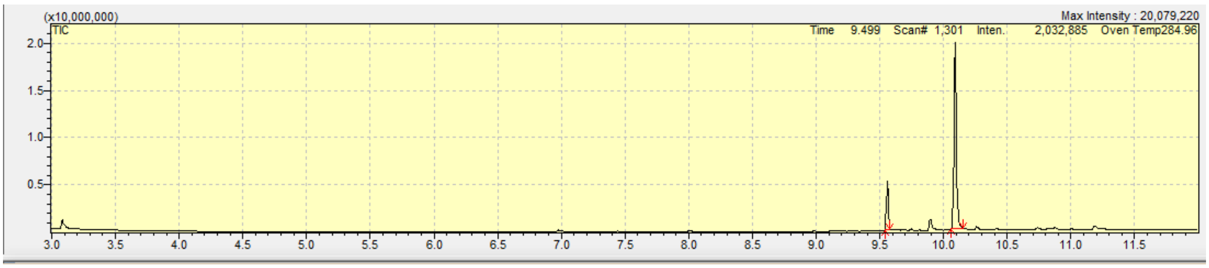


Qualitative Table

Print Edit View Similarity Search

| Peak# | Ret.Time | Start Tm | End Tm | m/z | Area | Area% | Height | Height% | A/H | Mark | Name |
|---|---|---|---|---|---|---|---|---|---|---|---|
| 1 | 9.559 | 9.540 | 9.580 | TIC | 5685967 | 17.29 | 5099578 | 20.59 | 1.11 | MI | |
| 2 | 10.090 | 10.060 | 10.150 | TIC | 27198596 | 82.71 | 19669292 | 79.41 | 1.38 | MI | |

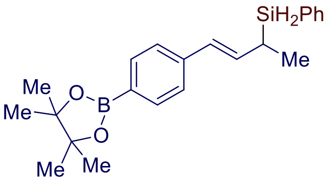


**(*E*)-phenyl(4-(4-(4,4,5,5-tetramethyl-1,3,2-dioxaborolan-2-yl)phenyl)but-3-en-2-yl)silane (1z)**

The reaction was stirred for 6 h at 24 ˚C. The title compound was isolated (127 mg, 87%, *E*/*Z* = >99:1, 1,2/1.4 = 95:5) as colourless oil after chromatography on silica gel (30:1 hexane/EtOAc). $^{1}$H NMR (400 MHz, $CDCl_3$) δ 7.75 (d, *J* = 8.1 Hz, 2H), 7.58 (dd, *J* = 7.9, 1.5 Hz, 2H), 7.45–7.29 (m, 5H), 6.42 (dd, *J* = 15.9, 7.6 Hz, 1H), 6.28 (d, *J* = 16.0 Hz, 1H), 4.32 (d, *J* = 2.8 Hz, 2H), 2.34–2.23 (m, 1H), 1.36 (s, 12H), 1.31 (d, *J* = 7.2 Hz, 3H). {$^{1}$H}$^{13}$C NMR (75 MHz, $CDCl_3$) δ 140.9, 135.8, 135.2, 134.4, 131.1, 130.0, 128.1, 127.5, 125.2, 83.8, 25.0, 23.2, 15.1. The carbon connected with boron could not be observed due to quadrupole of boron. GC-MS (EI) *m*/*z*: calcd for $C_{22}H_{29}BO_2Si$ ($[M]^+$): 364.20; Found: 364.25.

The GC trace for the crude mixture of the reaction to make **1z**.

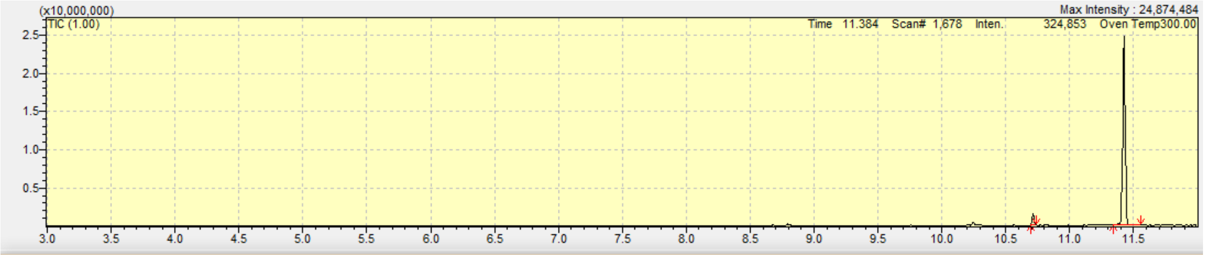


Qualitative Table

Print Edit View Similarity Search

| Peak# | Ret.Time | Start Tm | End Tm | m/z | Area | Area% | Height | Height% | A/H | Mark | Name |
|---|---|---|---|---|---|---|---|---|---|---|---|
| 1 | 10.714 | 10.695 | 10.735 | TIC | 1700120 | 4.94 | 1542682 | 5.87 | 1.10 | MI | |
| 2 | 11.425 | 11.345 | 11.560 | TIC | 32712704 | 95.06 | 24751194 | 94.13 | 1.32 | MI | |

**(*E*)-phenyl(1-phenylhex-1-en-3-yl)silane (1aa)**

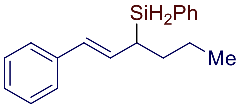


The reaction was stirred for 48 h at 24 °C. The title compound was isolated (61.0 mg, 57%, *E/Z* = >99:1, 1,2/1.4 = 94:6) as colourless oil after chromatography on silica gel (100:0 hexane/EtOAc). $^1$H NMR (400 MHz, $CDCl_3$) δ 7.54 (dd, *J* = 7.9, 1.4 Hz, 2H), 7.38–7.31 (m, 3H), 7.25 (d, *J* = 3.3 Hz, 4H), 7.17–7.11 (m, 1H), 6.24 (d, *J* = 15.9 Hz, 1H), 6.11 (dd, *J* = 15.8, 9.2 Hz, 1H), 4.31–4.26 (m, 2H), 2.21–2.13 (m, 1H), 1.62–1.54 (m, 2H), 1.49–1.39 (m, 1H), 1.37–1.27 (m, 1H), 0.86 (t, *J* = 7.3 Hz, 3H). {$^1$H}$^{13}$C NMR (101 MHz, $CDCl_3$) δ 138.2, 135.9, 132.1, 131.4, 129.9, 128.9, 128.6, 128.1, 126.7, 125.9, 32.9, 29.5, 22.6, 14.0. GC-MS (EI) *m/z*: calcd for $C_{18}H_{22}Si$ $[M]^+$: 266.45; Found: 266.20.

The GC trace for the crude mixture of the reaction to make **1aa**.

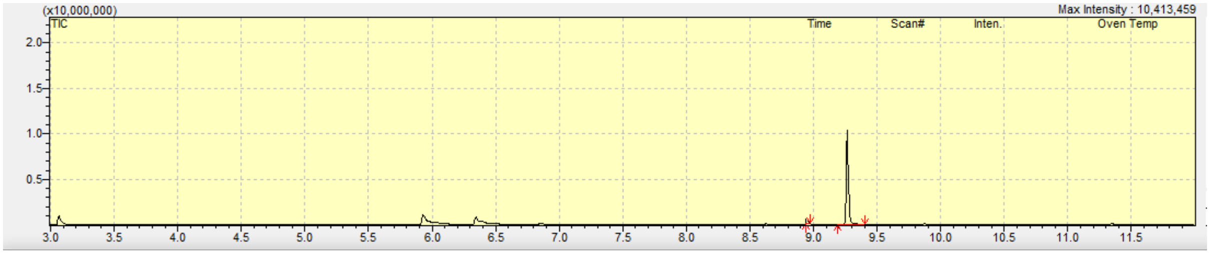


Qualitative Table

Print Edit View Similarity Search

| Peak# | Ret.Time | Start Tm | End Tm | m/z | Area | Area% | Height | Height% | A/H | Mark | Name |
|---|---|---|---|---|---|---|---|---|---|---|---|
| 1 | 8.949 | 8.935 | 8.970 | TIC | 726190 | 5.53 | 659303 | 5.99 | 1.10 | MI | |
| 2 | 9.265 | 9.190 | 9.405 | TIC | 12408588 | 94.47 | 10344330 | 94.01 | 1.20 | MI | |

**General procedure for Separation of *cis*-1,3-dienes via hydrosilylation reaction**

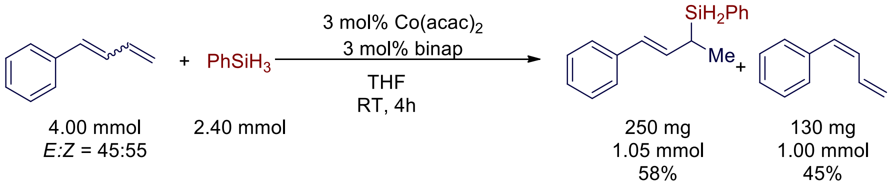


In an Ar-filled glovebox, a mixture of $Co(acac)_2$ (19.0 mg, 0.120 mmol) and binap (45.0 mg, 0.120 mmol) in THF (5 mL) was added into a 20-mL screw-capped vial containing a magnetic stirring bar. The resulting mixture was stirred for 2 mins prior adding phenylsilane (0.260 g, 2.40 mmol) and *trans*/*cis*-1,3-dienes (0.520 g, 4.00 mmol) successively. The vial was removed from the glove box, and the mixture was stirred at room temperature for 6 hours. After that, a GC-MS analysis was done and the crude reaction mixture was concentrated under vacuum using a 30 ºC water bath and the residue was purified by flash column chromatography using hexane and ethyl acetate (100:1) as eluents yielding (*E*)-allylsilane **1a** (0.250 g, 1.05 mmol, 58%) as a colorless oil and recovering (*Z*)-1-phenyl-1,3-diene (Z/E = 98:2, 0.130 g, 1.0 mmol, 45%).

The GC trace for the crude reaction mixture to form **1a** ($t_R$ = 5.0 min) and **(Z)-1-phenyl-1,3-diene** ($t_R$ = 8.8 min)**.**

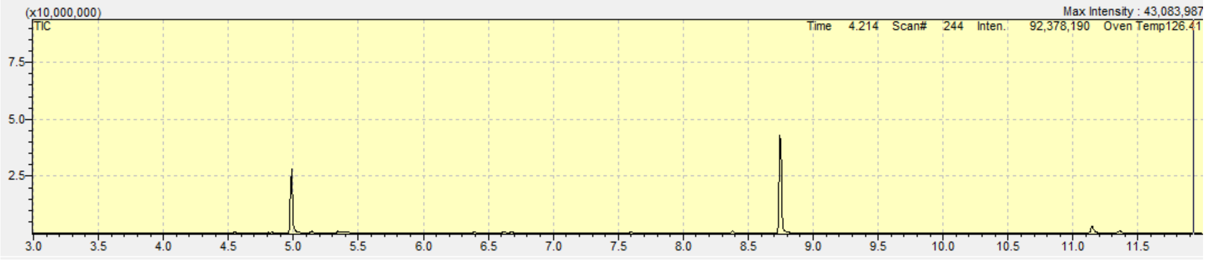


**(*Z*)-1-phenyl-1,3-diene**

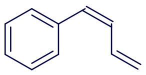

$^1$H NMR (400 MHz, $CDCl_3$) δ 7.36–7.31 (m, 4H), 7.28–7.22 (m, 1H), 6.89 (dddd, *J* = 16.9, 11.2, 10.1, 1.0 Hz, 1H), 6.47 (d, *J* = 11.5 Hz, 1H), 6.27 (t, *J* = 11.3 Hz, 1H), 5.38 (ddd, *J* = 16.9, 1.8, 0.9 Hz, 1H), 5.23 (dt, *J* = 10.1, 2.1 Hz, 1H). {$^1$H}$^{13}$C NMR (101 MHz, $CDCl_3$) δ 137.5, 133.4, 130.9, 130.6, 129.2, 128.4, 127.2, 119.8. GC-MS (EI) *m*/*z*: calcd for $C_{10}H_{10}$ $[M]^+$: 130.08; Found: 129.15.

**General procedure for Co-catalyzed Markovnikov 1,2-hydrosilylation of trans/cis 1,3-dienes (Outside glovebox under nitrogen protection)**

$Co(acac)_2$ (1.0 mg, 4.0 *μ*mol) and xantphos (2.3 mg, 4.0 *μ*mol) were pre-weighted in air on the open bench and added into a 25 mL Schlenk flask. The Schlenk flask was back-filled with $N_2$ thrice and dry THF (1 mL) was then added. The resulting mixture was stirred for 2 mins to give a pale pink solution. Subsequently, phenylsilane (54.1 mg, 0.500 mmol) and 1-phenylbutadiene (51.6 mg, 0.400 mmol) were added with the aid of a microsyringe which gave a pale-yellow solution. Upon stirring the mixture for 6 hours, a GC-MS analysis was conducted to determine the selectivity of the crude reaction mixture prior concentrating it under vacuum. Subsequently, the residue was purified by flash column chromatography using a mixture of ethyl acetate and hexane as eluent yielding (*E*)-phenyl(4-phenylbut-3-en-2-yl)silane **1a** (74.7mg, 78%, *E*/*Z* = >99:1, 1,2/1.4 = 94:6) as a colorless oil.

The GC trace for the crude mixture of the reaction to form **1a**.

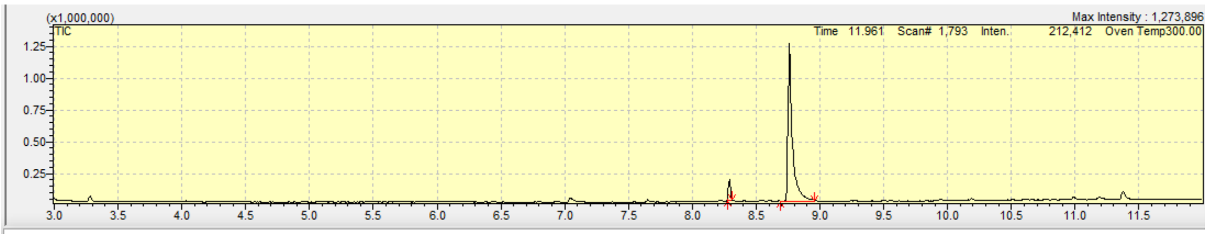


Qualitative Table

Print Edit View Similarity Search

| Peak# | Ret.Time | Start Tm | End Tm | m/z | Area | Area% | Height | Height% | A/H | Mark | Name |
|---|---|---|---|---|---|---|---|---|---|---|---|
| 1 | 8.290 | 8.275 | 8.315 | TIC | 179904 | 5.76 | 165017 | 11.74 | 1.09 | MI | |
| 2 | 8.760 | 8.690 | 8.955 | TIC | 2943472 | 94.24 | 1240123 | 88.26 | 2.37 | MI | |

**General procedure for gram scale Co-catalyzed Markovnikov 1,2-hydrosilylation of 1,3-dienes**

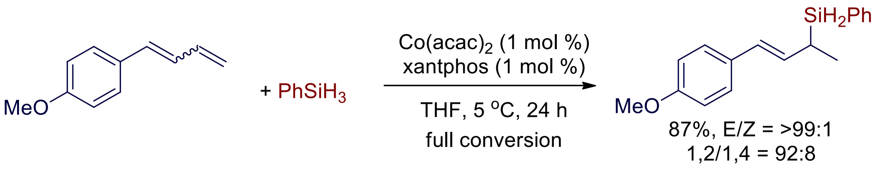


$Co(acac)_2$ (25.7 mg, 0.100 mmol) and xantphos (57.9 mg, 0.100 mmol) were weighted in air on the open bench and added into a 100 mL Schlenk flask. The Schlenk flask was back-filled with $N_2$ thrice and dry THF (25 mL) was then added. The resulting mixture was stirred for 2 mins to give a pale pink solution prior adding phenylsilane (1.35 g, 12.5 mmol) and 1-(buta-1,3-dien-1-yl)-4-methoxybenzene (1.60 g, 10.0 mmol) successively. The Schlenk flask was removed from the glove box, and the mixture was stirred at room

temperature for 6 hours. Subsequently, a GC-MS analysis was conducted to determine the selectivity of the crude reaction mixture prior concentrating it under vacuum. The residue was then purified by flash column chromatography using a mixture of ethyl acetate and hexane as eluent yielding (*E*)-(4-(4-methoxyphenyl)but-3-en-2-yl)(phenyl)silane **1o** (2.34 g, 87 %, *E*/*Z* = >99:1, 1,2/1.4 = 92:8) as a colorless oil.

The GC trace for the crude reaction mixture to form **1o.**

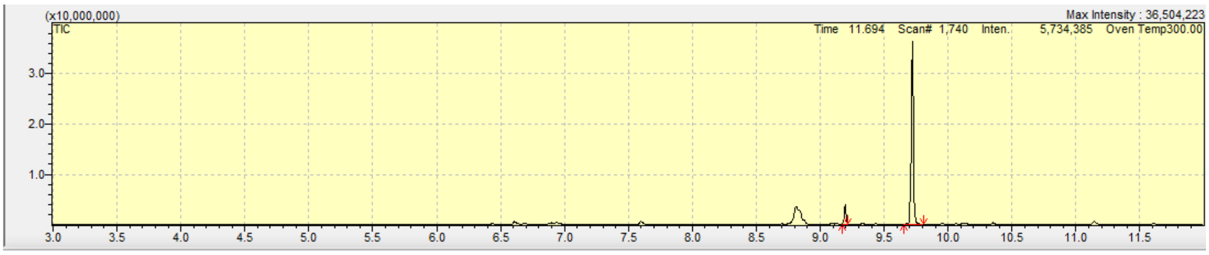


Qualitative Table

Print Edit View Similarity Search

| Peak# | Ret.Time | Start Tm | End Tm | m/z | Area | Area% | Height | Height% | A/H | Mark | Name |
|---|---|---|---|---|---|---|---|---|---|---|---|
| 1 | 9.195 | 9.175 | 9.215 | TIC | 3903588 | 7.75 | 3820825 | 9.49 | 1.02 | MI | |
| 2 | 9.721 | 9.655 | 9.810 | TIC | 46447584 | 92.25 | 36430526 | 90.51 | 1.27 | MI | |

**General procedure for asymmetric Co-catalyzed Markovnikov 1,2-hydrosilylation of *trans*-1,3-dienes**

In an Ar-filled glovebox, a mixture of Co(acac)$_2$ (5.1 mg, 20 $\mu$mol) and (*R*)-difluorphos (14.0 mg, 20 $\mu$mol) in THF (1 mL) was added into a 4-mL screw-capped vial containing a magnetic stirring bar. The resulting mixture was stirred for 2 mins prior adding phenylsilane (54.1 mg, 0.500 mmol) and *trans*-1,3-dienes (0.400 mmol) successively. The vial was removed from the glove box, and the mixture was stirred at room temperature for 6 hours. After that, the crude reaction mixture was concentrated under vacuum and the residue was purified by flash column chromatography using a mixture of ethyl acetate and hexane as eluent. The details and characterization data of the products are stated below. The enantiopurity of the product was analyzed by chiral HPLC or oxidized to (*E*)-allylic alcohol prior analysing with chiral HPLC.

**Procedure for oxidation of (*E*)-allylsilanes**

A mixture of (*E*)-allylsilane (0.200 mmol) and KHCO$_3$ (0.200 mmol) was added into a mixture of THF (0.5 mL) and methanol (0.5 mL) in a 4-mL screw-capped vial containing a magnetic stirring bar. The resulting mixture was stirred for 2 mins. Subsequently, 30% aqueous H$_2$O$_2$ (0.500 mmol) was dropwise slowly into the reaction mixture and then stirred at room temperature for 5 hours. Anhydrous sodium thiosulfate was then added to quenched the excess oxidant prior it was extracted with ethyl acetate, dried over sodium sulfate, filtered and concentrated under vacuum. The residue was then purified by flash column chromatography using hexane and ethyl acetate as eluents yielding (*E*)-allylic alcohol.

**(*S*,*E*)-phenyl(4-phenylbut-3-en-2-yl)silane (1a)**

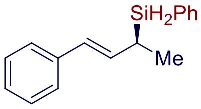


The reaction was stirred for 6 h at 24 ˚C. The title compound was isolated (68.0 mg, 71%, *E*/*Z* = >99:1) as colourless oil after chromatography on silica gel (100:1 hexane/EtOAc). $^1$H NMR (400 MHz, CDCl$_3$) δ 7.63–7.58 (m, 2H), 7.47–7.36 (m, 3H), 7.34–7.28 (m, 4H), 7.23–7.17 (m, 1H), 6.35 (dd, *J* = 15.9, 6.8 Hz, 1H), 6.29 (d, *J* = 16.0 Hz, 1H), 4.35–4.28 (m, 2H), 2.35–2.23 (m, 1H), 1.32 (d, *J* = 7.2 Hz, 3H). {$^1$H}$^{13}$C NMR (101 MHz, CDCl$_3$) δ 138.2, 135.9, 133.2, 131.30, 123.0, 128.6, 128.1, 127.4, 126.7, 125.9, 23.0, 15.3. HR-MS (APCI$^+$) *m/z* calcd for C$_{16}$H$_{18}$Si, [M+H$^+$]: 239.1256, Found: 239.1258. Optical Rotation: $[\alpha]^{20}_D$ = -15.00 (c = 0.20 g/cm$^3$, CHCl$_3$). The absolute configuration was assigned by oxidizing it to the corresponding allylic alcohol (**1a’’’**).

**(*S,E*)-4-phenylbut-3-en-2-ol (1a''')**

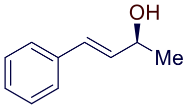

The title compound was isolated (34.5 mg, 82%, 88:11 er) as colourless oil after chromatography on silica gel (10:1 hexane/EtOAc). $^1$H NMR (400 MHz, $CDCl_3$) δ 7.39 (dd, *J* = 5.3, 3.4 Hz, 2H), 7.34–7.29 (m, 2H), 7.26–7.21 (m, 1H), 6.57 (d, *J* = 16.0 Hz, 1H), 6.27 (dd, *J* = 15.9, 6.4 Hz, 1H), 4.55–4.44 (m, 1H), 1.38 (d, *J* = 6.4 Hz, 3H).{$^1$H} $^{13}$C NMR (101 MHz, $CDCl_3$) δ 136.9, 133.7, 129.6, 128.7, 127.8, 126.6, 69.1, 23.6. Optical Rotation: $[\alpha]^{20}_D$ = -16.10 (c = 0.20 g/cm$^3$, $CHCl_3$). The absolute configuration was assigned by comparing with the optical rotation reported in the literature.[5] HPLC condition: Chiral column IB, n-hexane/i-PrOH = 90:10, flow rate = 0.35 mL/min, wavelength = 254 nm, $t_R$ = 19.8 min for minor isomer, $t_R$ = 25.3 min for major isomer.

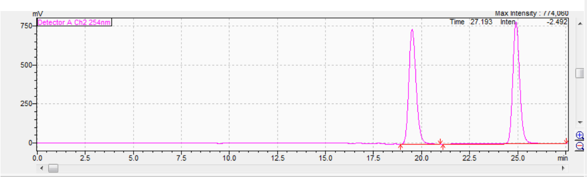

Results View - Peak Table

| Peak# | Ret. Time | Area | Height | Mark | Conc. | Unit | ID# | Name | Area% |
|---|---|---|---|---|---|---|---|---|---|
| 1 | 19.510 | 19347143 | 734529 | | 49.623 | | | | 49.623 |
| 2 | 24.916 | 19641037 | 778233 | S | 50.377 | | | | 50.377 |
| Total | | 38988180 | 1512762 | | 100.000 | | | | 100.000 |

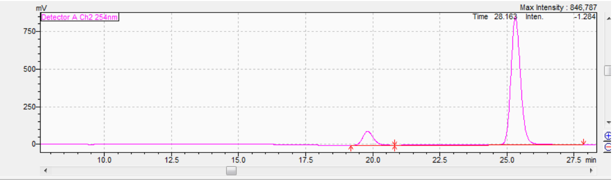

Results View - Peak Table

| Peak# | Ret. Time | Area | Height | Mark | Conc. | Unit | ID# | Name | Area% |
|---|---|---|---|---|---|---|---|---|---|
| 1 | 19.809 | 2582806 | 93052 | | 10.775 | | | | 10.775 |
| 2 | 25.315 | 21387652 | 849797 | SV | 89.225 | | | | 89.225 |
| Total | | 23970458 | 942849 | | 100.000 | | | | 100.000 |

**(*S,E*)-N,N-dimethyl-4-(3-(phenylsilyl)but-1-en-1-yl)aniline (1d)**

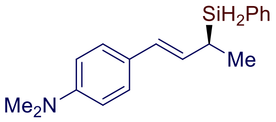

The title compound was isolated (91.0 mg, 80%, *E/Z* = >99:1) as colourless oil after chromatography on silica gel (20:1 hexane/EtOAc). $^1$H NMR (400 MHz, $CDCl_3$) δ 7.45 (dd, *J* = 7.8, 1.4 Hz, 2H), 7.29 – 7.17 (m, 3H), 7.08 (d, *J* = 8.7 Hz, 2H), 6.54 (d, *J* = 8.8 Hz, 2H), 6.08 (d, *J* = 15.9 Hz, 1H), 5.98 (dd, *J* = 15.8, 7.5 Hz, 1H), 4.23 – 4.09 (m, 2H), 2.79 (s, 6H), 2.15 – 2.03 (m, 1H), 1.15 (d, *J* = 7.2 Hz, 3H). {$^1$H}$^{13}$C NMR (101 MHz, $CDCl_3$) δ 149.7, 135.9, 131.7, 129.8, 128.9, 128.0, 127.3, 127.1, 126.7, 112.8, 40.8, 22.7, 15.5. HR-MS (APCI$^+$) *m/z* calcd for $C_{18}H_{24}NSi$, [M+H$^+$]: 282.1678, Found: 282.1681. The absolute configuration was assigned by analog to that of **1a.** Optical Rotation: $[\alpha]^{20}_D$ = -21.00 (c = 0.30 g/cm$^3$, $CHCl_3$). HPLC condition: Chiral column IB, n-hexane/i-PrOH = 99.9:0.1, flow rate = 0.35 mL/min, wavelength = 254 nm, $t_R$ = 20.2 min for minor isomer, $t_R$ = 21.7 min for major isomer.

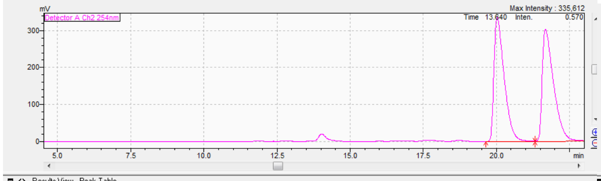

Results View - Peak Table

| Peak# | Ret. Time | Area | Height | Mark | Conc. | Unit | ID# | Name | Area% |
|---|---|---|---|---|---|---|---|---|---|
| 1 | 20.028 | 8207375 | 335755 | | 49.816 | | | | 49.816 |
| 2 | 21.678 | 8267859 | 302710 | V | 50.184 | | | | 50.184 |
| Total | | 16475234 | 638465 | | 100.000 | | | | 100.000 |

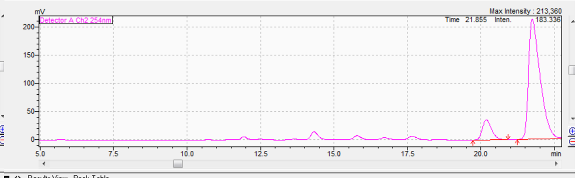

Results View - Peak Table

| Peak# | Ret. Time | Area | Height | Mark | Conc. | Unit | ID# | Name | Area% |
|---|---|---|---|---|---|---|---|---|---|
| 1 | 20.182 | 782170 | 35673 | | 11.937 | | | | 11.937 |
| 2 | 21.747 | 5770292 | 211846 | | 88.063 | | | | 88.063 |
| Total | | 6552462 | 247519 | | 100.000 | | | | 100.000 |

**(*S,E*)-(4-(4-methoxyphenyl)but-3-en-2-yl)(phenyl)silane (1o)**

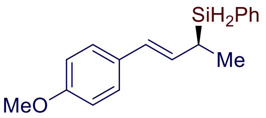

The reaction was stirred for 12 h at 24 °C. The title compound was isolated (42.0 mg, 61%, *E/Z* = >99:1.) as colourless oil after chromatography on silica gel (100:1 hexane/EtOAc). $^1$H NMR (400 MHz, $CDCl_3$) δ 7.62–7.51 (m, 2H), 7.45–7.31 (m, 3H), 7.26–7.22 (m, 2H), 6.85–6.79 (m, 2H), 6.23 (d, *J* = 16.0 Hz, 1H), 6.16 (dd, *J* = 15.9, 6.9 Hz, 1H), 4.35–4.19 (m, 2H), 3.80 (s, 3H), 2.28–2.19 (m, 1H), 1.28 (d, *J* = 7.2 Hz, 3H). {$^1$H}$^{13}$C NMR (101 MHz, $CDCl_3$) δ 158.7, 135.9, 131.5, 131.1, 131.0, 129.9, 128.1, 127.0, 126.8, 114.1, 55.5, 22.8, 15.4. HR-MS (APCI$^+$) *m/z* calcd for $C_{17}H_{21}OSi$, [M+H$^+$]: 269.1362, Found: 269.1354. Optical Rotation: $[\alpha]^{20}_D$ = -21.00 (c = 0.30 g/cm$^3$, $CHCl_3$). The absolute configuration was assigned by oxidizing it to the corresponding allylic alcohol (**1o''**).

**($S$,$E$)-4-(4-methoxyphenyl)but-3-en-2-ol (1o''')**

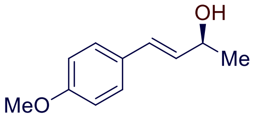


The title compound was isolated (31.7 mg, 73%, 90:10 er) as colourless oil after chromatography on silica gel (10:1 hexane/EtOAc). $^{1}$H NMR (400 MHz, $CDCl_3$) δ 7.35–7.28 (m, 2H), 6.88–6.83 (m, 2H), 6.51 (d, $J$ = 15.9 Hz, 1H), 6.13 (dd, $J$ = 15.9, 6.6 Hz, 1H), 4.47 (p, $J$ = 6.3 Hz, 1H), 3.81 (s, 3H), 1.37 (d, $J$ = 6.4 Hz, 3H). {$^{1}$H}$^{13}$C NMR (101 MHz, $CDCl_3$) δ 159.5, 131.6, 129.6, 129.2, 127.8, 114.2, 69.3, 55.5, 23.6. Optical Rotation: $[\alpha]^{20}_{D}$ = -10.70 (c = 0.30 g/cm$^3$, $CHCl_3$). The absolute configuration was assigned by analog to that of **1a''**. HPLC condition: Chiral column IB, n-hexane/i-PrOH = 93:7, flow rate = 0.35 mL/min, wavelength = 254 nm, $t_R$ = 30.4 min for minor isomer, $t_R$ = 31.5 min for major isomer.

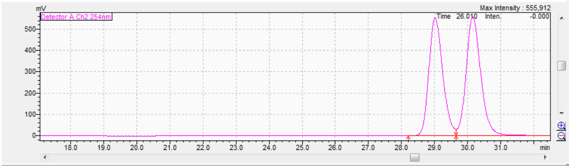


| Peak# | Ret. Time | Area | Height | Mark | Conc. | Unit | ID# | Name | Area% |
|---|---|---|---|---|---|---|---|---|---|
| 1 | 29.015 | 16070830 | 553652 | | 48.277 | | | | 48.277 |
| 2 | 30.154 | 17218053 | 555750 | SV | 51.723 | | | | 51.723 |
| Total | | 33288883 | 1109402 | | 100.000 | | | | 100.000 |

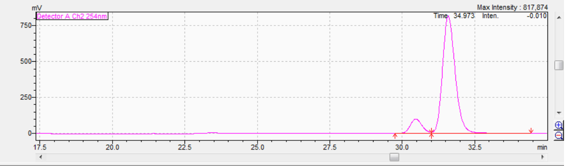


| Peak# | Ret. Time | Area | Height | Mark | Conc. | Unit | ID# | Name | Area% |
|---|---|---|---|---|---|---|---|---|---|
| 1 | 30.465 | 2852097 | 100758 | | 10.312 | | | | 10.312 |
| 2 | 31.585 | 24806500 | 818012 | V | 89.688 | | | | 89.688 |
| Total | | 27658597 | 918769 | | 100.000 | | | | 100.000 |

## Mechanistic studies

### Procedure for Deuterium-labeling Experiments

Note: $PhSiD_3$[6] was synthesized based on previously reported procedure.

In an Ar-filled glovebox, a mixture of $Co(acac)_2$ (0.5 mg, 2.0 μmol) and xantphos (1.2 mg, 2.0 μmol) in THF (0.5 mL) was added into a 4-mL screw-capped vial containing a magnetic stirring bar. The resulting mixture was stirred for 2 mins prior adding $PhSiD_3$ (27.8 mg, 0.25 mmol) and 1,3-dienes (0.2 mmol) successively. The vial was removed from the glove box, and the mixture was stirred at room temperature for 4 hours. After that, the crude reaction mixture was concentrated under vacuum and the residue was purified by flash column chromatography using a mixture of ethyl acetate and hexane (100:1) as eluent. The details and characterization data of the products are stated below. Equimolar of chloroform-$d$ was added as the internal standard for $^{2}$H NMR analysis.

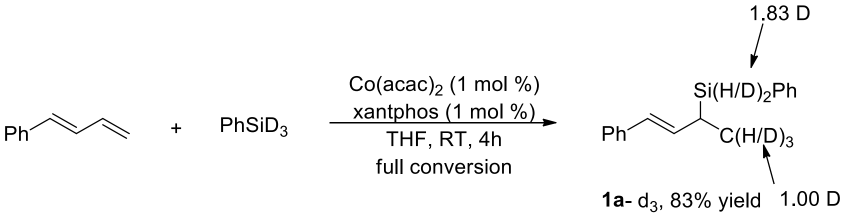


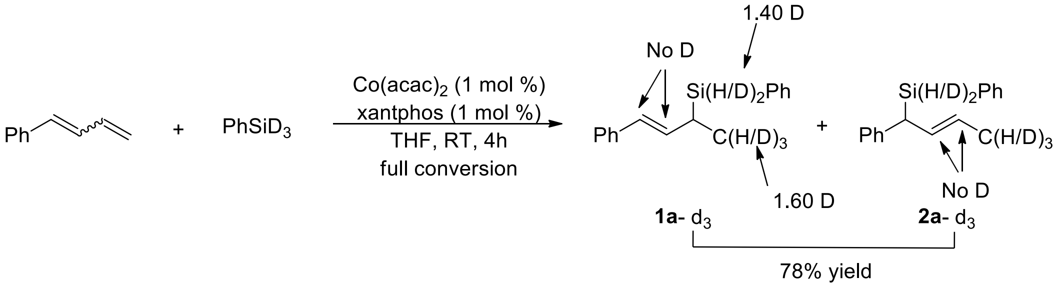

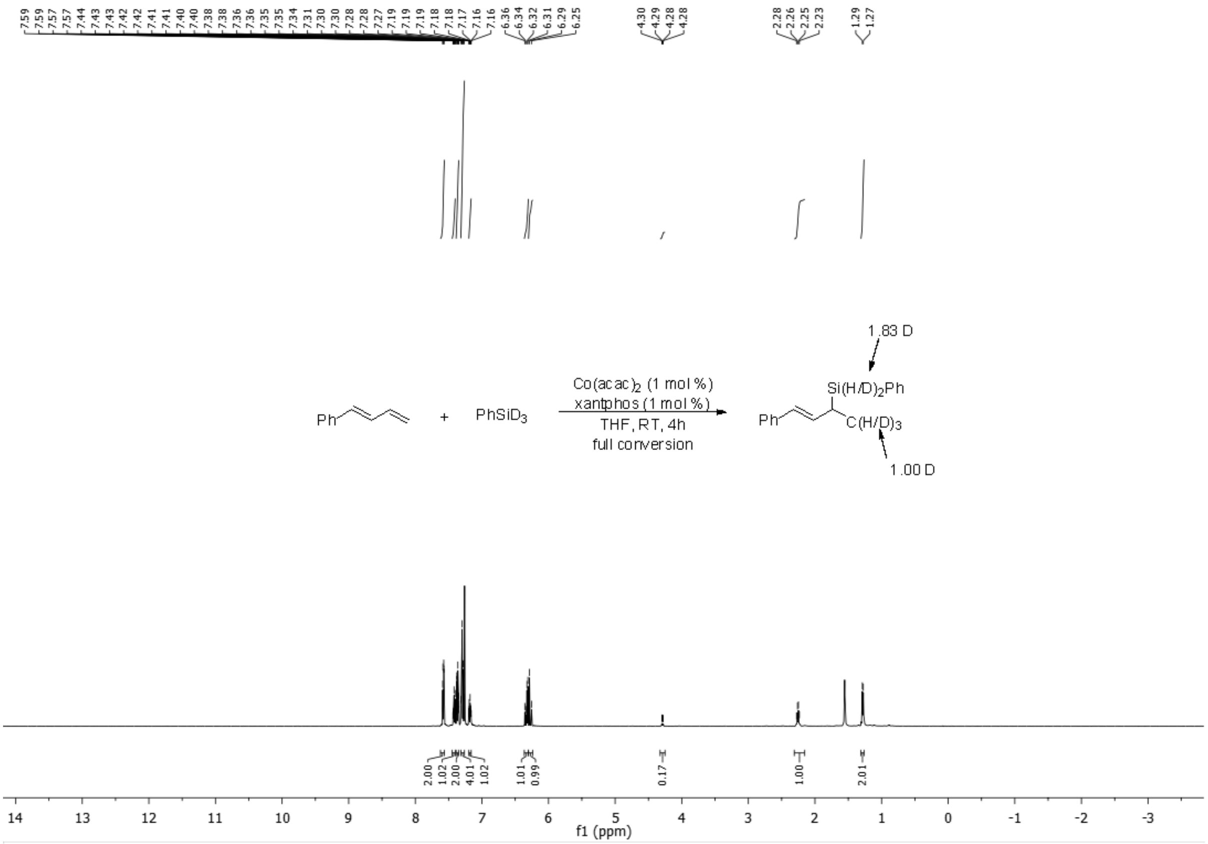
1.83 D
Co(acac)2 (1 mol %)
xantphos (1 mol %)
THF, RT, 4h
full conversion
Ph
PhSiD3
Si(H/D)2Ph
C(H/D)3
1.00 D
f1 (ppm)

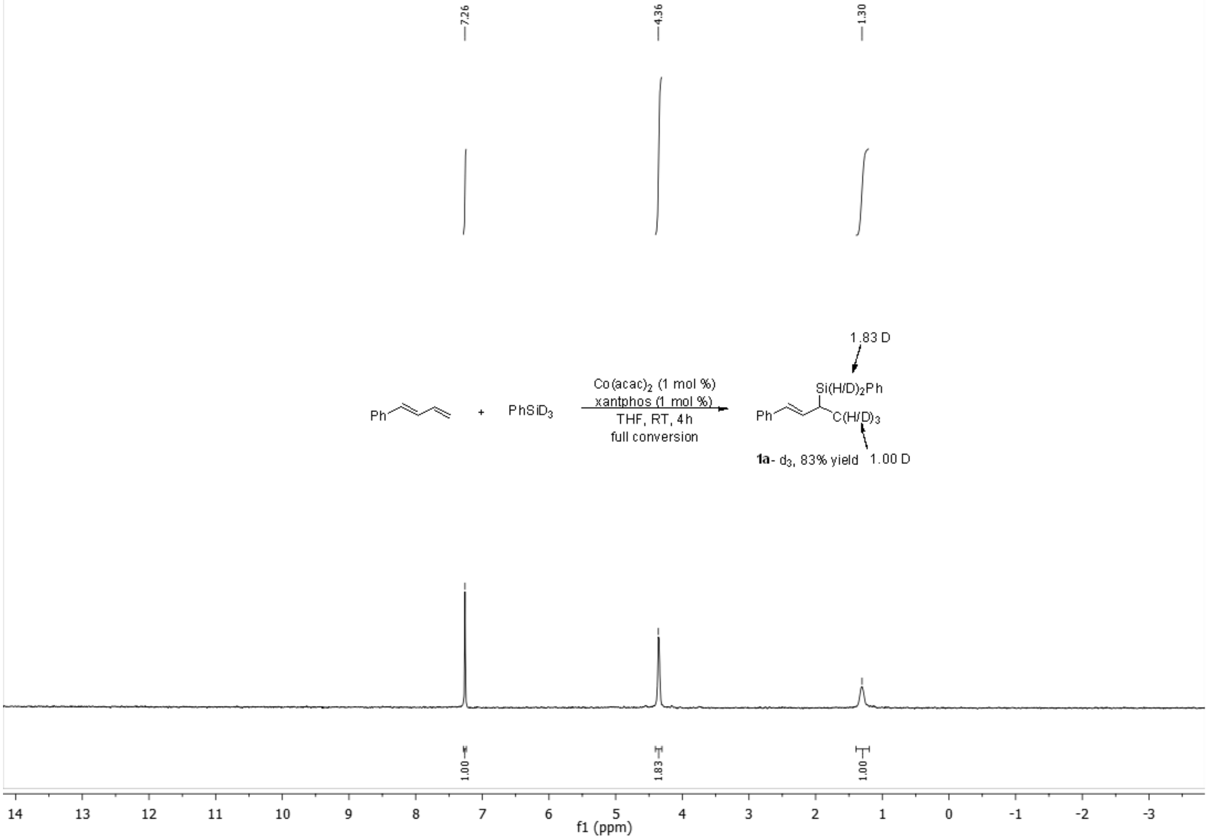
1.83 D
Co(acac)2 (1 mol %)
xantphos (1 mol %)
THF, RT, 4h
full conversion
Ph
PhSiD3
Si(H/D)2Ph
C(H/D)3
1a- d3, 83% yield
1.00 D
f1 (ppm)

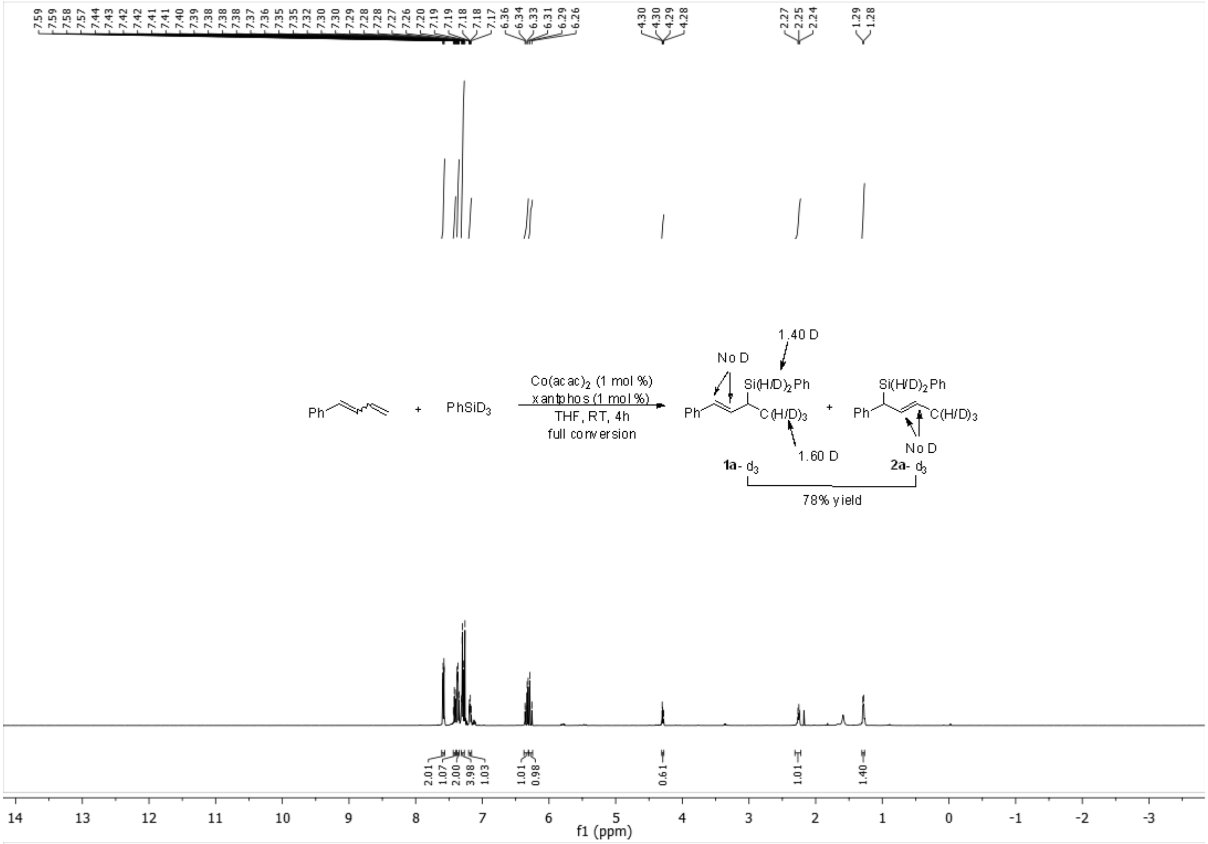
Co(acac)2 (1 mol %)
xantphos (1 mol %)
THF, RT, 4h
full conversion
Ph
PhSiD3
No D
1.40 D
Si(H/D)2Ph
C(H/D)3
1.60 D
1a- d3
2a- d3
78% yield
f1 (ppm)

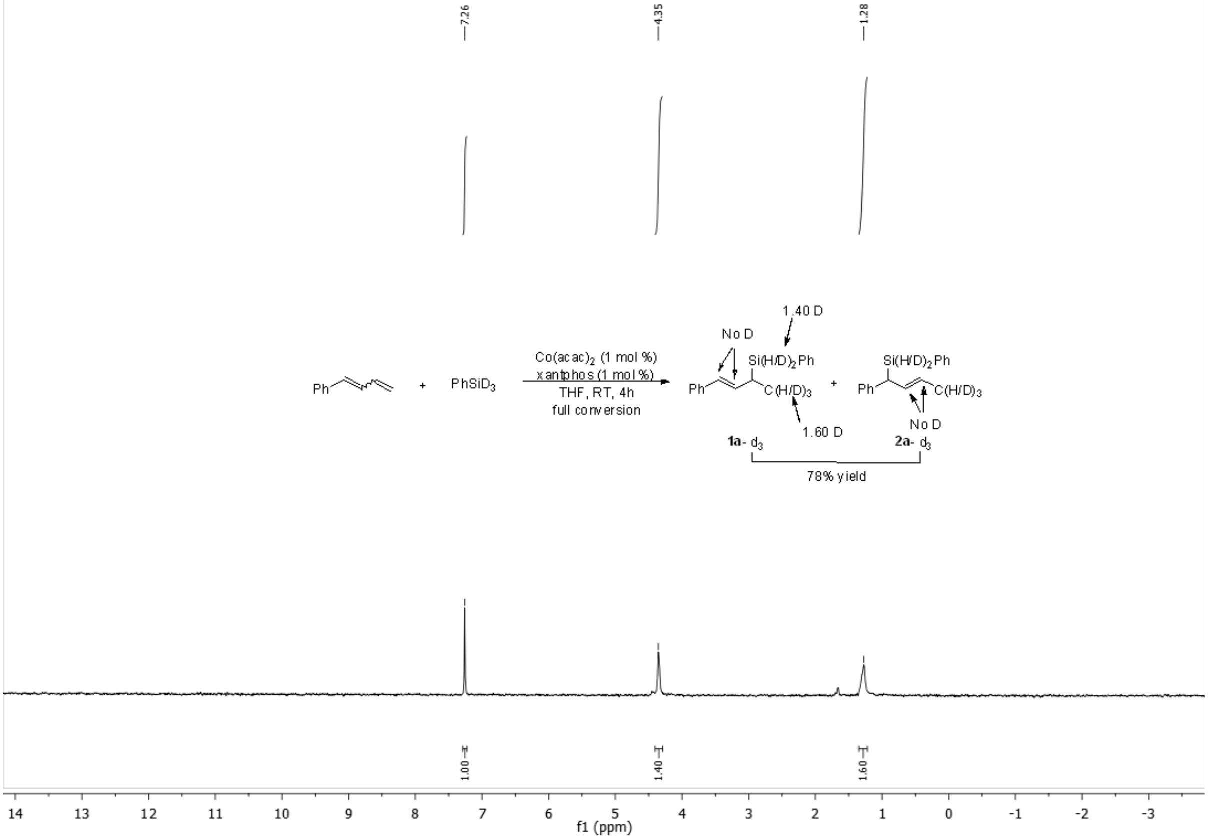
Co(acac)2 (1 mol %)
xantphos (1 mol %)
THF, RT, 4h
full conversion
Ph
PhSiD3
No D
1.40 D
Si(H/D)2Ph
C(H/D)3
1.60 D
1a- d3
2a- d3
78% yield
f1 (ppm)

## Reaction monitoring

### General Procedure for Reaction monitoring of (*E*/Z)-1-(buta-1,3-dien-1-yl)-4-methoxybenzene with phenylsilane

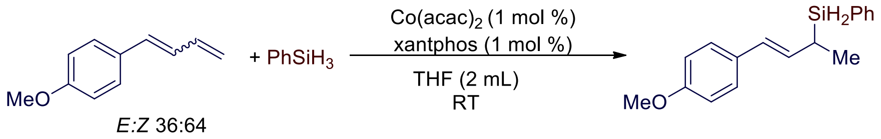


In an Ar-filled glovebox, a mixture of (1.0 mg, 4.0 $\mu$mol) and xantphos (2.3 mg, 4.0 $\mu$mol) in THF (2 mL) was added into a 4-mL screw-capped vial containing a magnetic stirring bar. The resulting mixture was stirred for 2 mins prior adding phenylsilane (54.1 mg, 0.5 mmol) and 1-(buta-1,3-dien-1-yl)-4-methoxybenzene (64.1 mg, 0.4 mmol) successively. Lastly, tridecane (27.7 mg, 0.15 mmol) was added into the reaction mixture as an internal standard. The mixture was stirred at room temperature. A GC analysis was done for the crude mixture for every 5 minutes to monitor the reaction. It was found that the (*E*)-1-(buta-1,3-dien-1-yl)-4-methoxybenzene was consumed at a significantly higher rate than (*Z*)-1-(buta-1,3-dien-1-yl)-4-methoxybenzene.

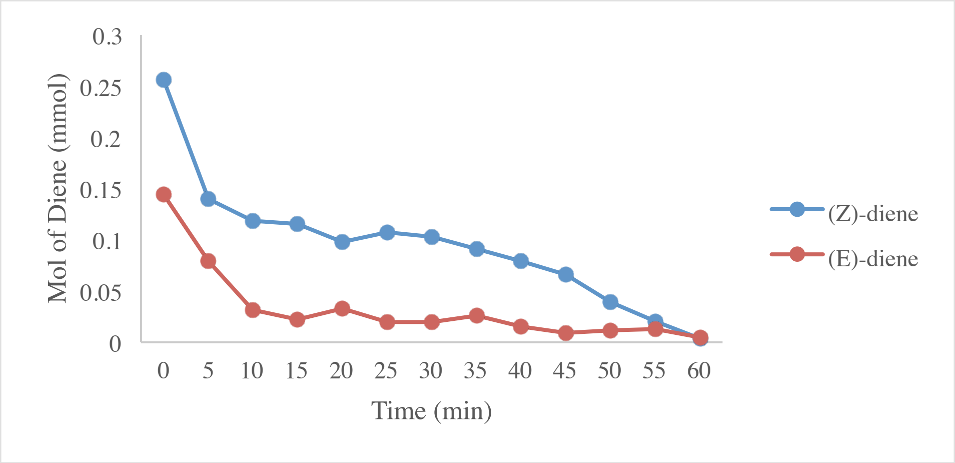


### References


1. C. H. Schuster, T. Diao, I. Pappas and P. J. Chirik, *ACS Catalysis*, 2016, **6**, 2632-2636.
2. K. B. Urkalan and M. S. Sigman, *Angewandte Chemie International Edition*, 2009, **48**, 3146-3149.
3. G. Battistuzzi, S. Cacchi and G. Fabrizi, *Organic Letters*, 2003, **5**, 777-780.
4. P. Röse, S. Emge, J.-i. Yoshida and G. Hilt, *Beilstein Journal of Organic Chemistry*, 2015, **11**, 174-183.
5. L. Sun, Y. Guo, G. Peng and C. Li, *Synthesis*, 2008, **2008**, 3487-3491.
6. V. Avrorin, G. Fominykh, I. Ignat'ev, E. Sinotova and T. Kochina, *Russian Journal of General Chemistry*, 2014, **84**, 2125-2129.

**NMR Spectra**

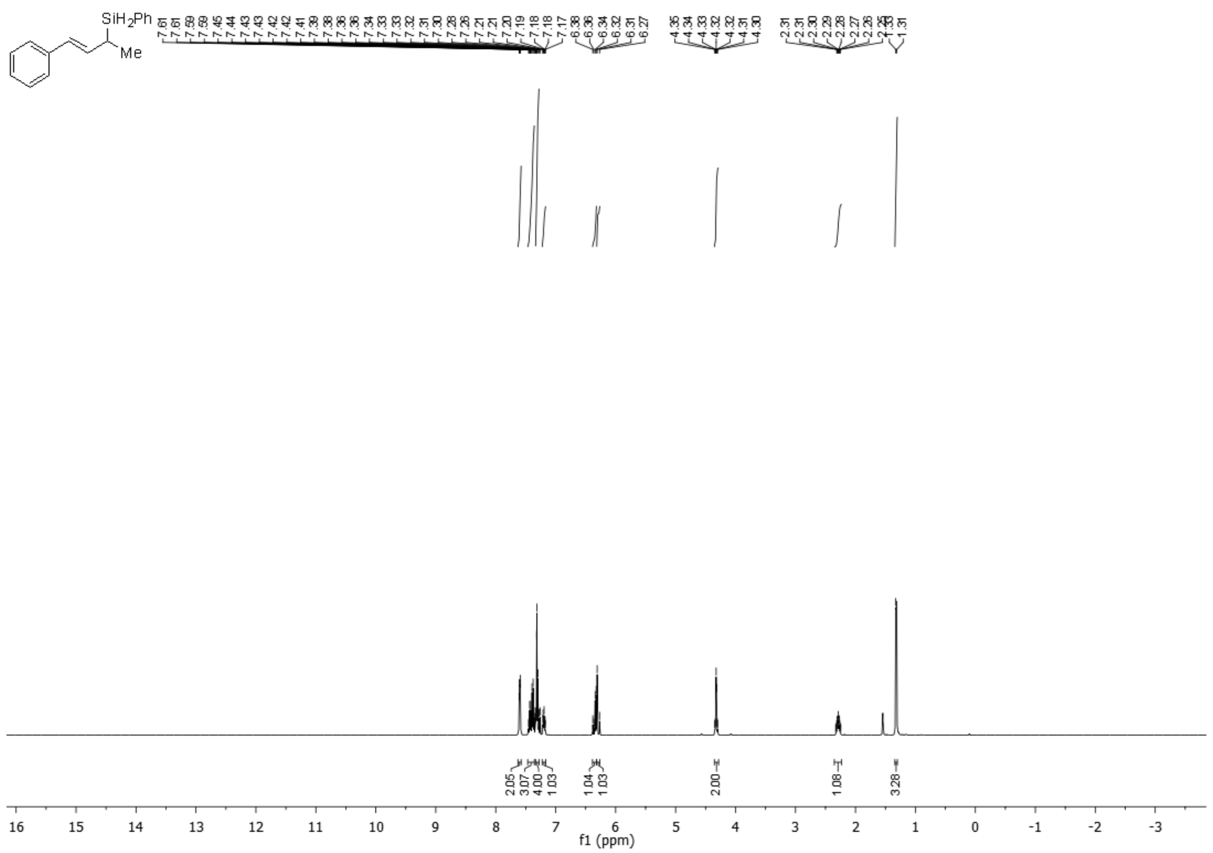

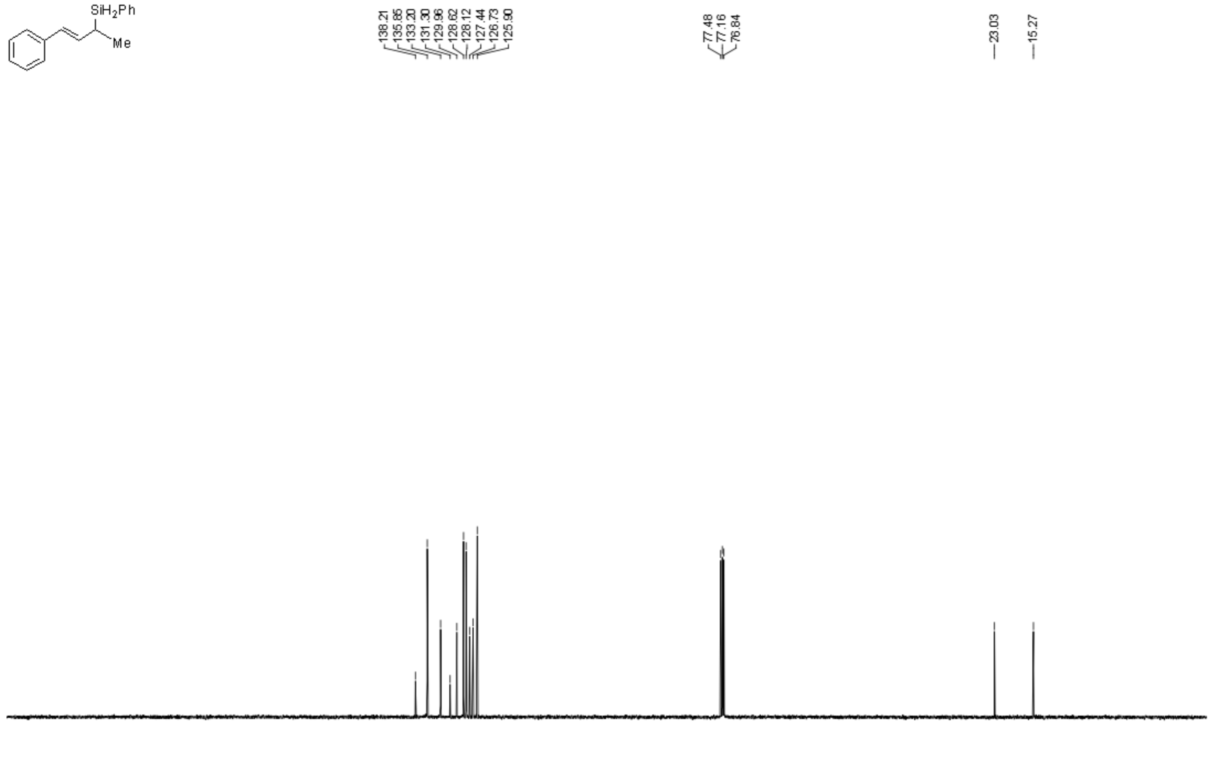

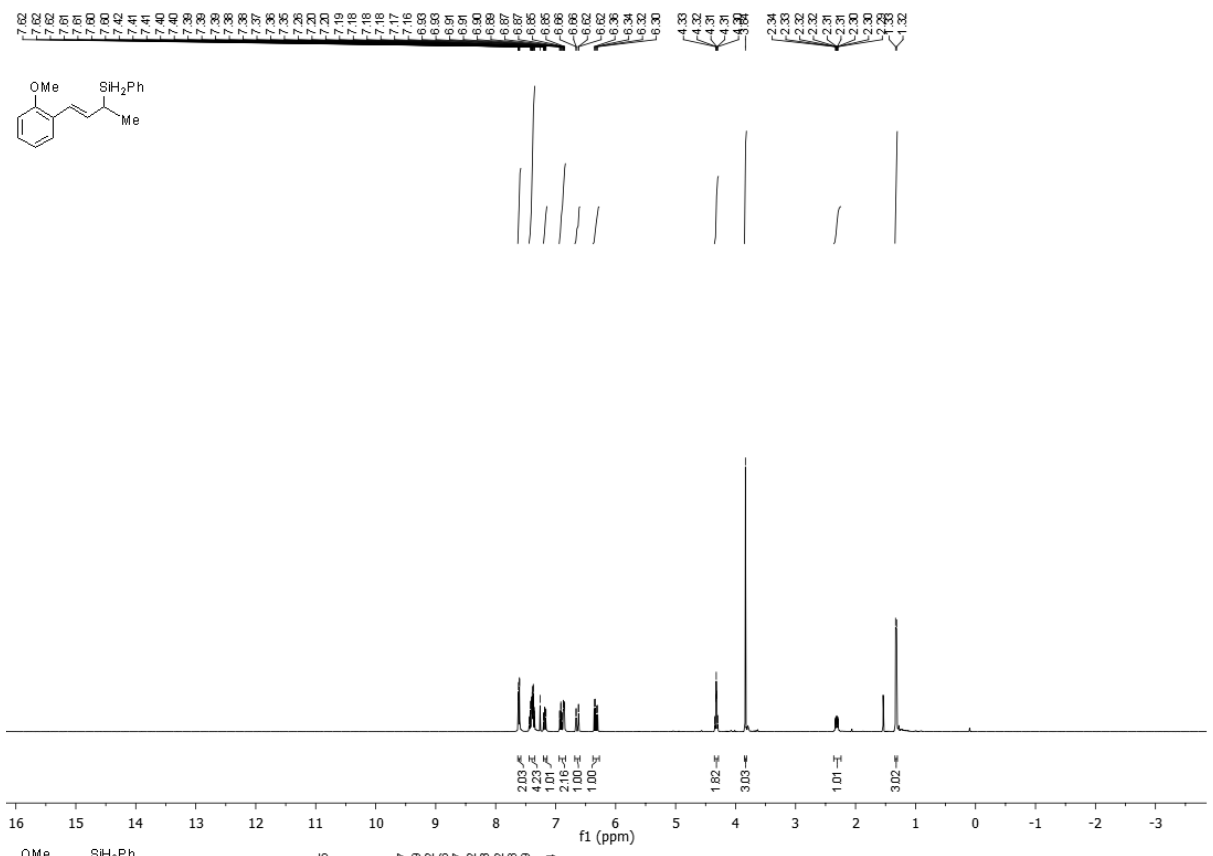

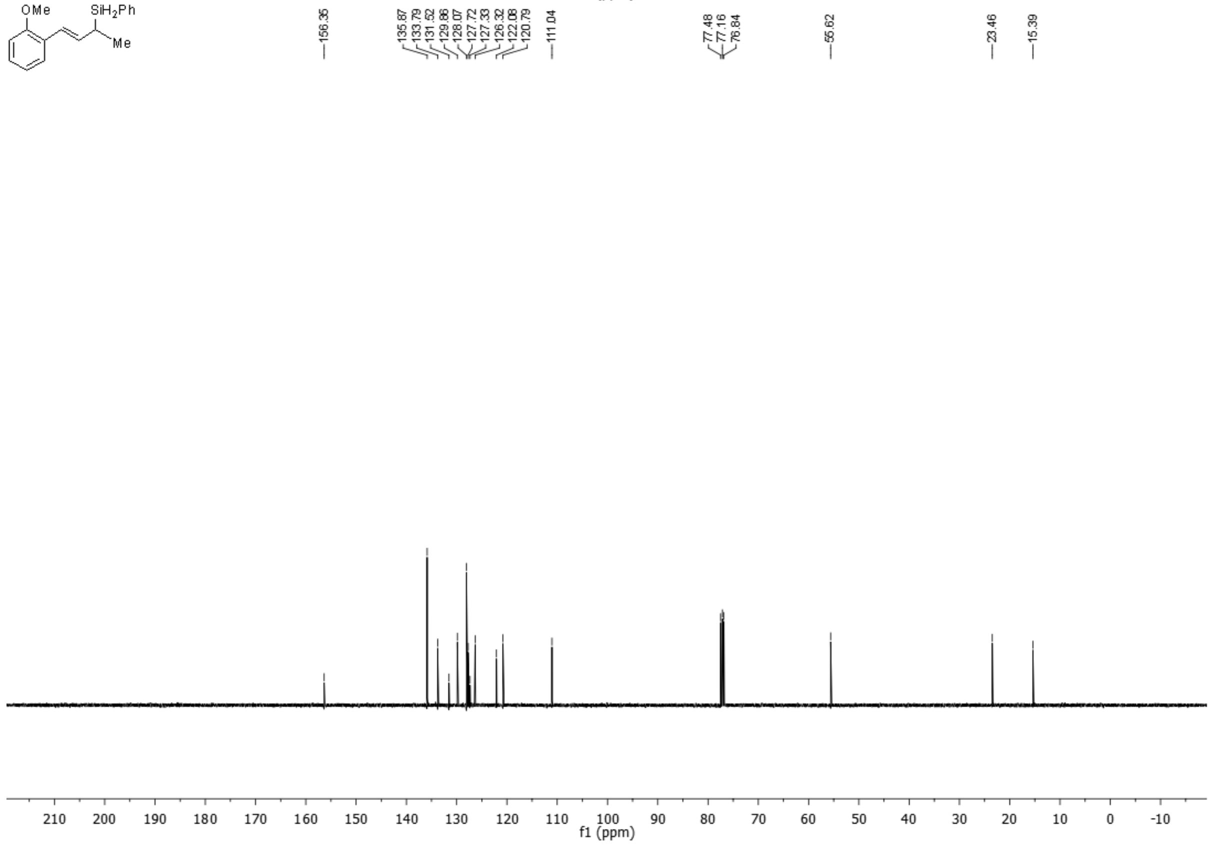

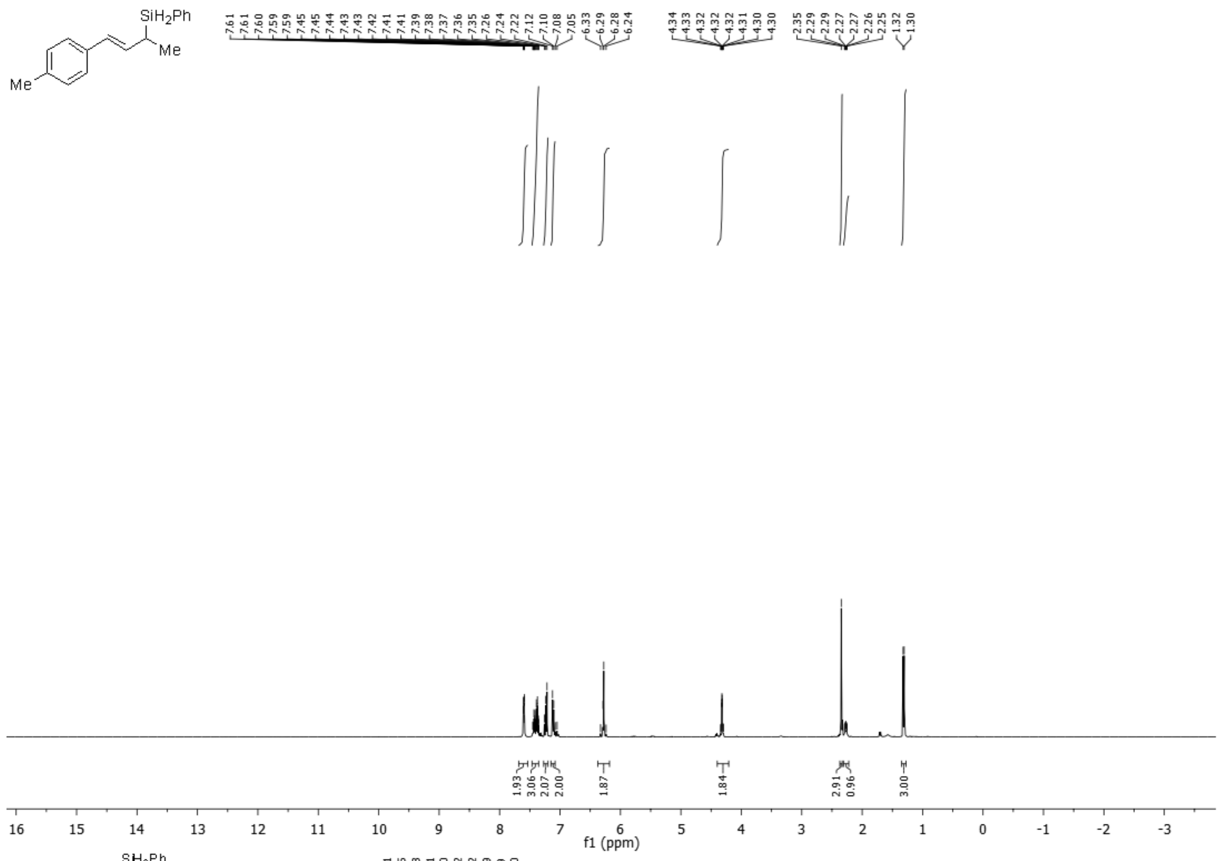
SiH2Ph
Me
Me
f1 (ppm)

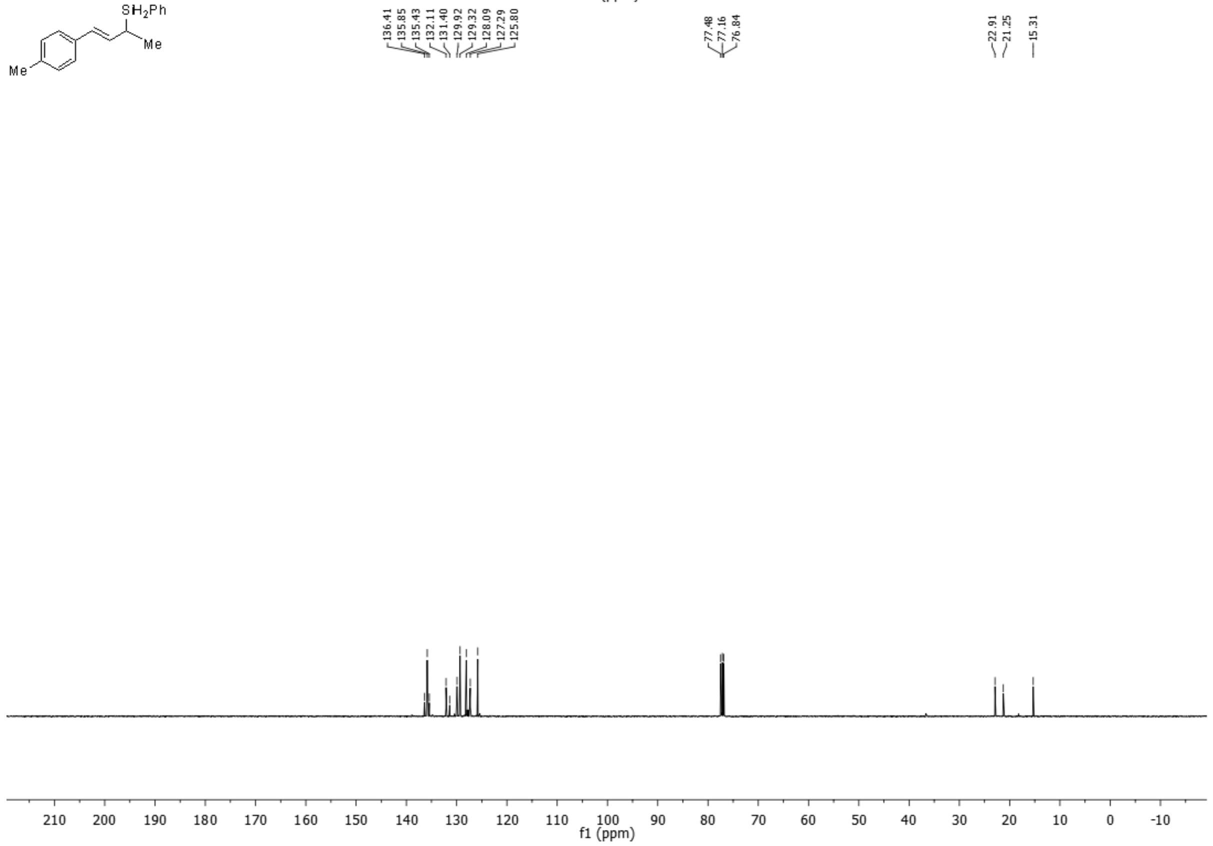
SiH2Ph
Me
Me
f1 (ppm)

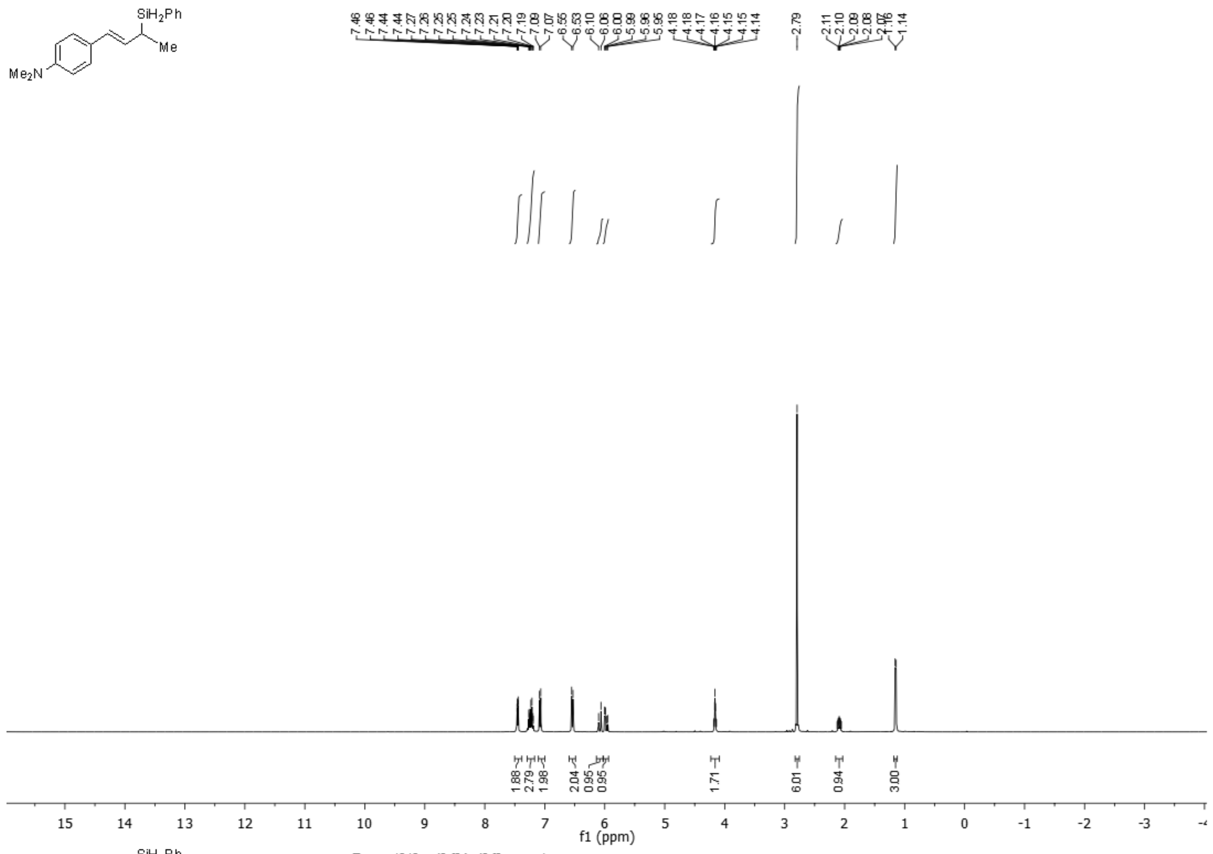
SiH2Ph
Me
Me2N
f1 (ppm)

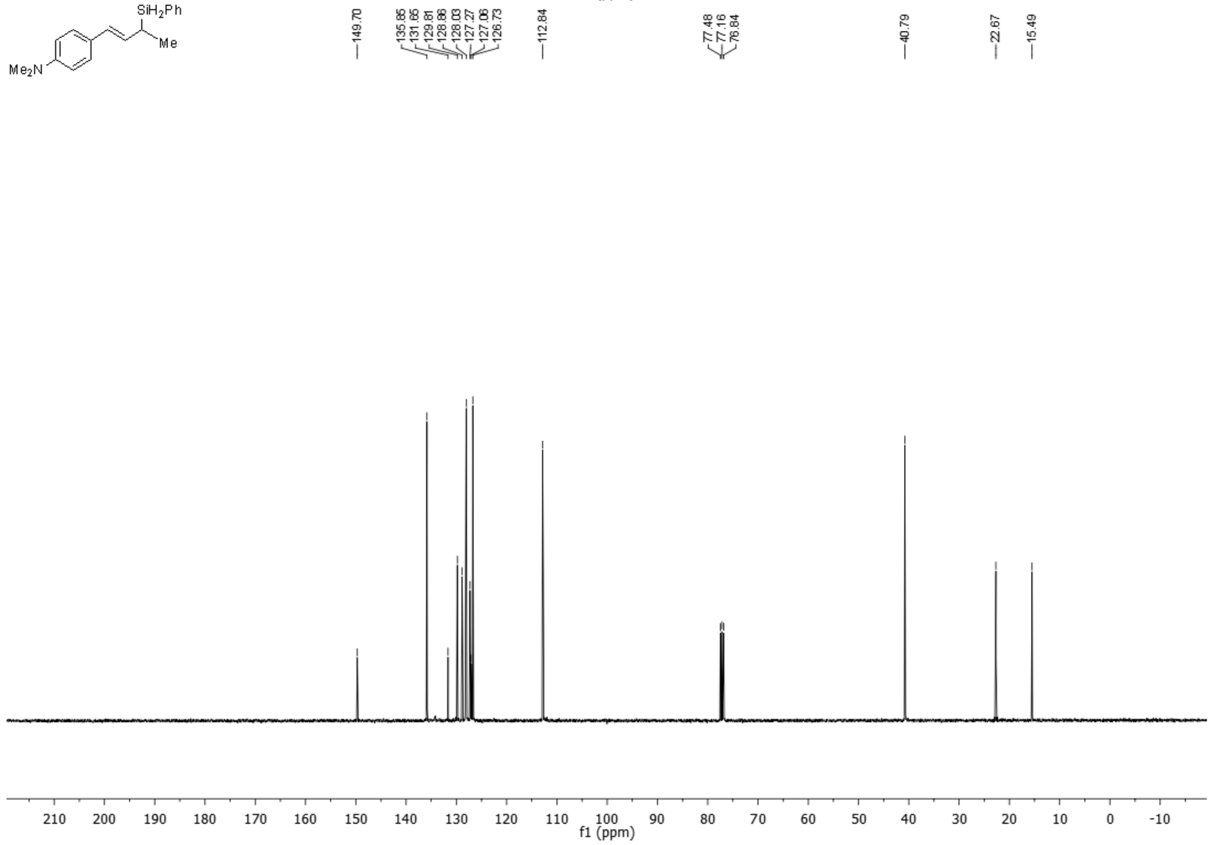
SiH2Ph
Me
Me2N
f1 (ppm)

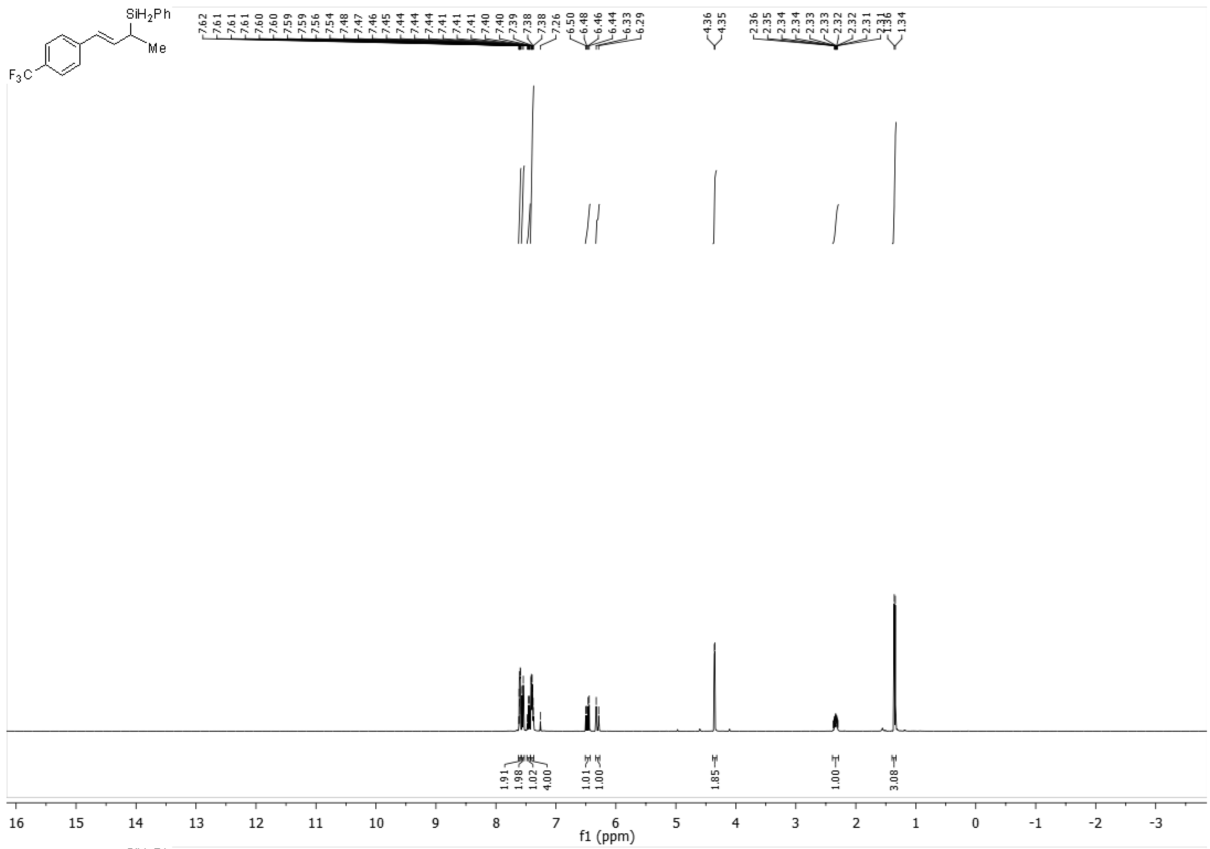
SiH2Ph
Me
F3C
7.62 7.61 7.61 7.60 7.59 7.59 7.56 7.54 7.48 7.47 7.46 7.45 7.44 7.44 7.44 7.41 7.41 7.40 7.39 7.38 7.38 7.26 6.50 6.48 6.46 6.44 6.33 6.29
4.36 4.35
2.36 2.35 2.34 2.34 2.33 2.33 2.32 2.32 2.31 2.31 1.36 1.34
1.91 1.98 1.02 4.00 1.01 1.00 1.85 1.00 3.08
f1 (ppm)

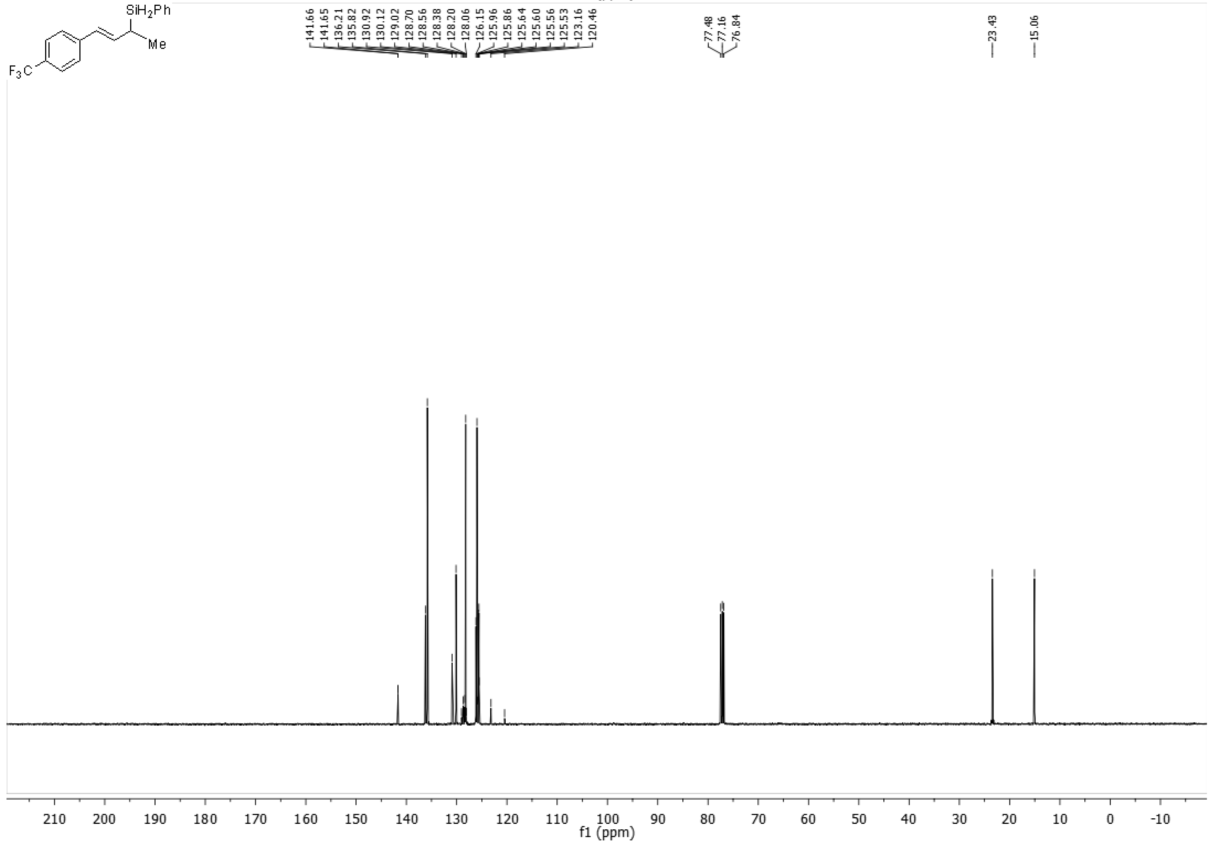
SiH2Ph
Me
F3C
141.66 141.65 136.21 135.82 130.92 130.12 129.02 128.70 128.56 128.38 128.20 128.06 126.15 125.96 125.86 125.64 125.60 125.56 125.53 123.16 120.46
77.48 77.16 76.84
23.43
15.06
f1 (ppm)

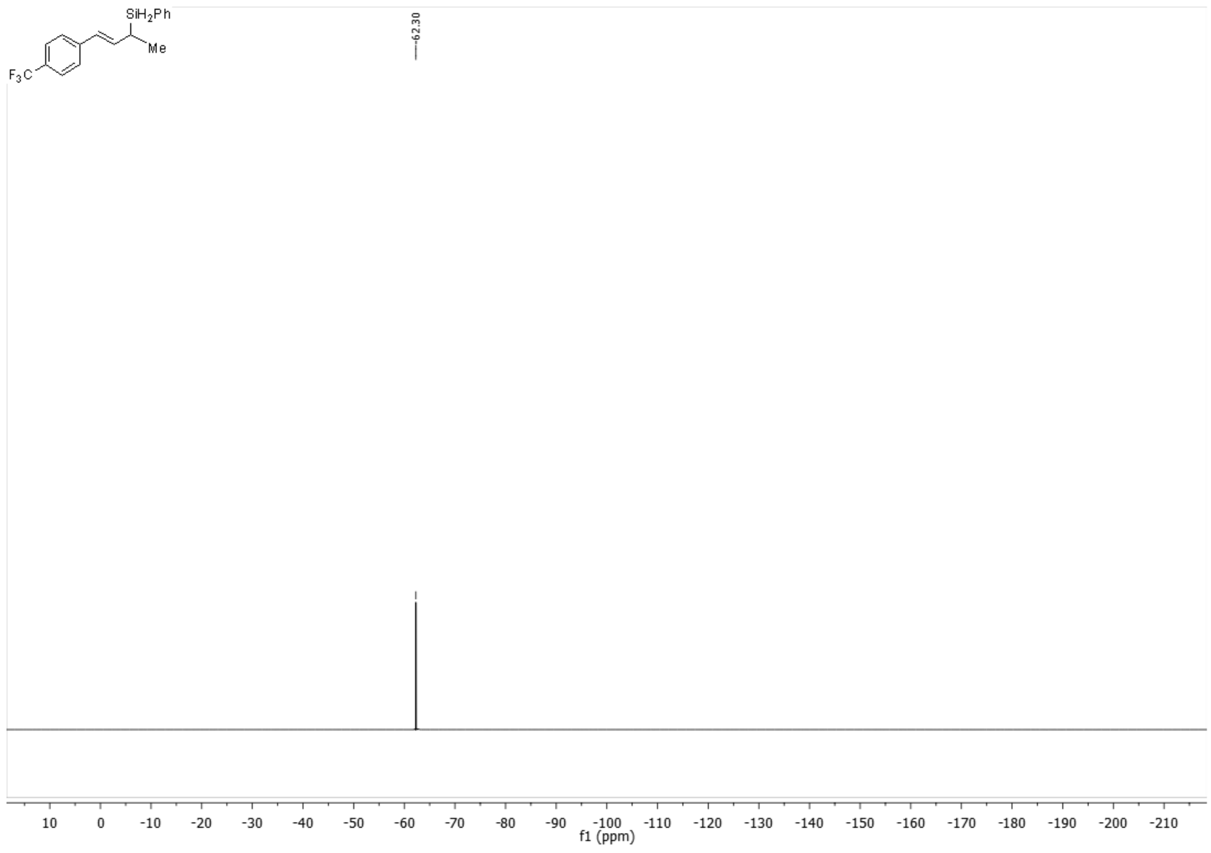
SiH2Ph
Me
F3C
-62.30
10 0 -10 -20 -30 -40 -50 -60 -70 -80 -90 -100 -110 -120 -130 -140 -150 -160 -170 -180 -190 -200 -210
f1 (ppm)

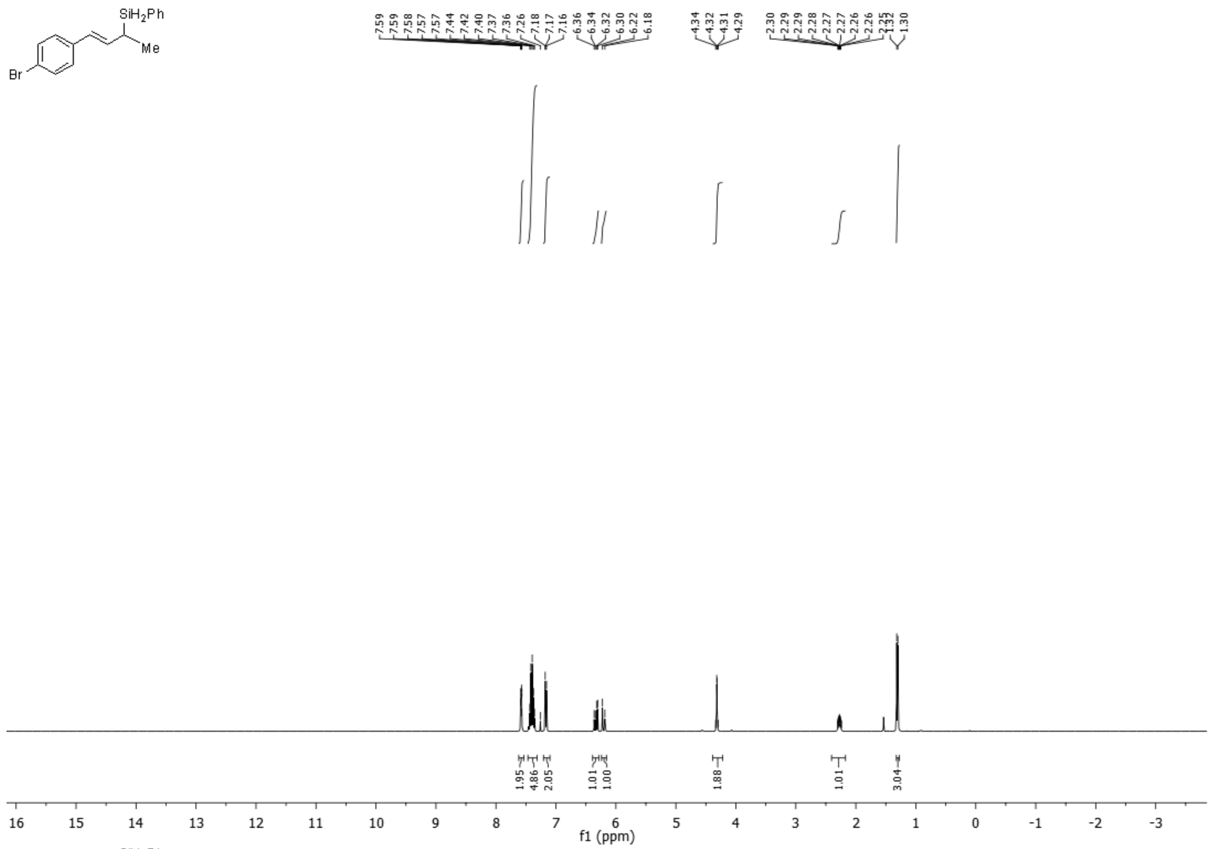
SiH2Ph
Me
Br
f1 (ppm)

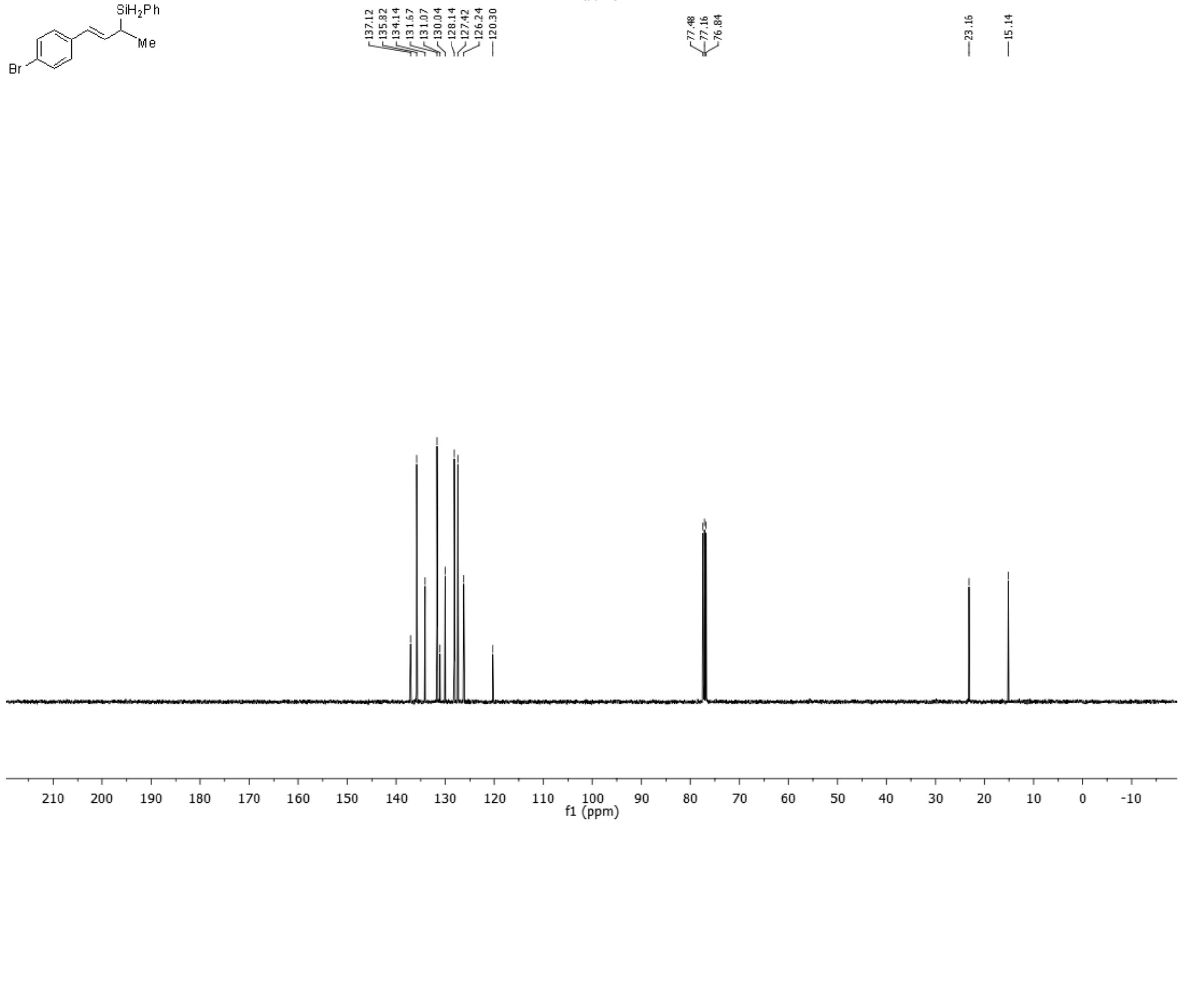
SiH2Ph
Me
Br
f1 (ppm)

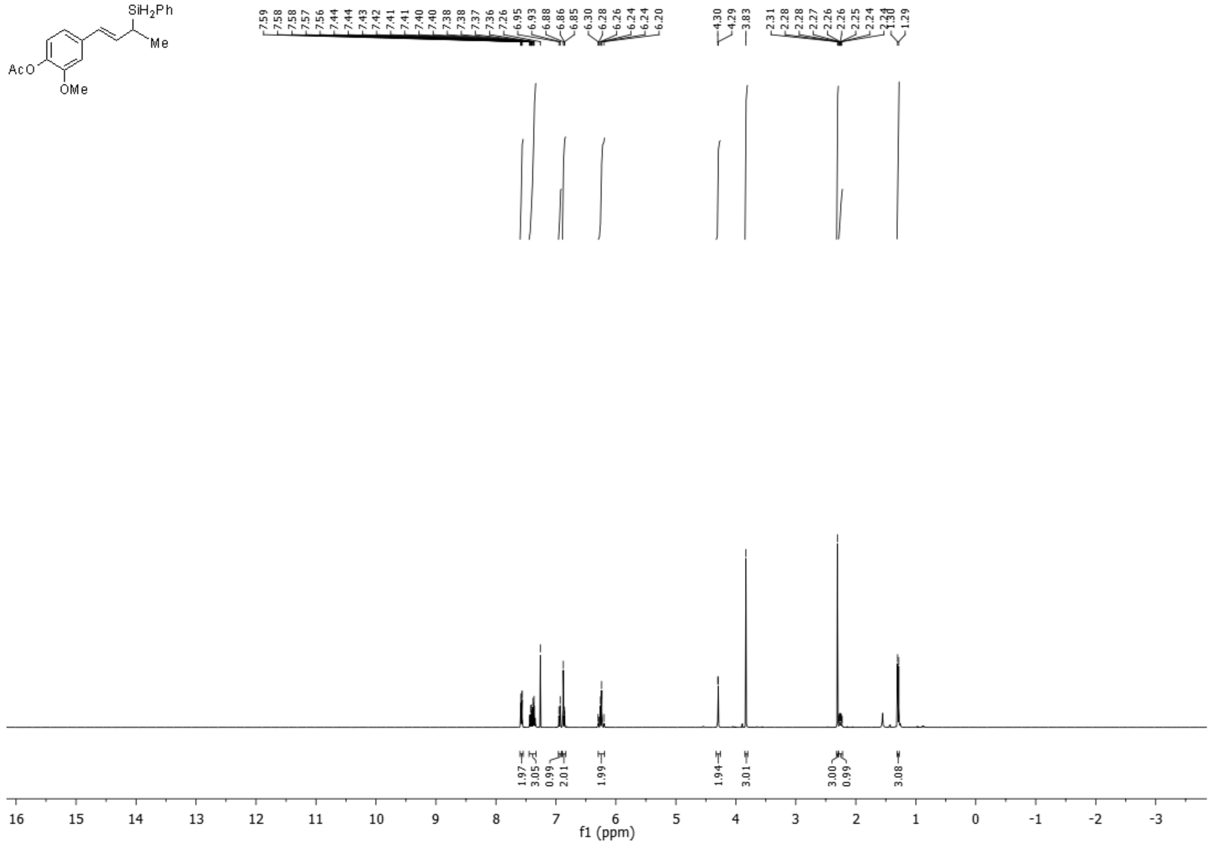

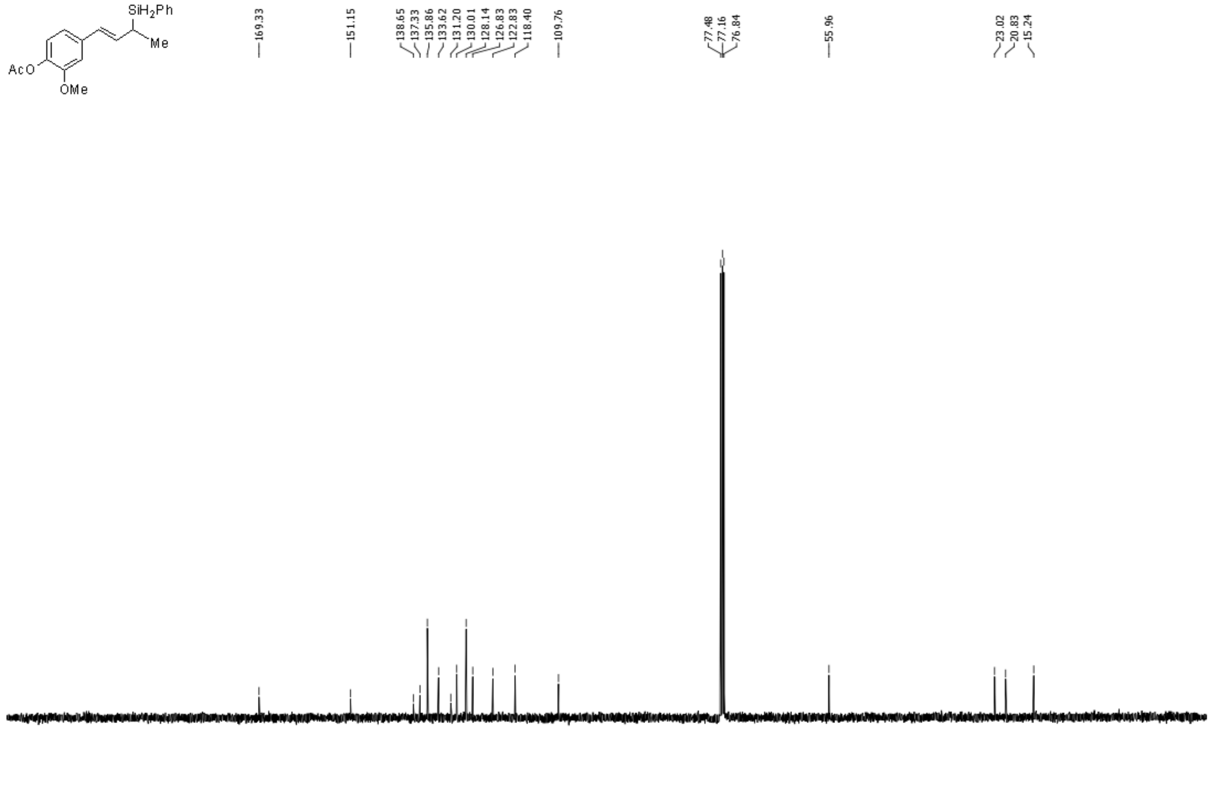

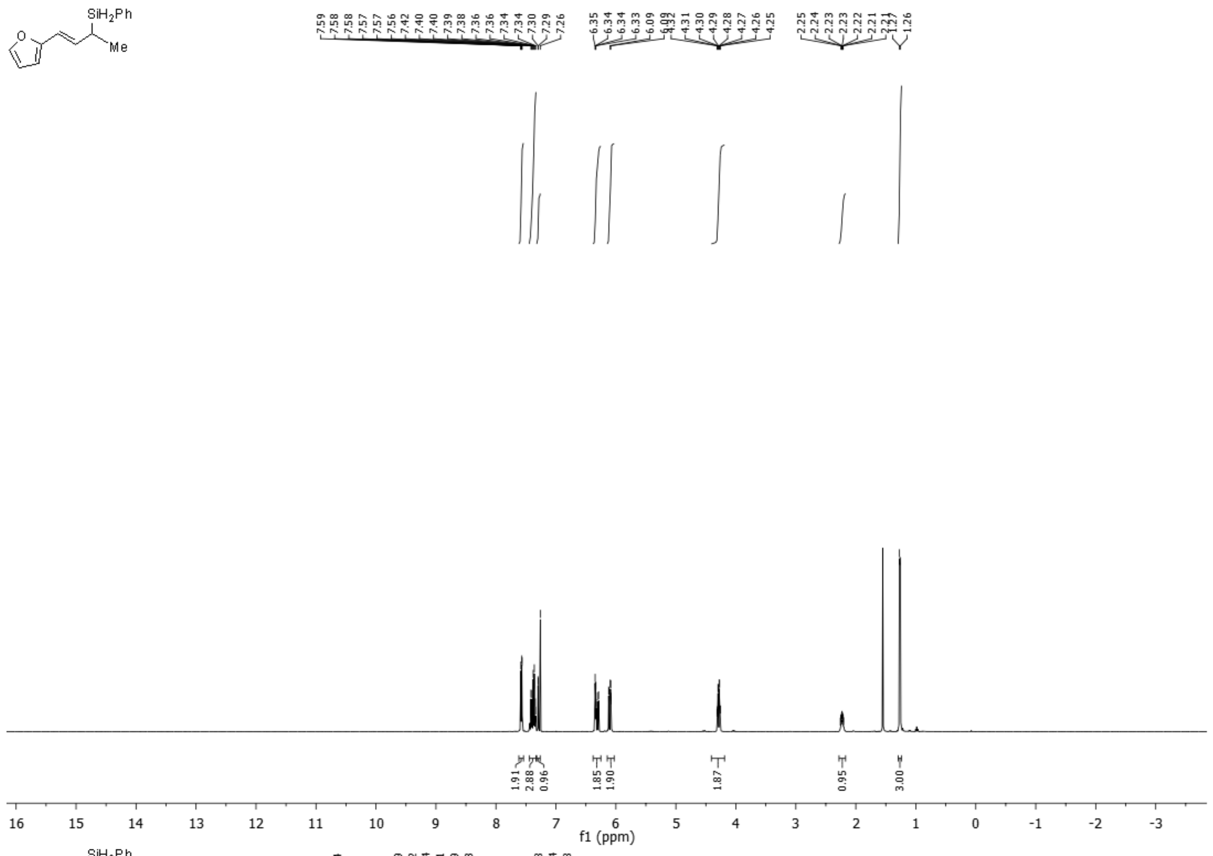
SiH2Ph
O
Me
7.59
7.58
7.58
7.57
7.57
7.56
7.42
7.40
7.40
7.39
7.38
7.36
7.36
7.34
7.34
7.30
7.29
7.26
6.35
6.34
6.34
6.33
4.31
4.30
4.29
4.28
4.27
4.26
4.25
2.25
2.24
2.23
2.23
2.22
2.21
1.27
1.26
1.91
2.88
0.96
1.85
1.90
1.87
0.95
3.00
16 15 14 13 12 11 10 9 8 7 6 5 4 3 2 1 0 -1 -2 -3
f1 (ppm)

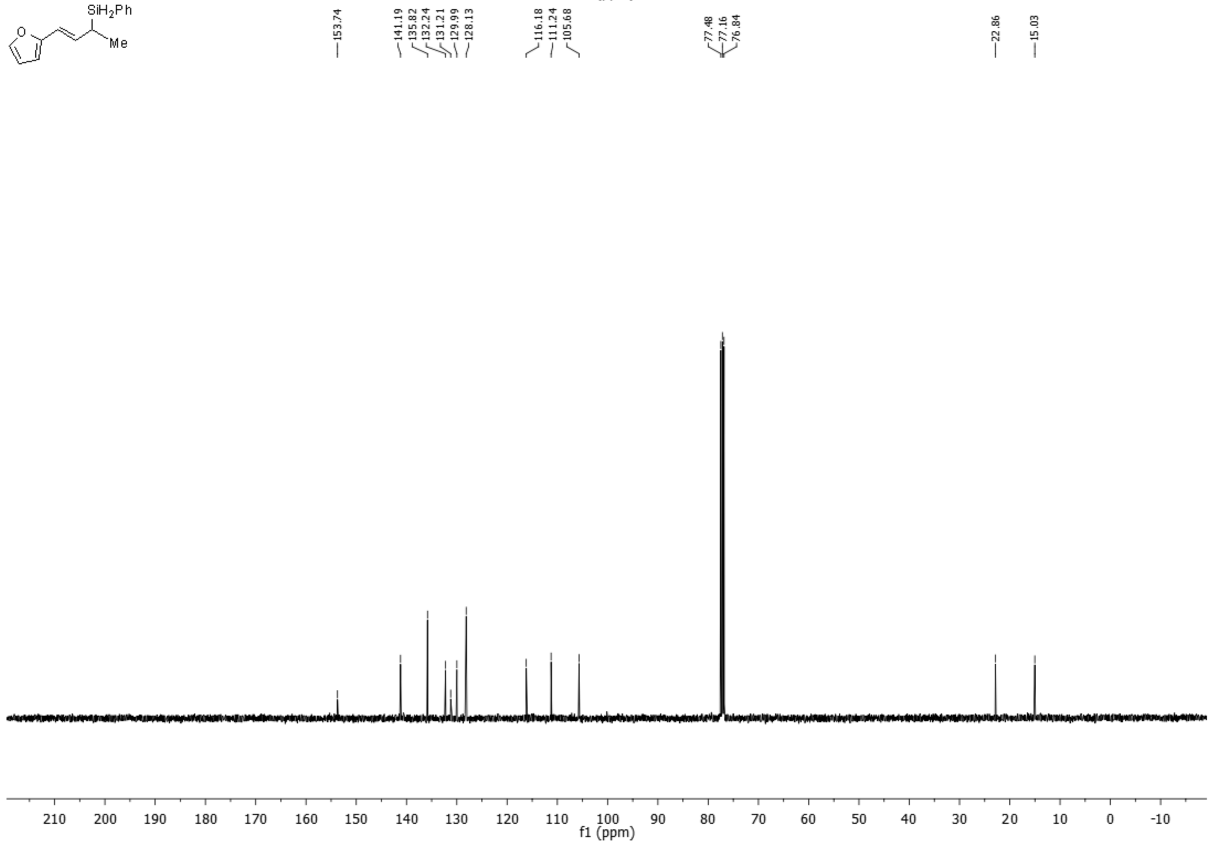
SiH2Ph
O
Me
153.74
141.19
135.82
132.24
131.21
129.99
128.13
116.18
111.24
105.68
77.48
77.16
76.84
22.86
15.03
210 200 190 180 170 160 150 140 130 120 110 100 90 80 70 60 50 40 30 20 10 0 -10
f1 (ppm)

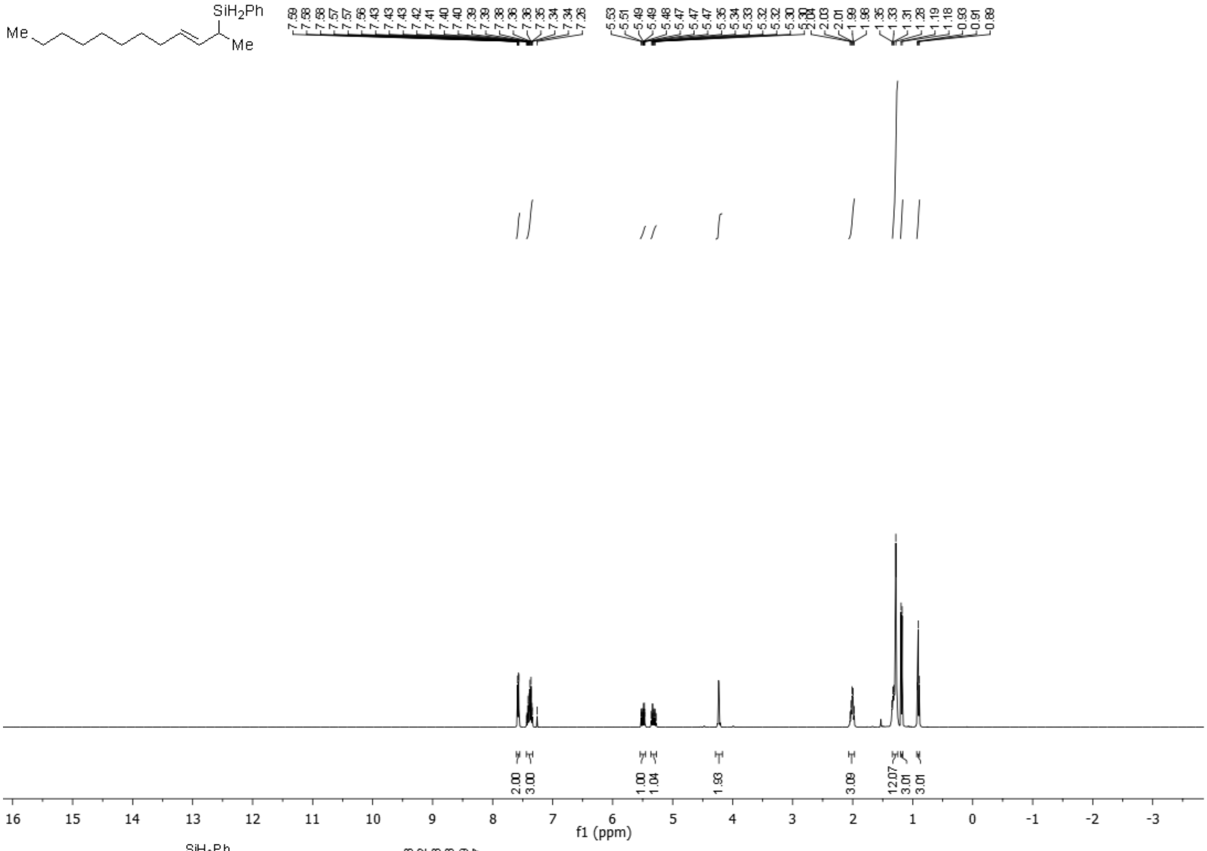
SiH2Ph
Me
Me
f1 (ppm)

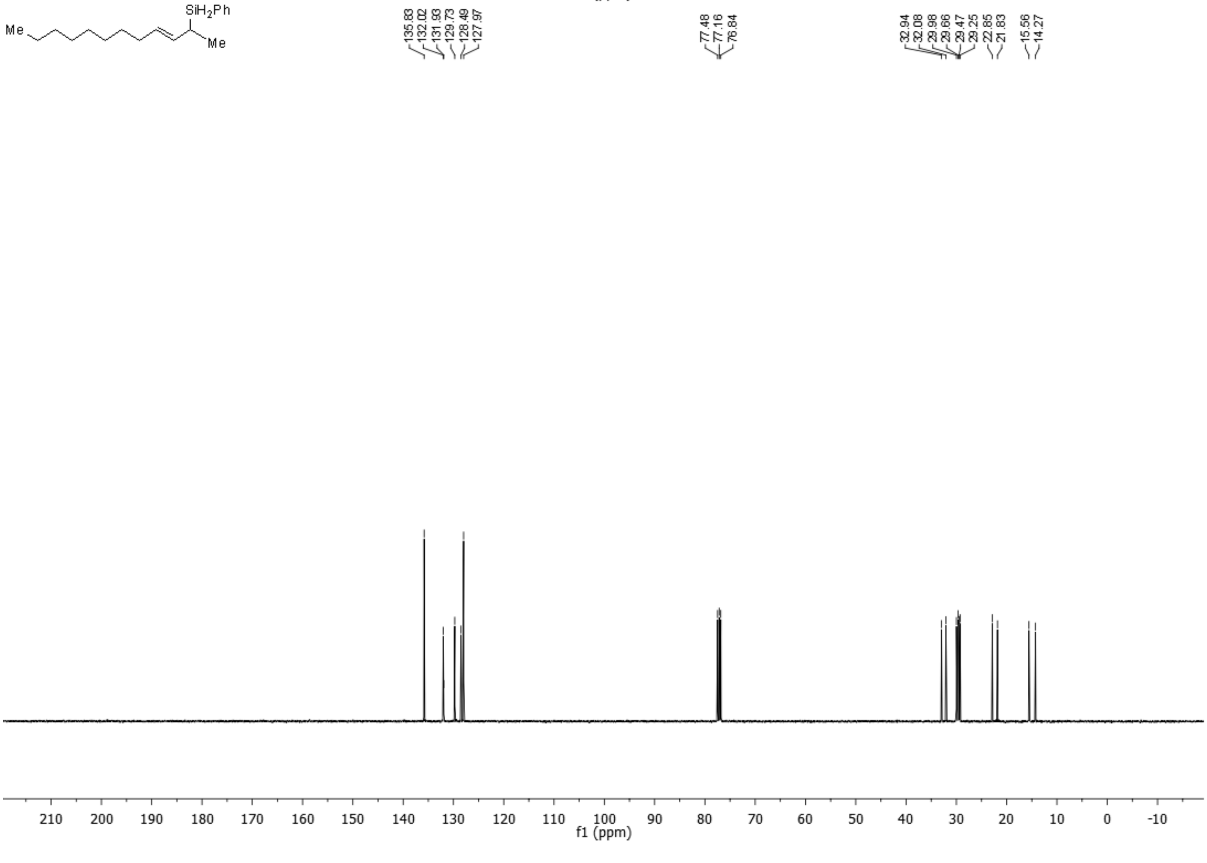
SiH2Ph
Me
Me
135.83
132.02
131.93
129.73
128.49
127.97
77.48
77.16
76.84
32.94
32.08
29.98
29.66
29.47
29.25
22.85
21.83
15.56
14.27
f1 (ppm)

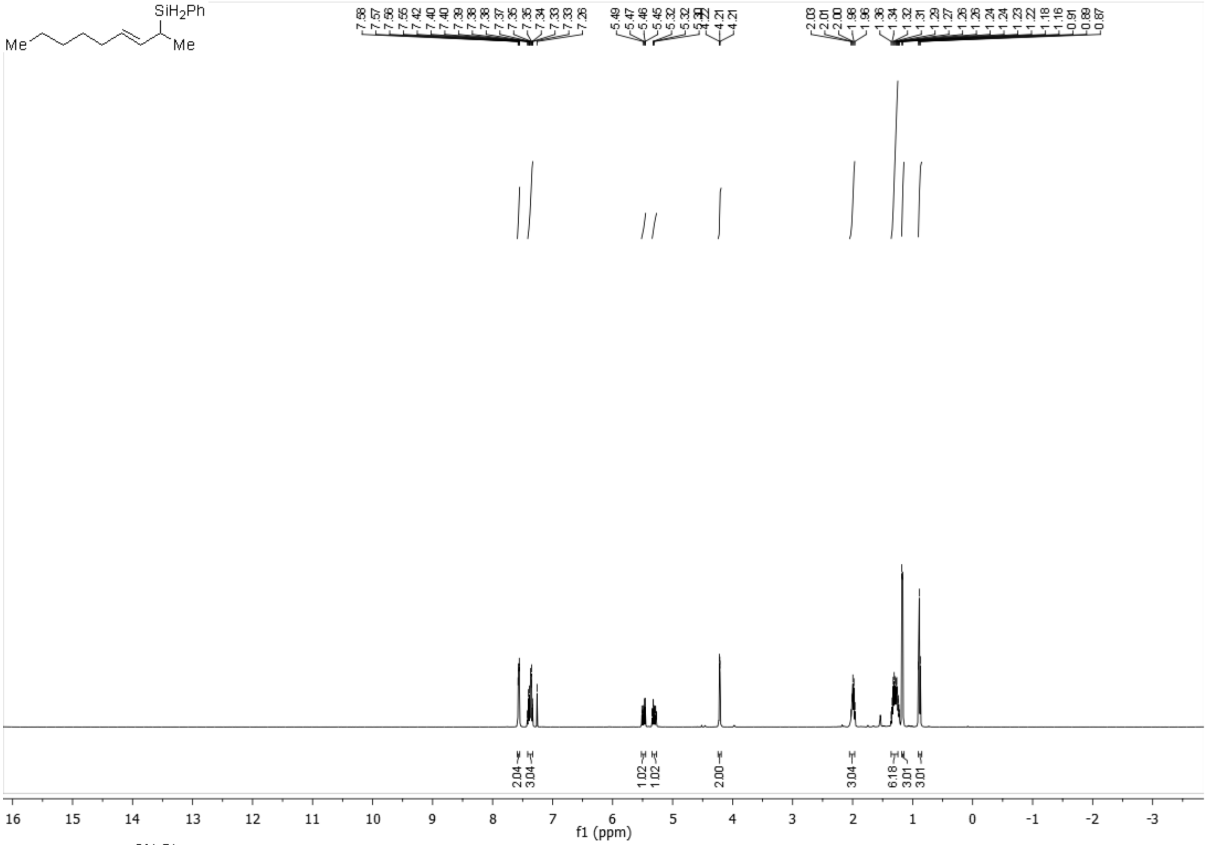

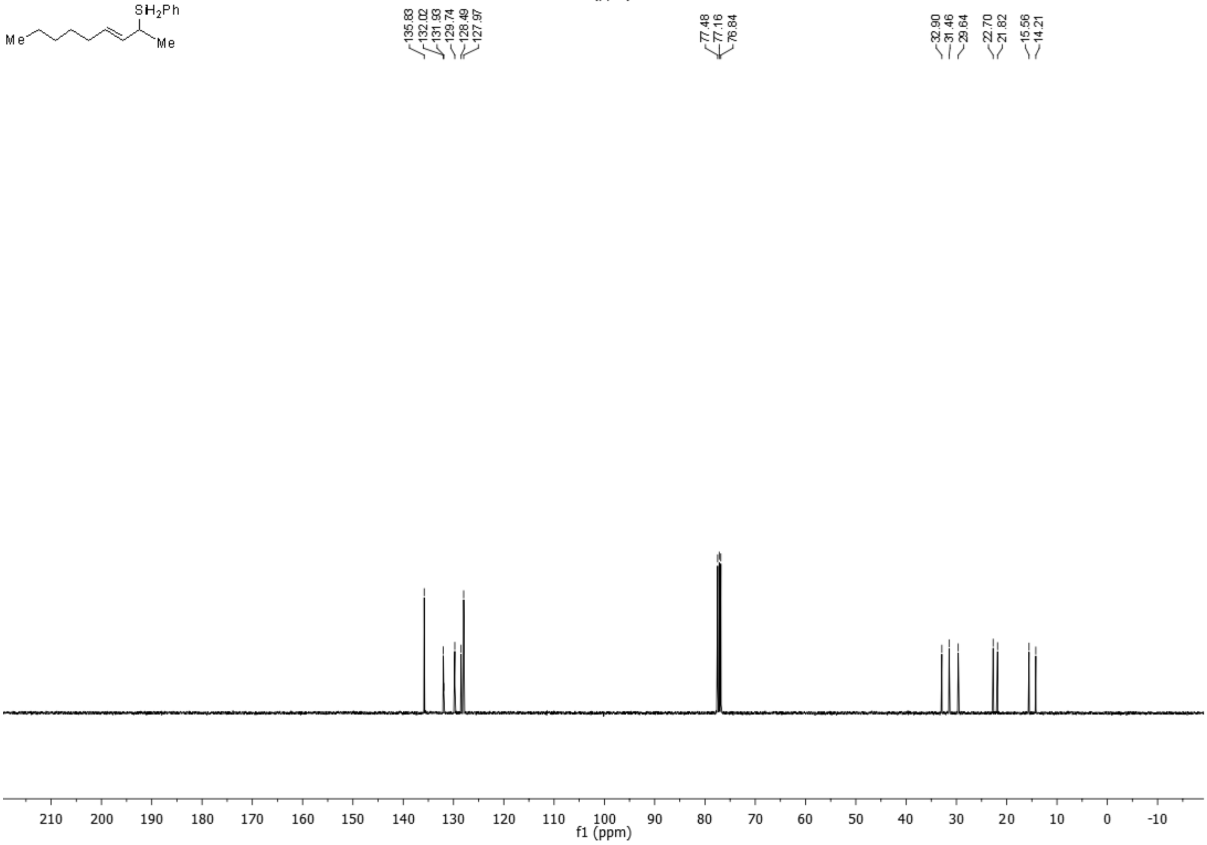

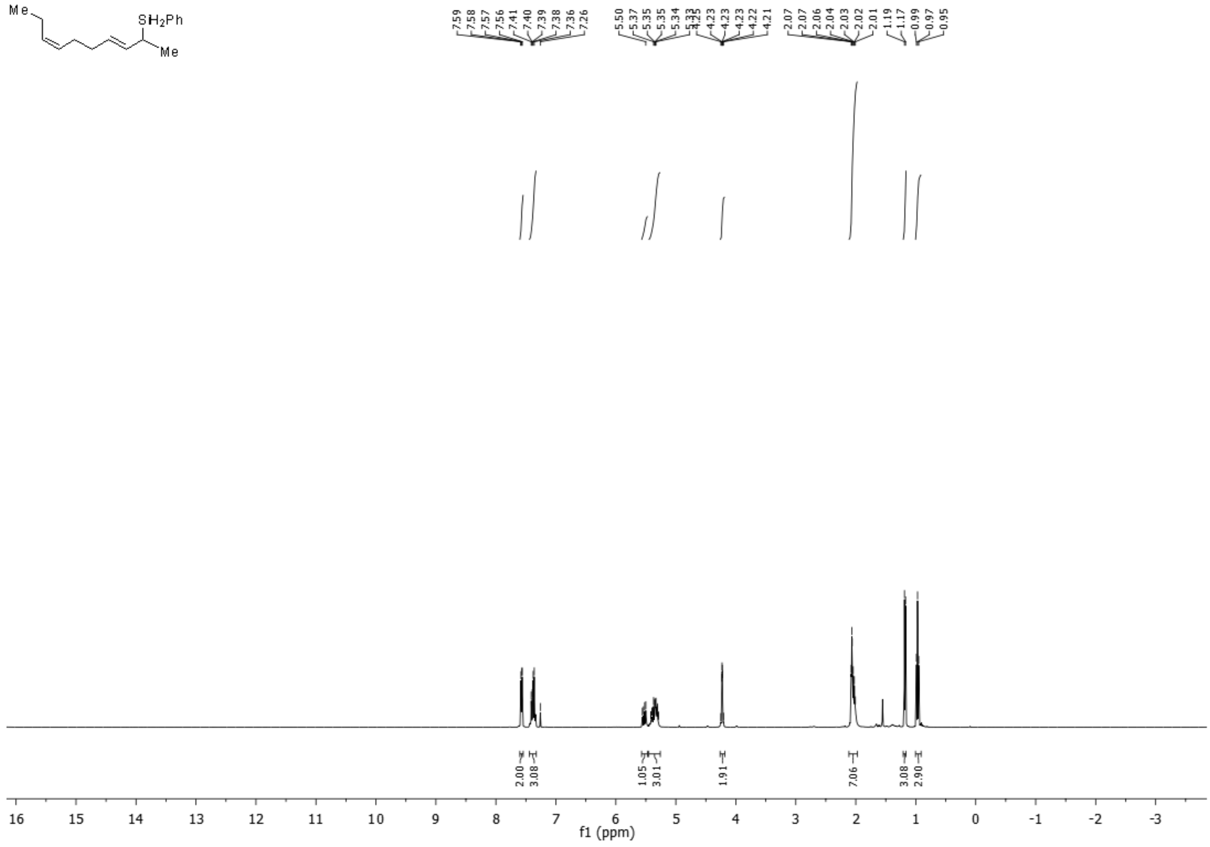
Me
SH2Ph
Me
7.59 7.58 7.57 7.56 7.41 7.40 7.39 7.38 7.36 7.26
5.50 5.37 5.35 5.35 5.34 5.33 4.25 4.23 4.23 4.23 4.22 4.21
2.07 2.07 2.06 2.04 2.03 2.02 2.01 1.19 1.17 0.99 0.97 0.95
2.00 3.08 1.05 3.01 1.91 7.06 3.08 2.90
f1 (ppm)

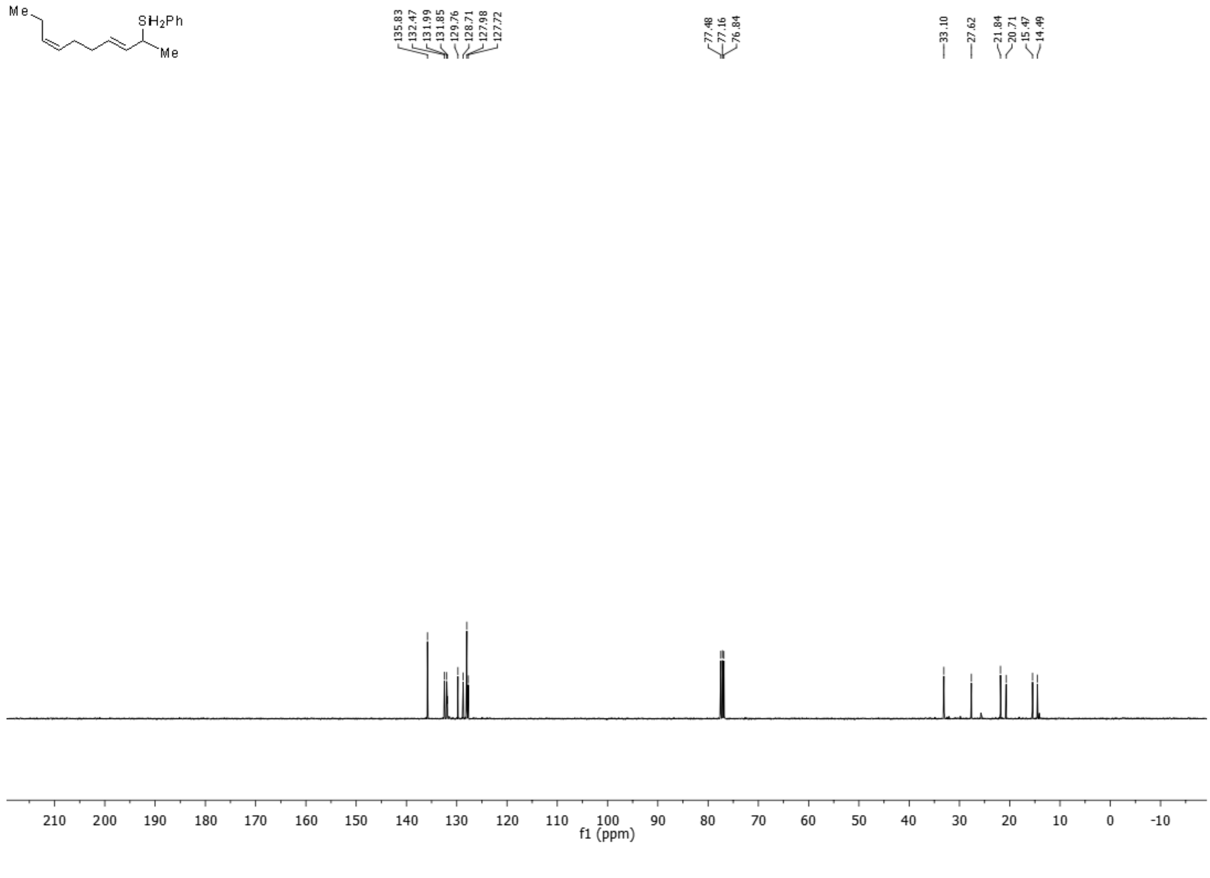
Me
SH2Ph
Me
135.83 132.47 131.99 131.85 129.76 128.71 127.98 127.72
77.48 77.16 76.84
33.10 27.62 21.84 20.71 15.47 14.49
f1 (ppm)

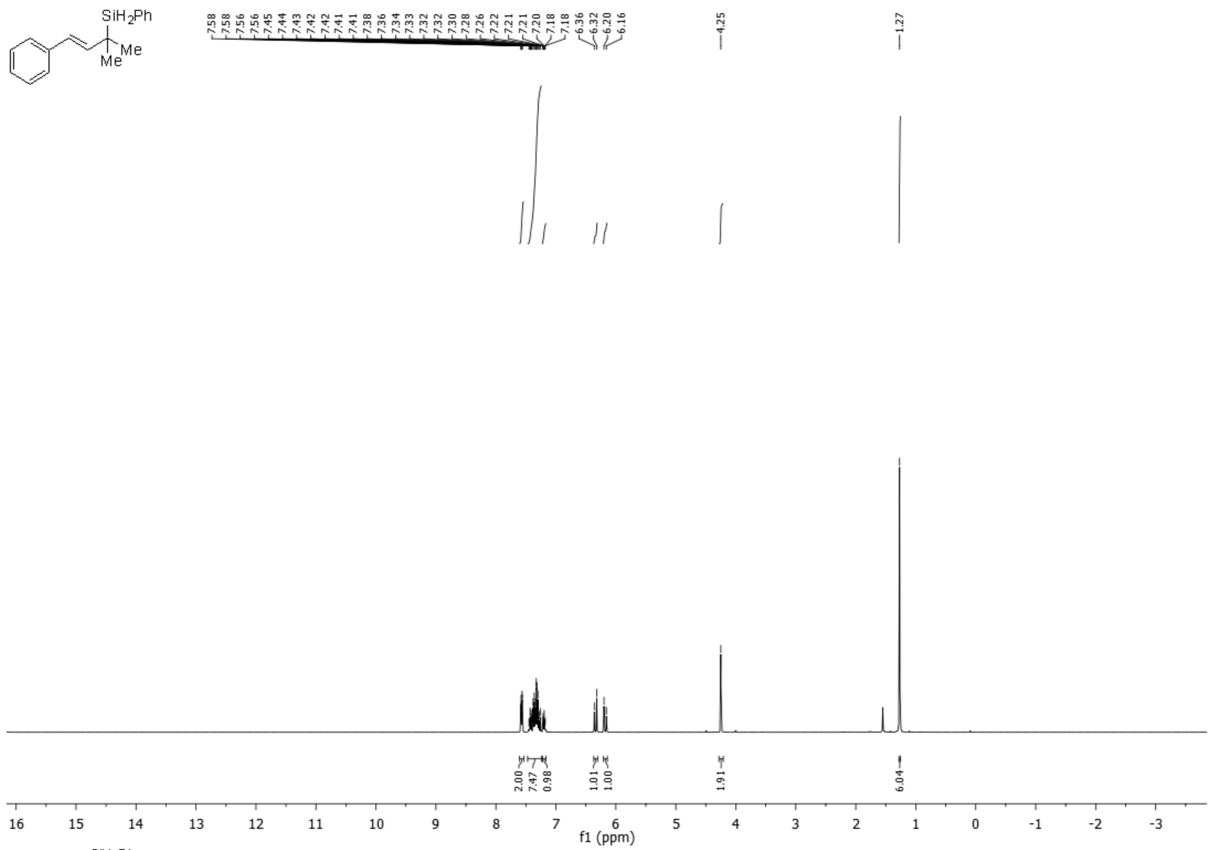
SiH2Ph
Me
Me
7.58 7.58 7.56 7.56 7.45 7.44 7.43 7.42 7.42 7.41 7.41 7.38 7.36 7.34 7.33 7.32 7.32 7.30 7.28 7.26 7.22 7.21 7.21 7.20 7.18 6.36 6.32 6.20 6.16
4.25
1.27
2.00 7.47 0.98 1.01 1.00 1.91 6.04
f1 (ppm)

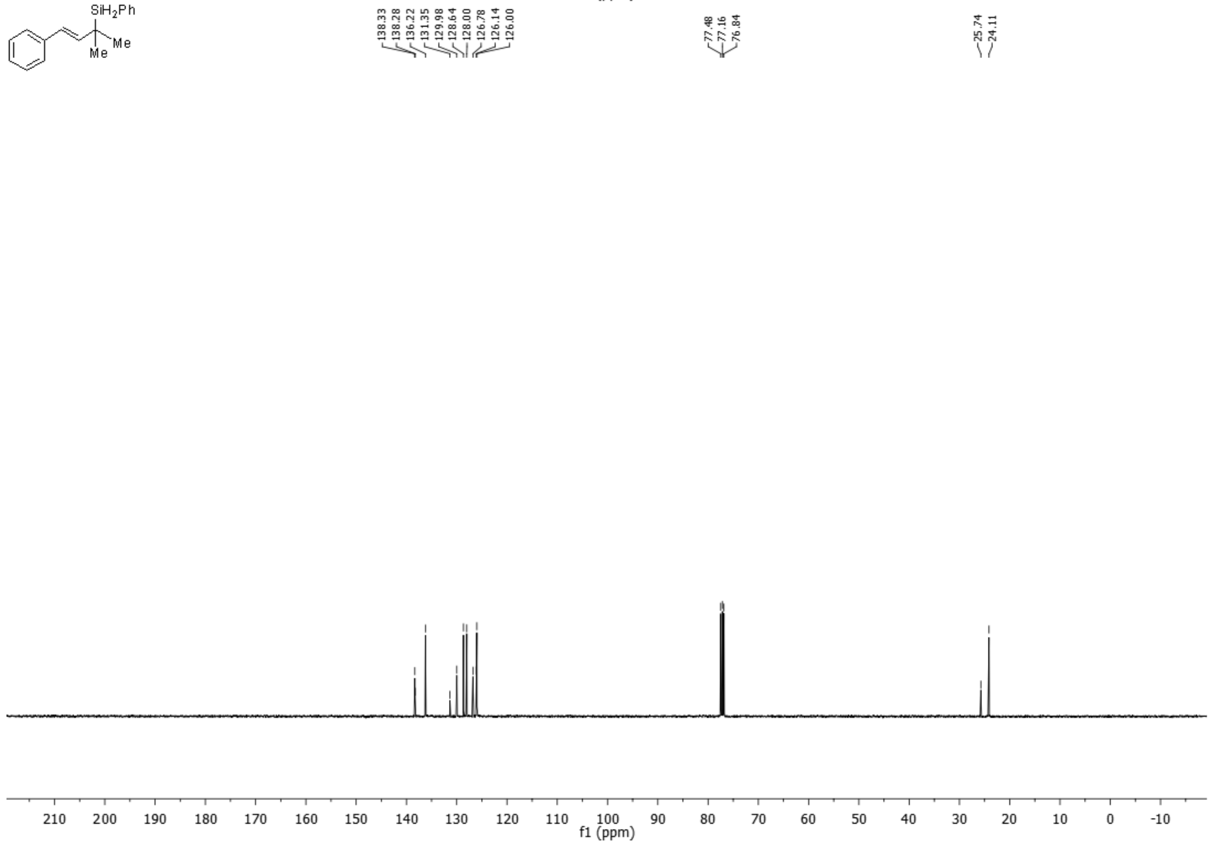
SiH2Ph
Me
Me
138.33 138.28 136.22 131.35 129.98 128.64 128.00 126.78 126.14 126.00
77.48 77.16 76.84
25.74 24.11
f1 (ppm)

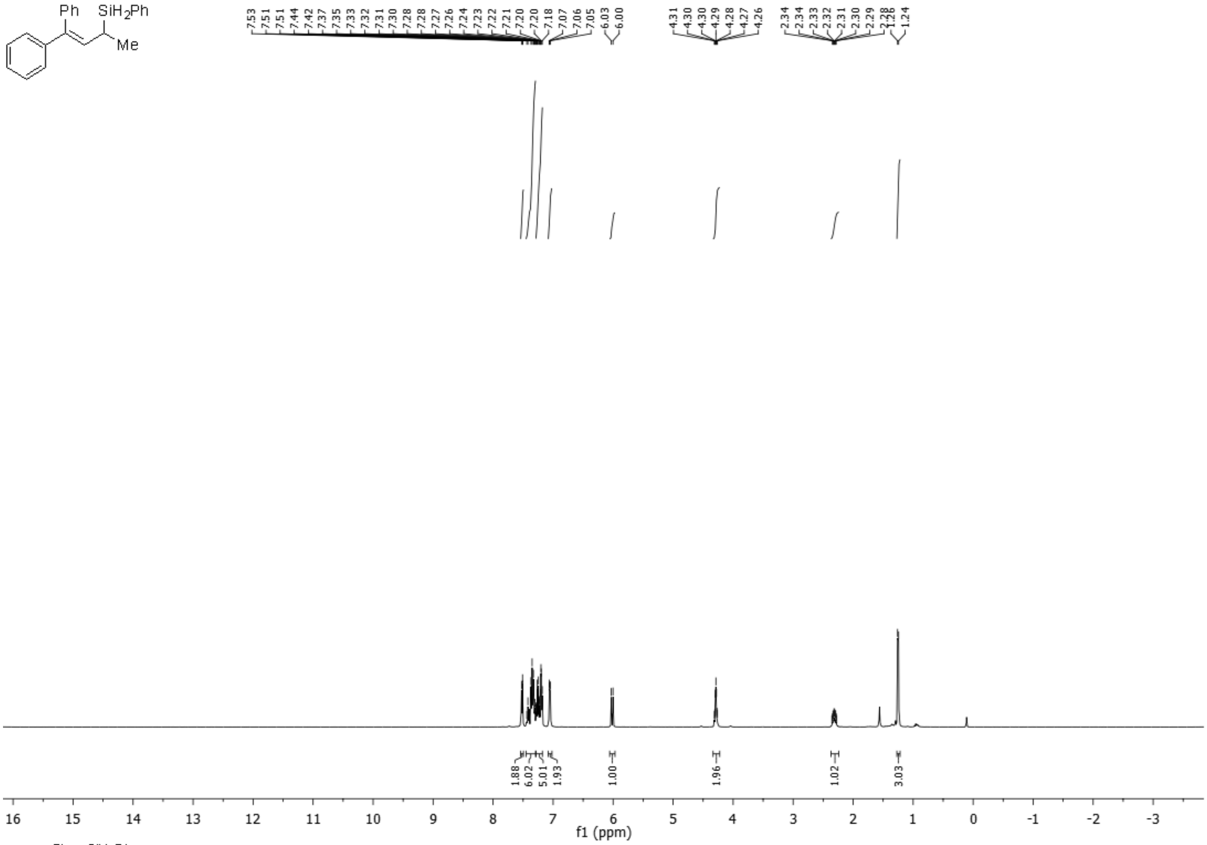
Ph SiH2Ph Me
7.53 7.51 7.51 7.44 7.42 7.37 7.35 7.33 7.32 7.31 7.30 7.28 7.28 7.27 7.26 7.24 7.23 7.22 7.21 7.20 7.20 7.18 7.07 7.06 7.05 6.03 6.00
4.31 4.30 4.30 4.29 4.28 4.27 4.26
2.34 2.34 2.33 2.32 2.31 2.30 2.29 1.28 1.26 1.24
1.88 6.02 5.01 1.93 1.00 1.96 1.02 3.03
16 15 14 13 12 11 10 9 8 7 6 5 4 3 2 1 0 -1 -2 -3
f1 (ppm)

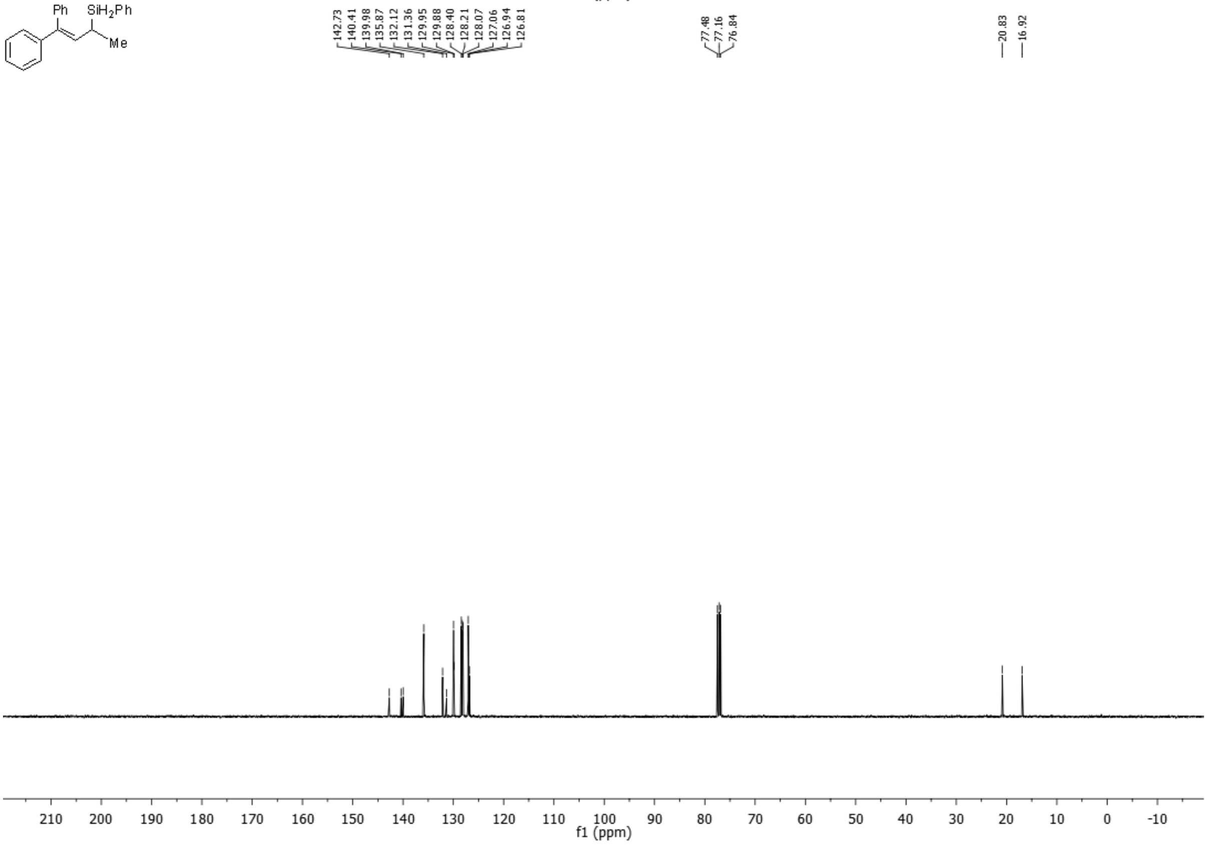
Ph SiH2Ph Me
142.73 140.41 139.98 135.87 132.12 131.36 129.95 129.88 128.40 128.21 128.07 127.06 126.94 126.81
77.48 77.16 76.84
20.83 16.92
210 200 190 180 170 160 150 140 130 120 110 100 90 80 70 60 50 40 30 20 10 0 -10
f1 (ppm)

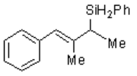
SiH2Ph
Me
Me

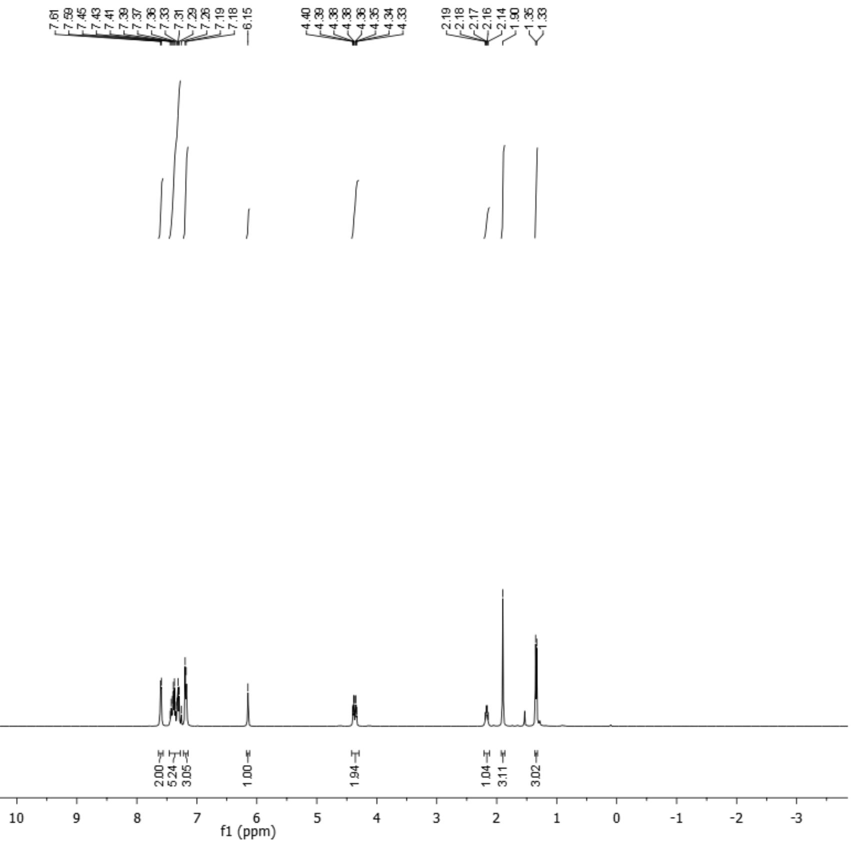
7.61 7.59 7.45 7.43 7.41 7.39 7.37 7.36 7.33 7.31 7.29 7.26 7.19 7.18 6.15
4.40 4.39 4.38 4.38 4.36 4.35 4.34 4.33
2.19 2.18 2.17 2.16 2.14 1.90 1.35 1.33
2.00 5.24 3.05 1.00 1.94 1.04 3.11 3.02
f1 (ppm)

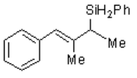
SiH2Ph
Me
Me

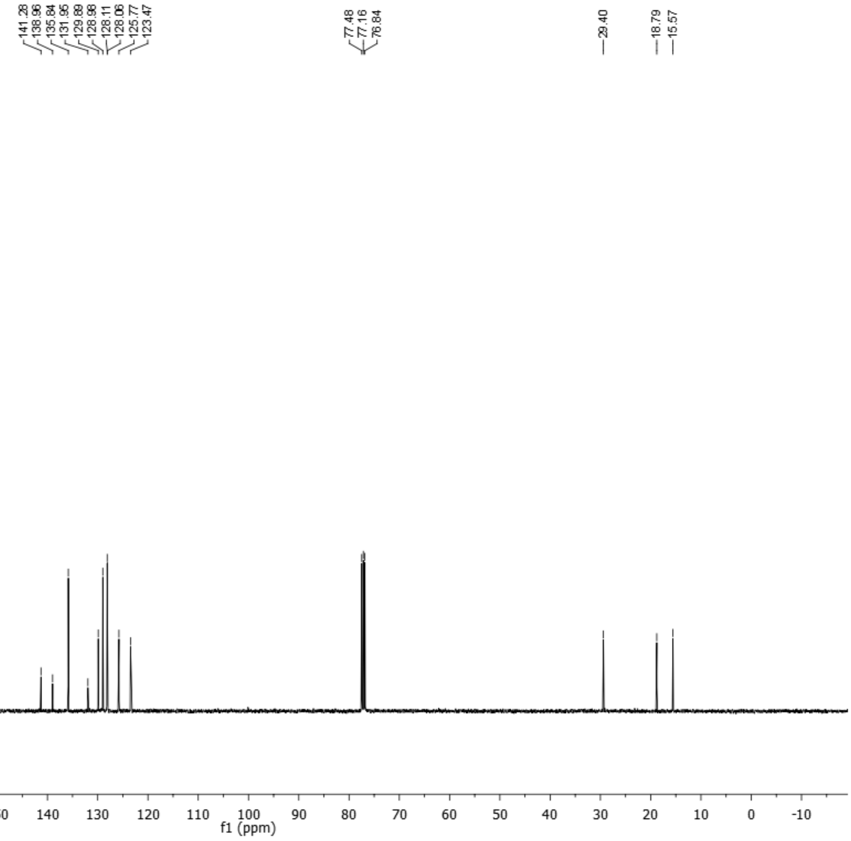
141.28 138.96 135.84 131.95 129.89 128.98 128.11 128.06 125.77 123.47
77.48 77.16 76.84
29.40
18.79 15.57
f1 (ppm)

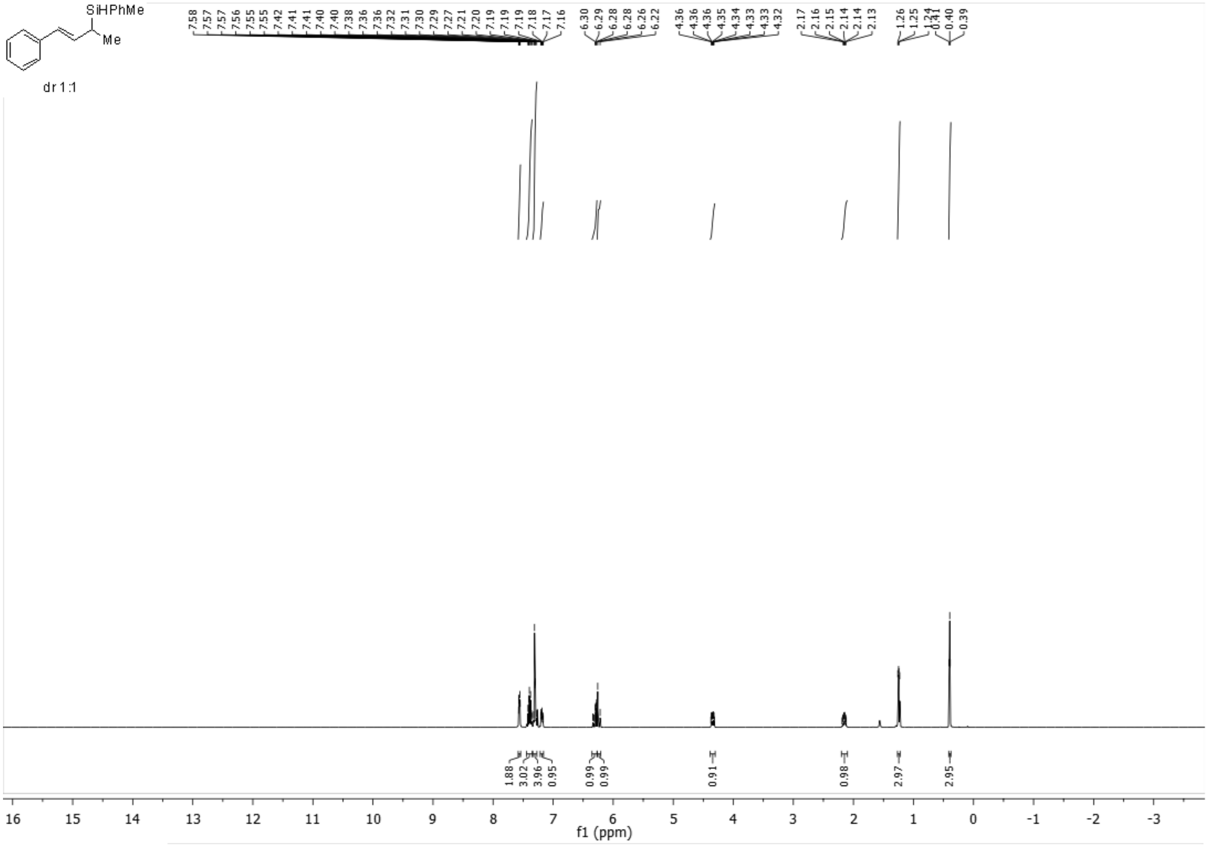
SiHPhMe
Me
dr 1:1
7.58 7.57 7.57 7.56 7.55 7.55 7.42 7.41 7.41 7.40 7.38 7.36 7.36 7.32 7.31 7.30 7.29 7.27 7.21 7.20 7.19 7.19 7.18 7.17 7.16
6.30 6.29 6.28 6.28 6.26 6.22
4.36 4.36 4.36 4.35 4.34 4.33 4.33 4.32
2.17 2.16 2.15 2.14 2.14 2.13
1.26 1.25 1.24 0.41 0.40 0.39
1.88 3.02 3.96 0.95 0.99 0.99 0.91 0.98 2.97 2.95
f1 (ppm)

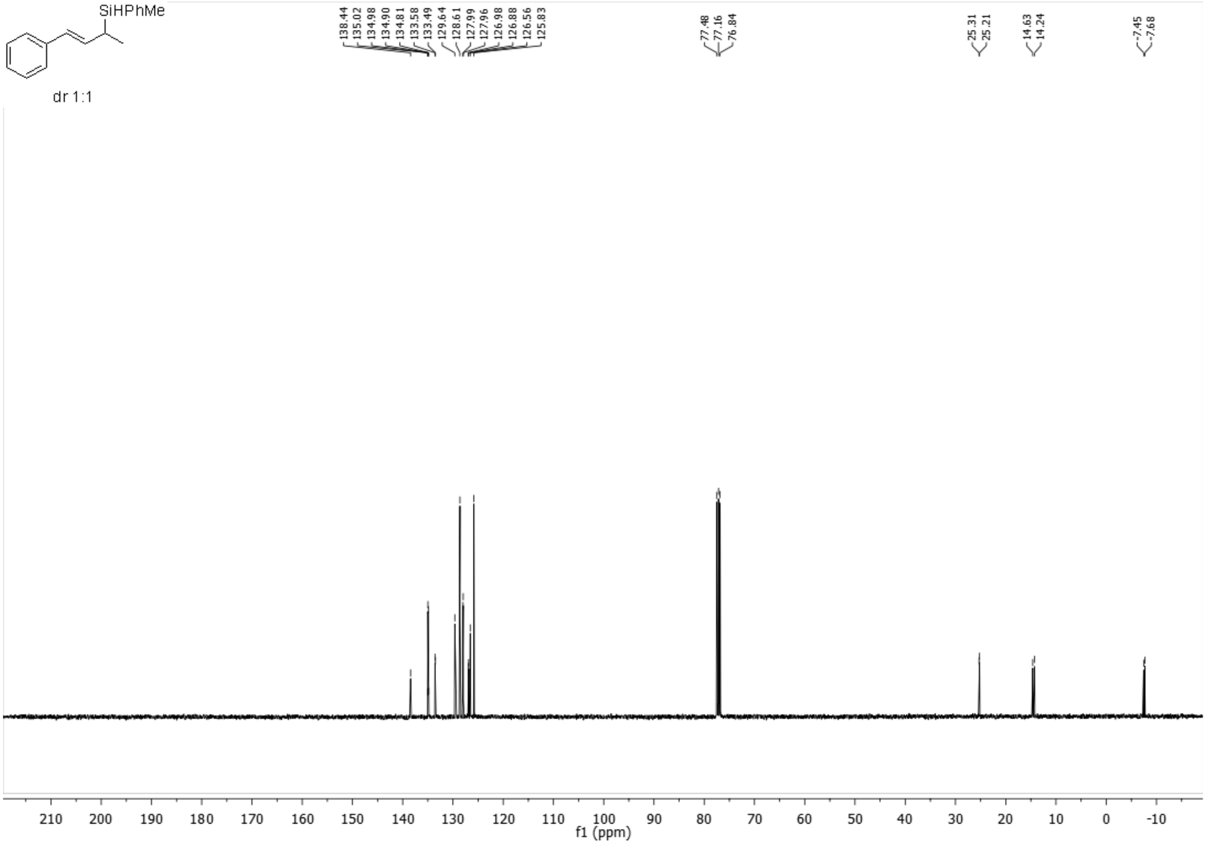
SiHPhMe
dr 1:1
138.44 135.02 134.98 134.90 134.81 133.58 133.49 129.64 128.61 127.99 127.96 126.98 126.88 126.56 125.83
77.48 77.16 76.84
25.31 25.21
14.63 14.24
-7.45 -7.68
f1 (ppm)

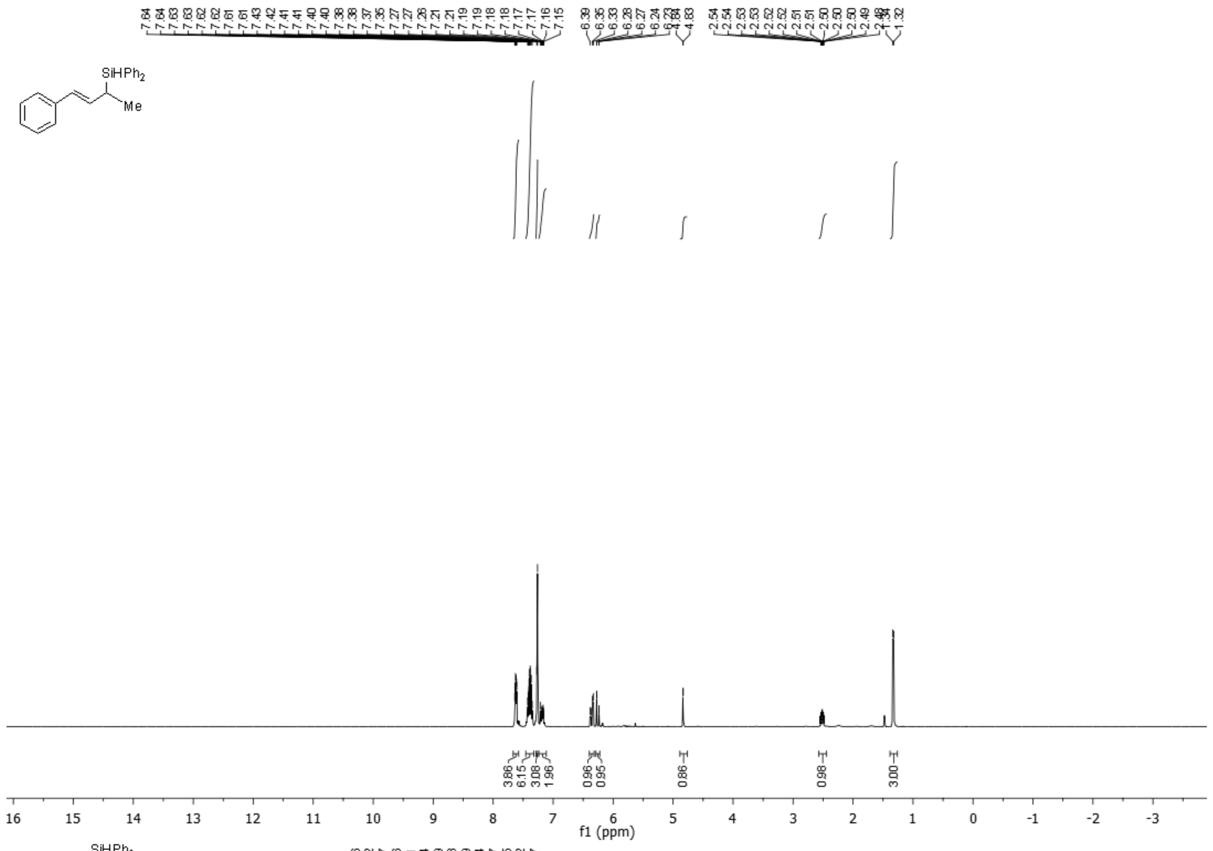
SiHPh2
Me
f1 (ppm)

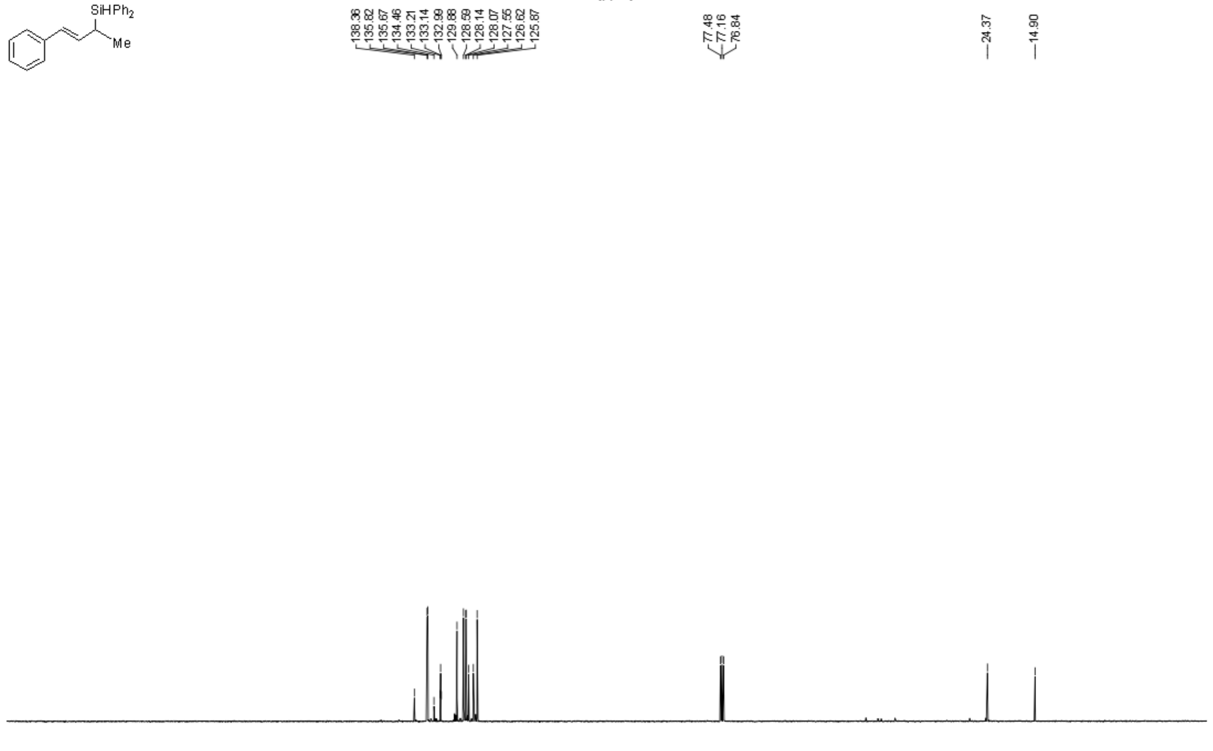
SiHPh2
Me
138.36
135.82
135.67
134.46
133.21
133.14
132.99
129.88
128.59
128.14
128.07
127.55
126.62
125.87
77.48
77.16
76.84
24.37
14.90
f1 (ppm)

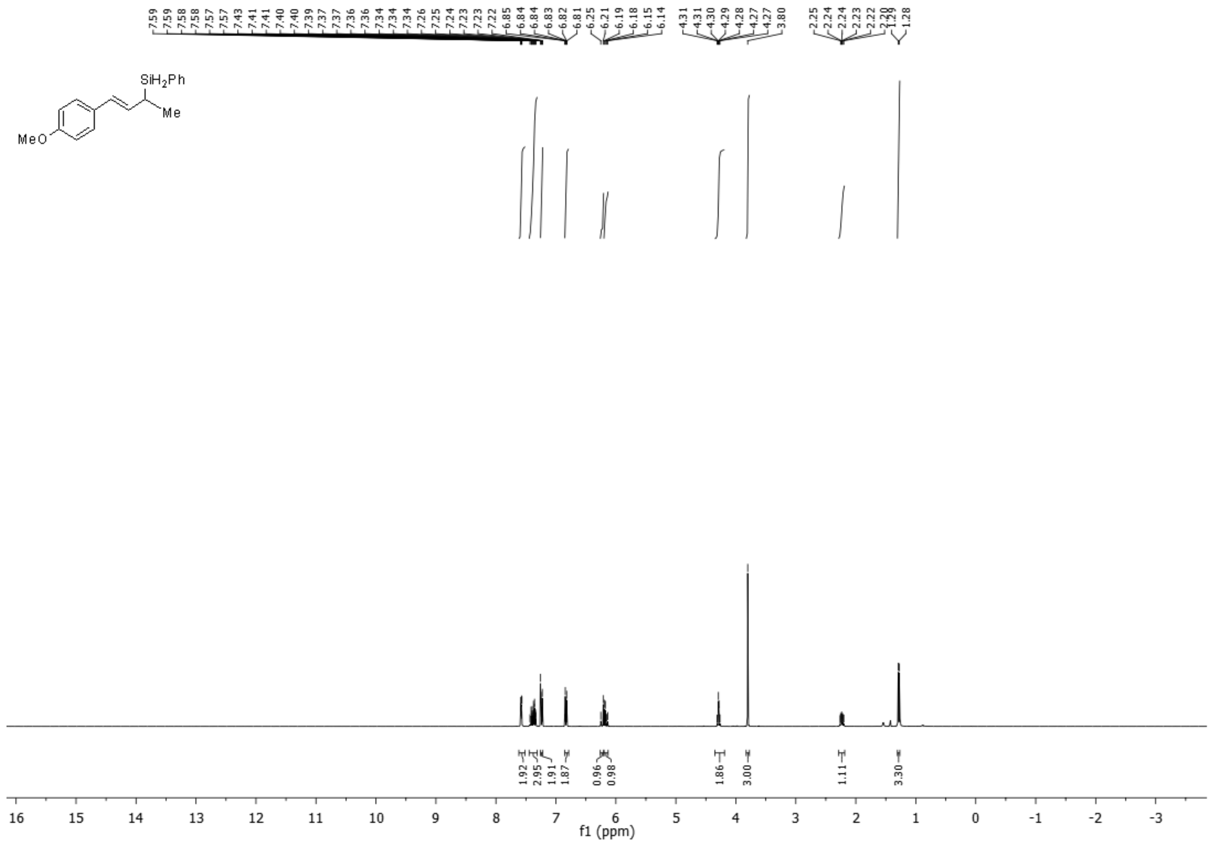
SiH2Ph
Me
MeO
7.59 7.59 7.58 7.58 7.57 7.43 7.41 7.41 7.40 7.39 7.37 7.37 7.36 7.36 7.34 7.34 7.34 7.34 7.26 7.25 7.24 7.23 7.23 7.22 6.85 6.84 6.84 6.83 6.82 6.81 6.25 6.21 6.19 6.18 6.15 6.14 4.31 4.31 4.30 4.29 4.28 4.27 4.27 3.80 2.25 2.24 2.24 2.23 2.22 2.20 1.29 1.28
1.92 2.95 1.91 1.87 0.96 0.98 1.86 3.00 1.11 3.30
f1 (ppm)

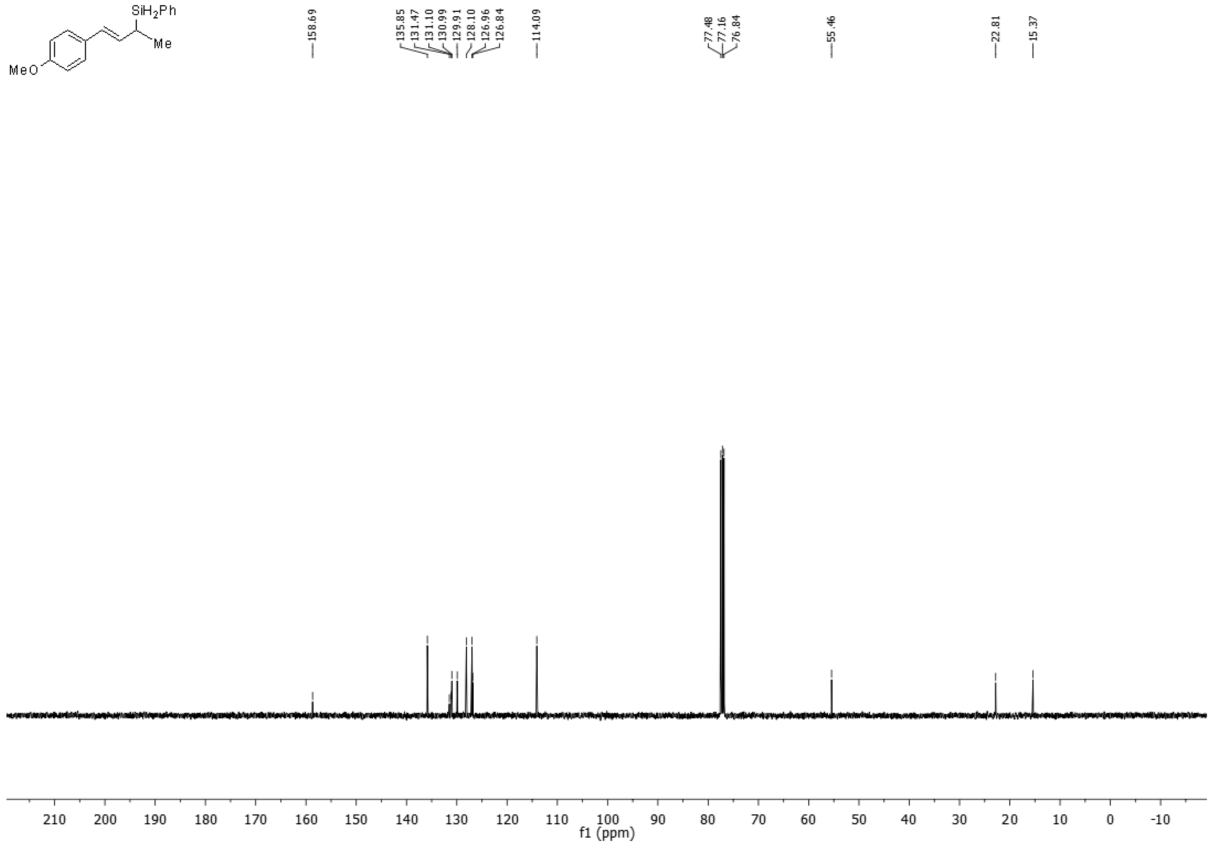
SiH2Ph
Me
MeO
158.69 135.85 131.47 131.10 130.99 129.91 128.10 126.96 126.84 114.09 77.48 77.16 76.84 55.46 22.81 15.37
f1 (ppm)

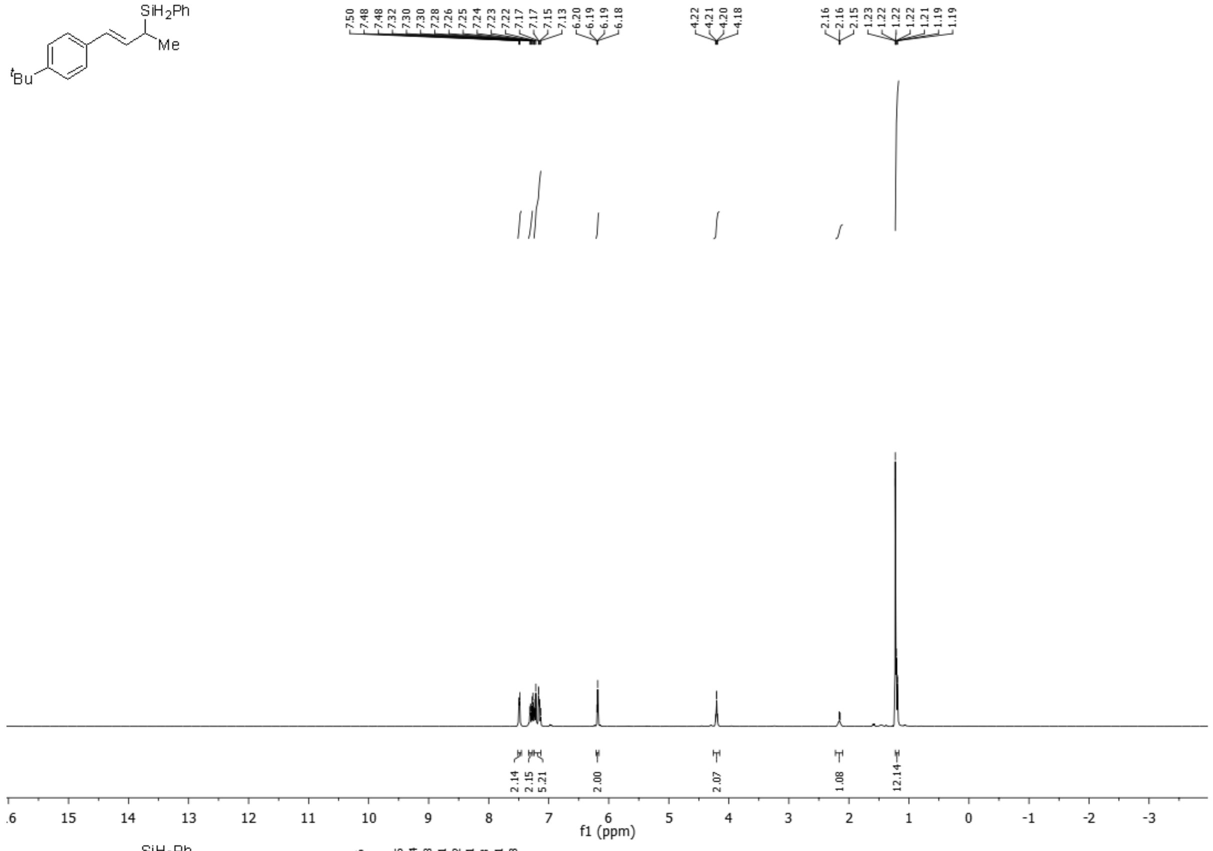

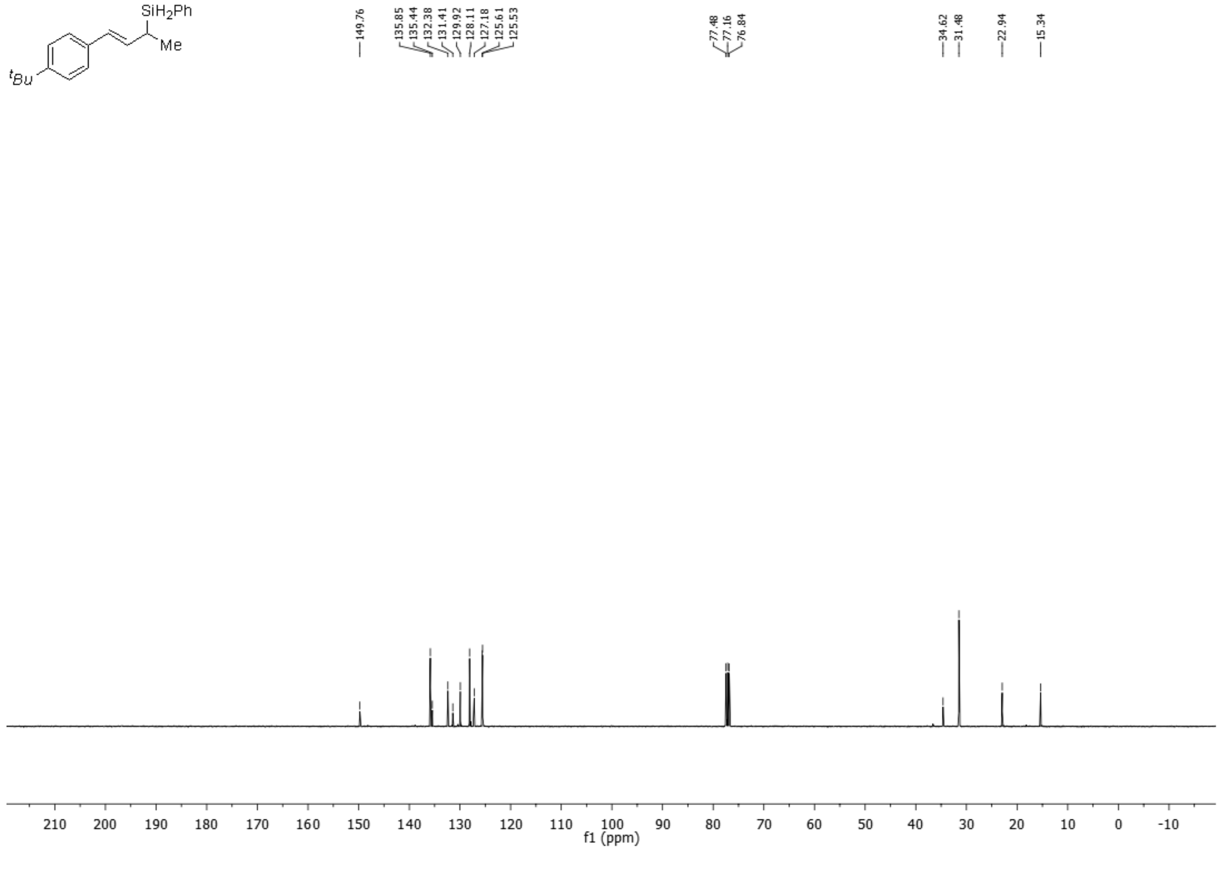

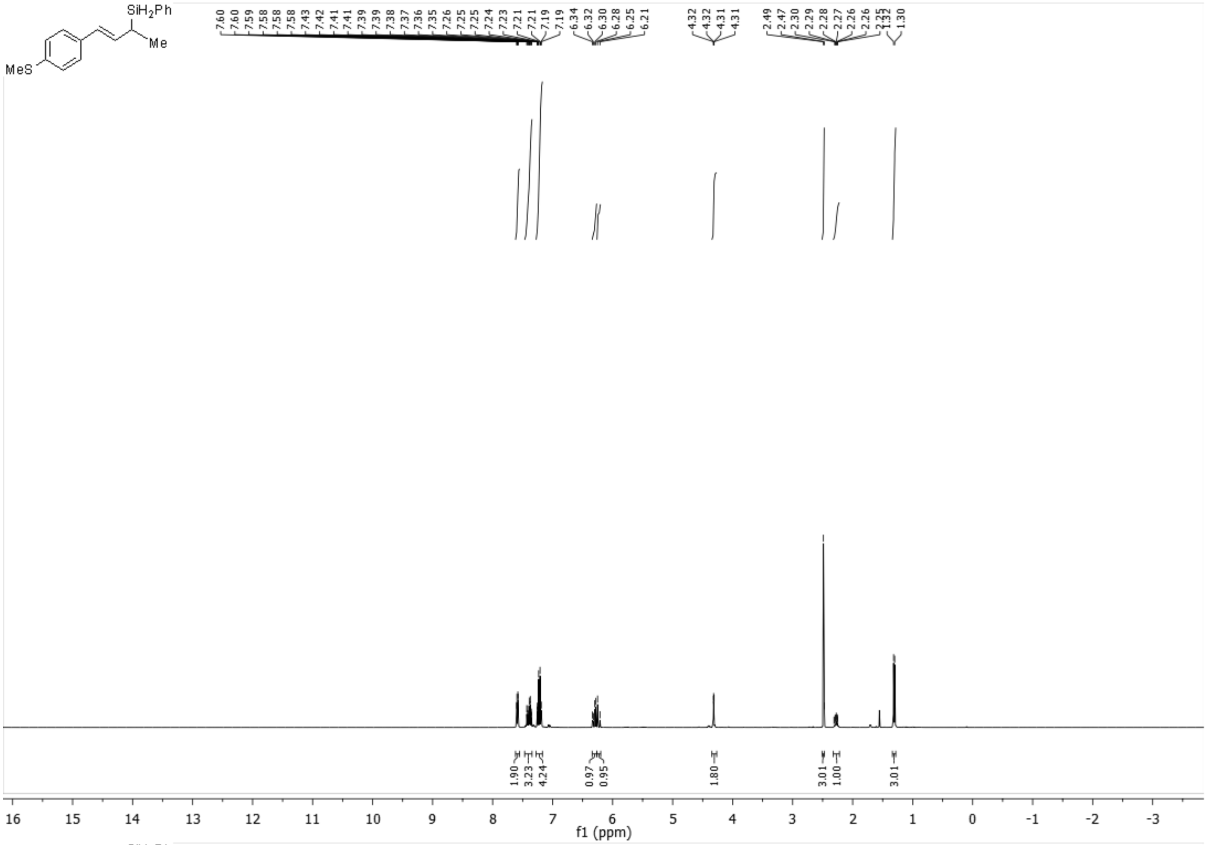
SiH2Ph
Me
MeS
7.60 7.60 7.59 7.58 7.58 7.58 7.43 7.42 7.41 7.41 7.39 7.39 7.38 7.37 7.36 7.35 7.26 7.25 7.25 7.24 7.23 7.21 7.21 7.19 7.19 6.34 6.32 6.30 6.28 6.25 6.21
4.32 4.32 4.31 4.31
2.49 2.47 2.30 2.29 2.28 2.27 2.26 2.26 2.25 1.32 1.30
1.90 3.23 4.24 0.97 0.95 1.80 3.01 1.00 3.01
f1 (ppm)

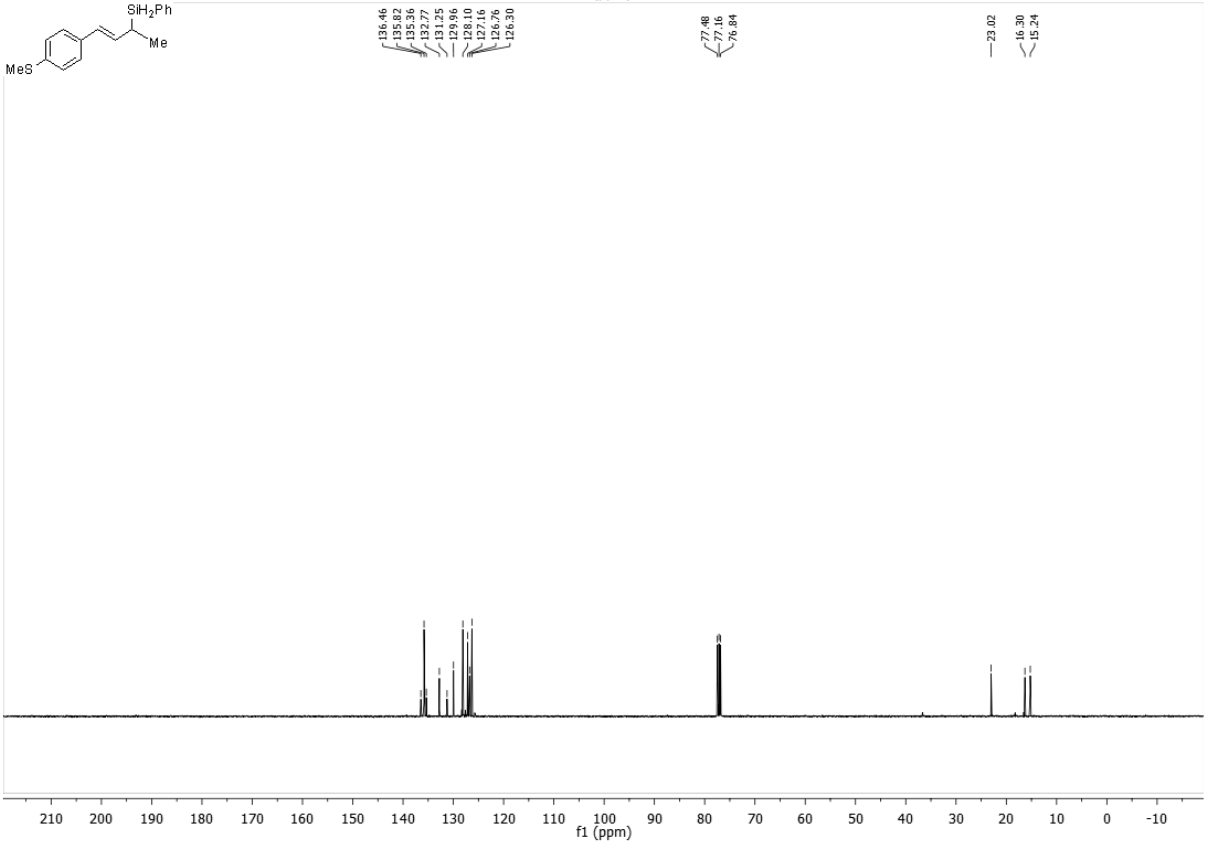
SiH2Ph
Me
MeS
136.46 135.82 135.36 132.77 131.25 129.96 128.10 127.16 126.76 126.30
77.48 77.16 76.84
23.02
16.30 15.24
f1 (ppm)

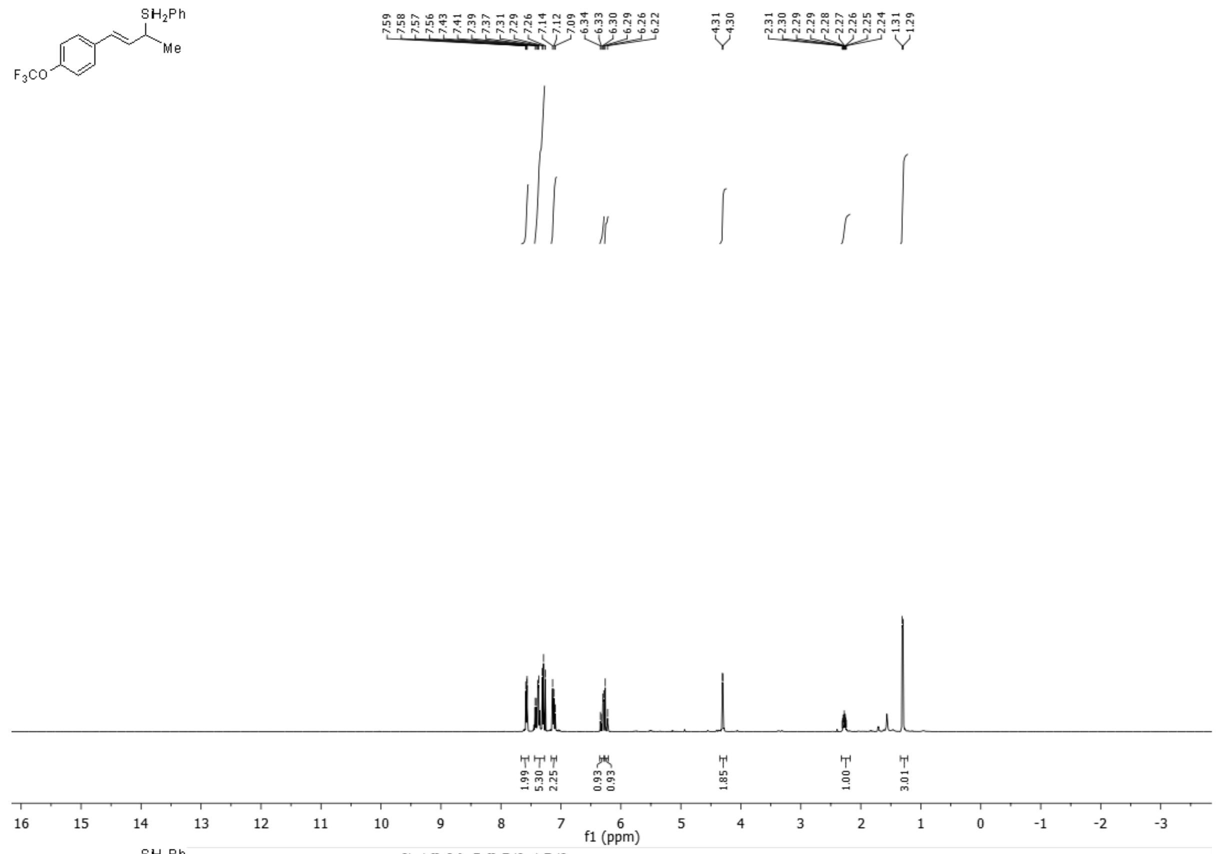

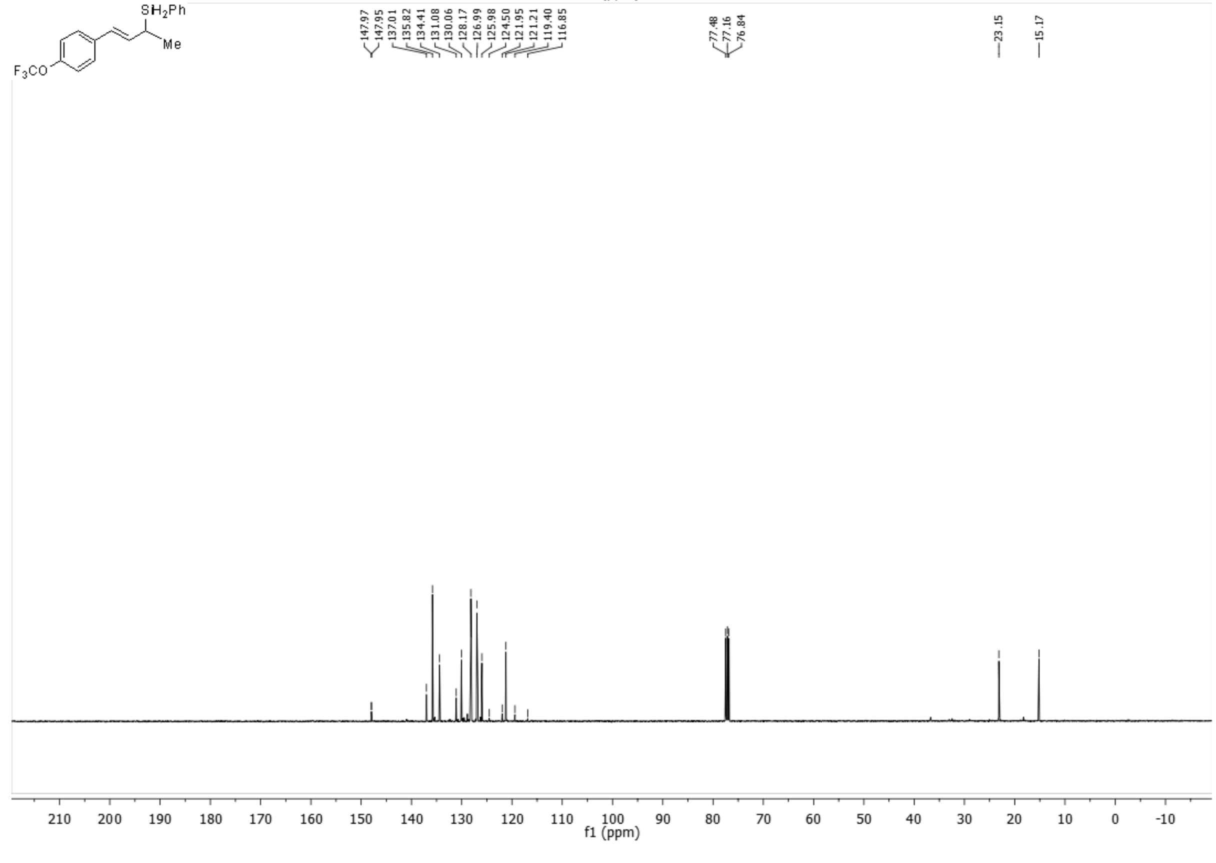

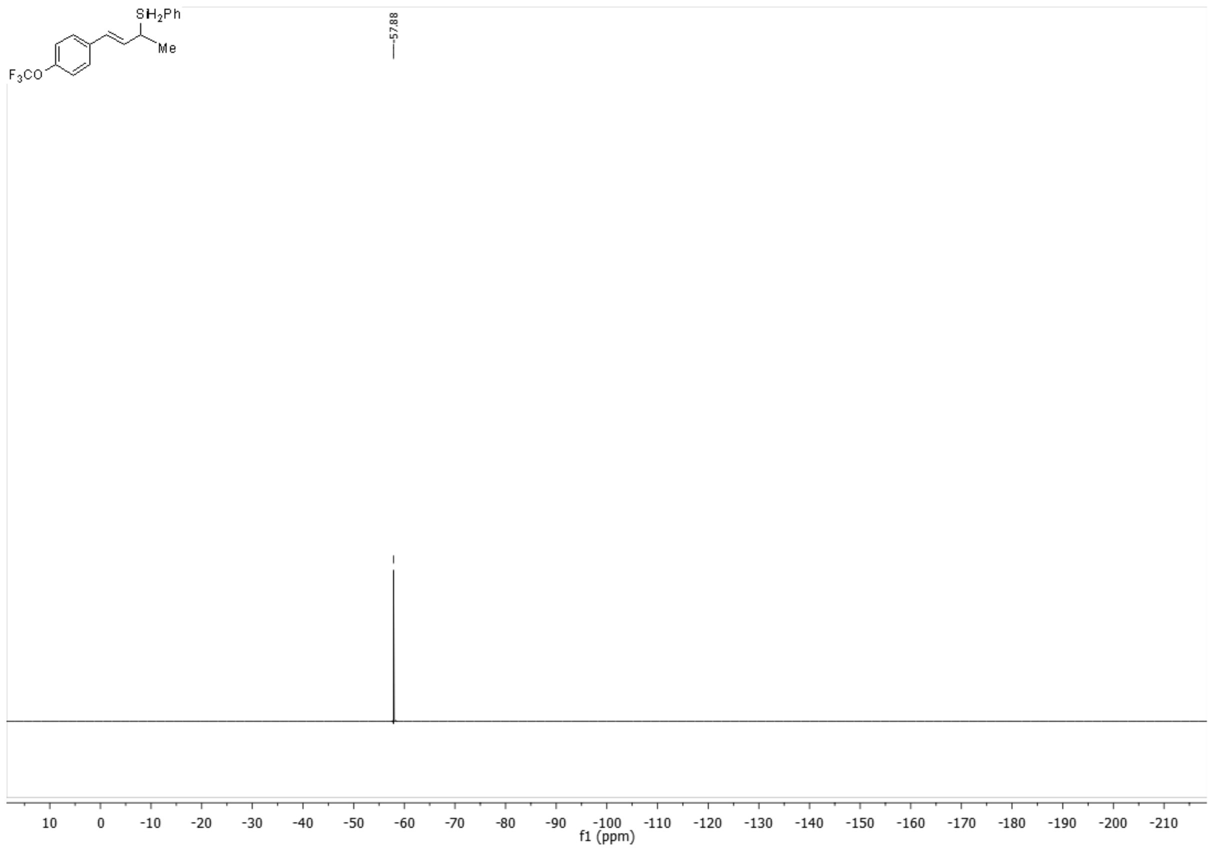
SiH2Ph
Me
F3CO
—57.88
10 0 -10 -20 -30 -40 -50 -60 -70 -80 -90 -100 -110 -120 -130 -140 -150 -160 -170 -180 -190 -200 -210
f1 (ppm)

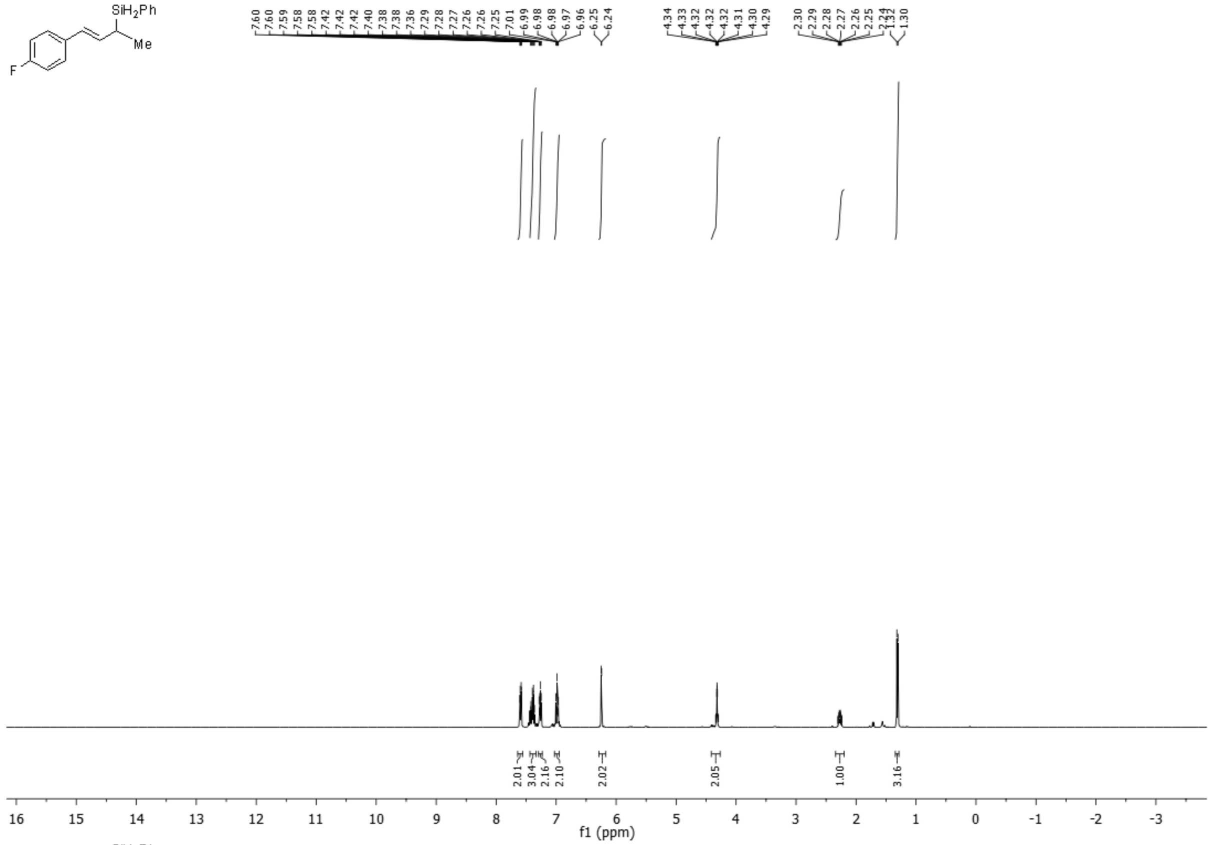
SiH2Ph
Me
F
f1 (ppm)

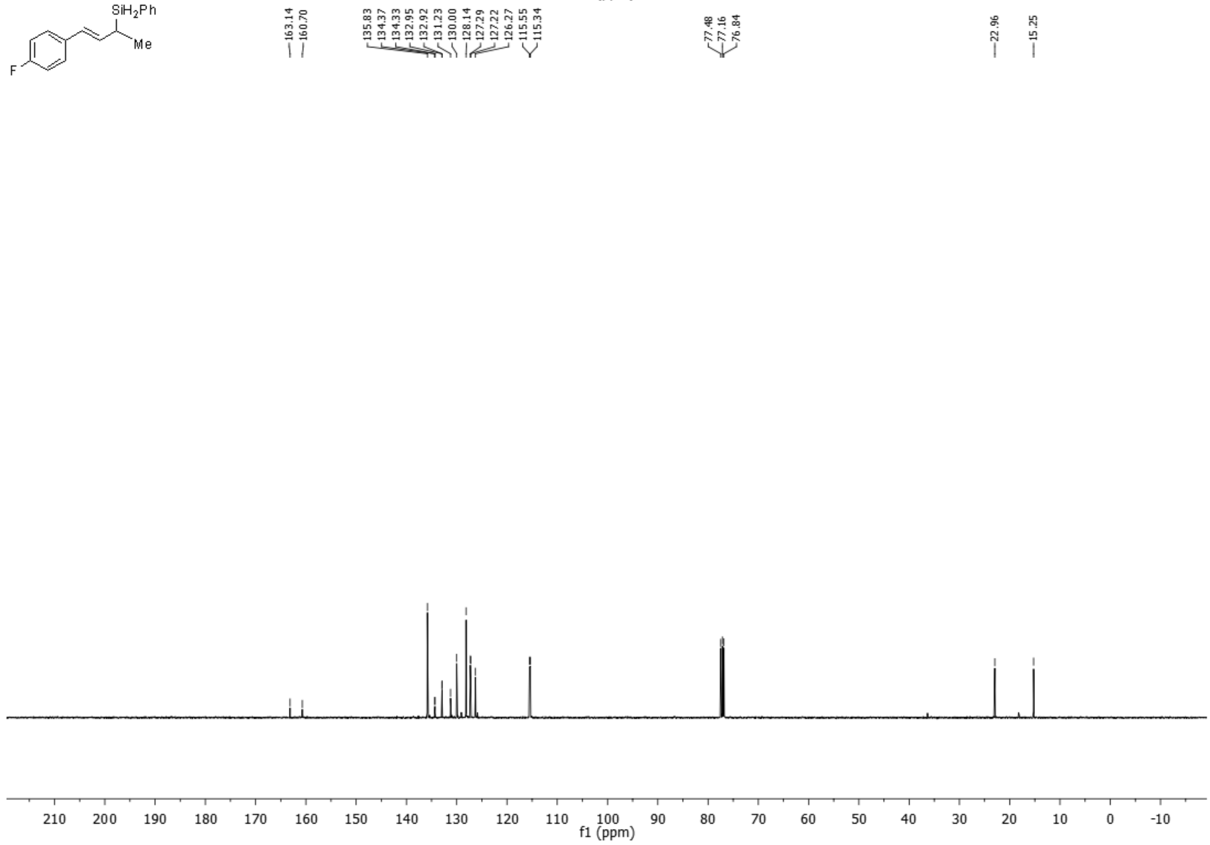
SiH2Ph
Me
F
f1 (ppm)

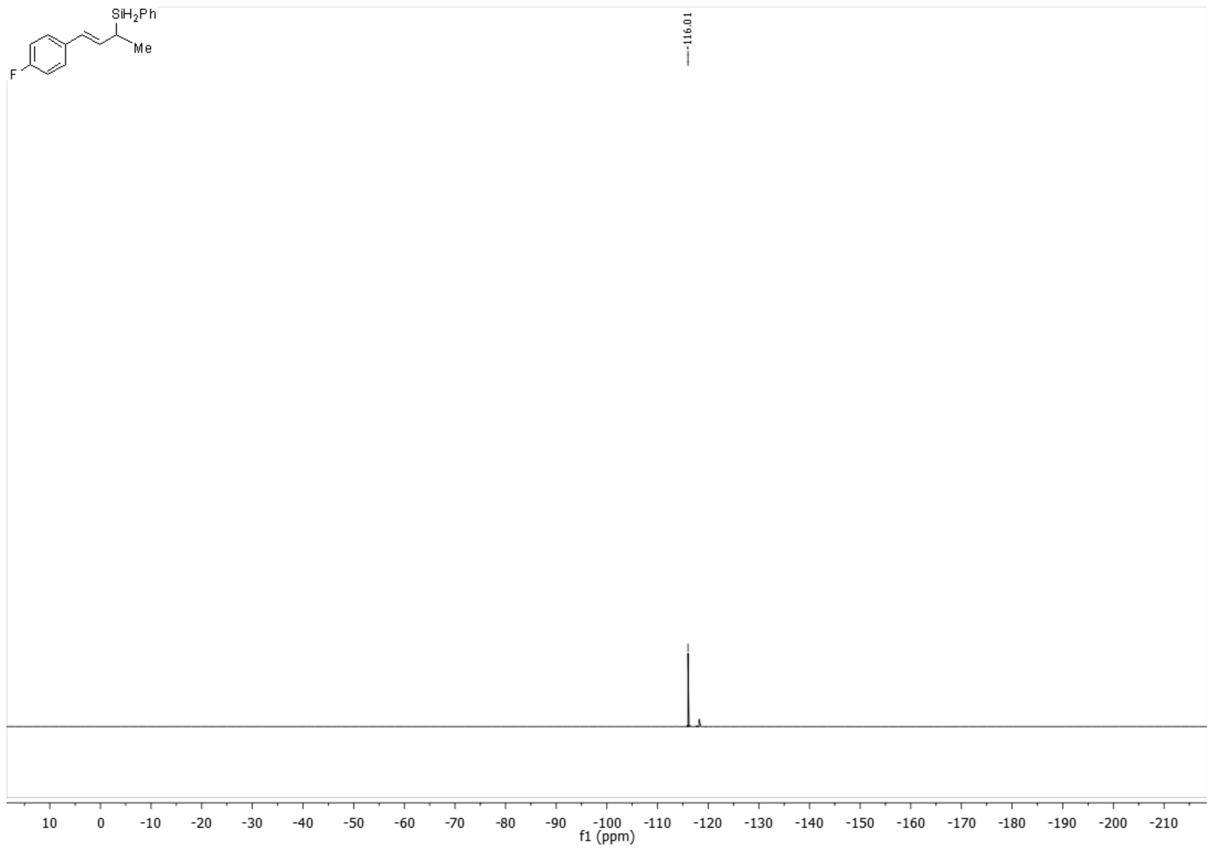
SiH2Ph
Me
F
-116.01
10 0 -10 -20 -30 -40 -50 -60 -70 -80 -90 -100 -110 -120 -130 -140 -150 -160 -170 -180 -190 -200 -210
f1 (ppm)

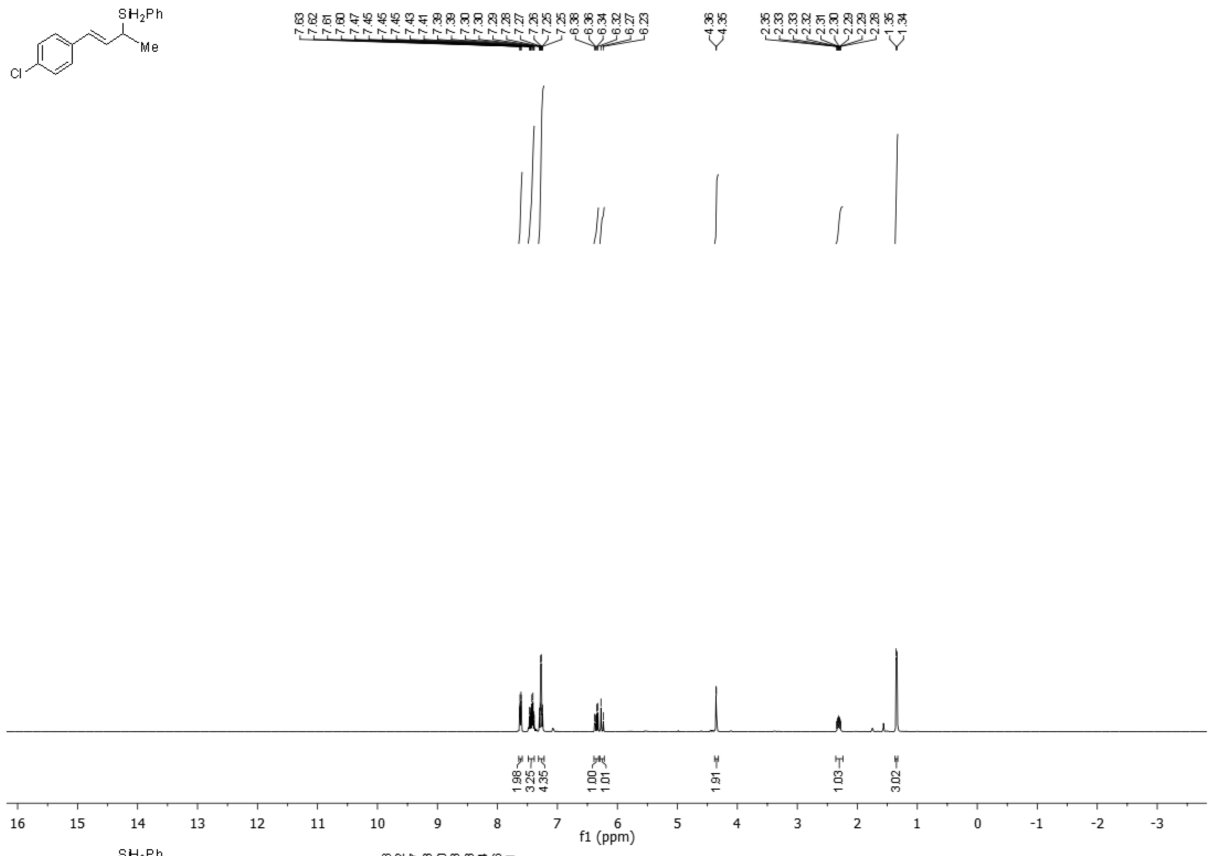

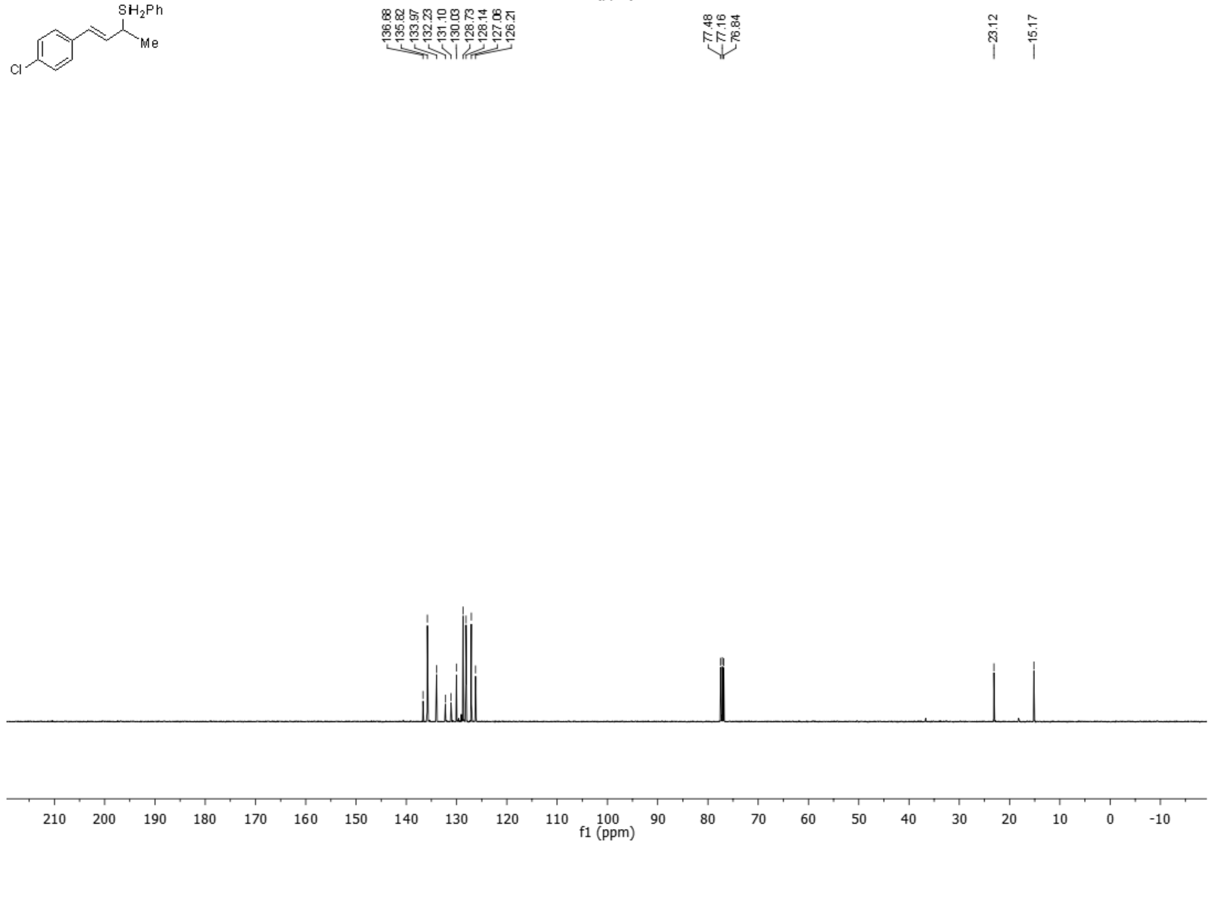

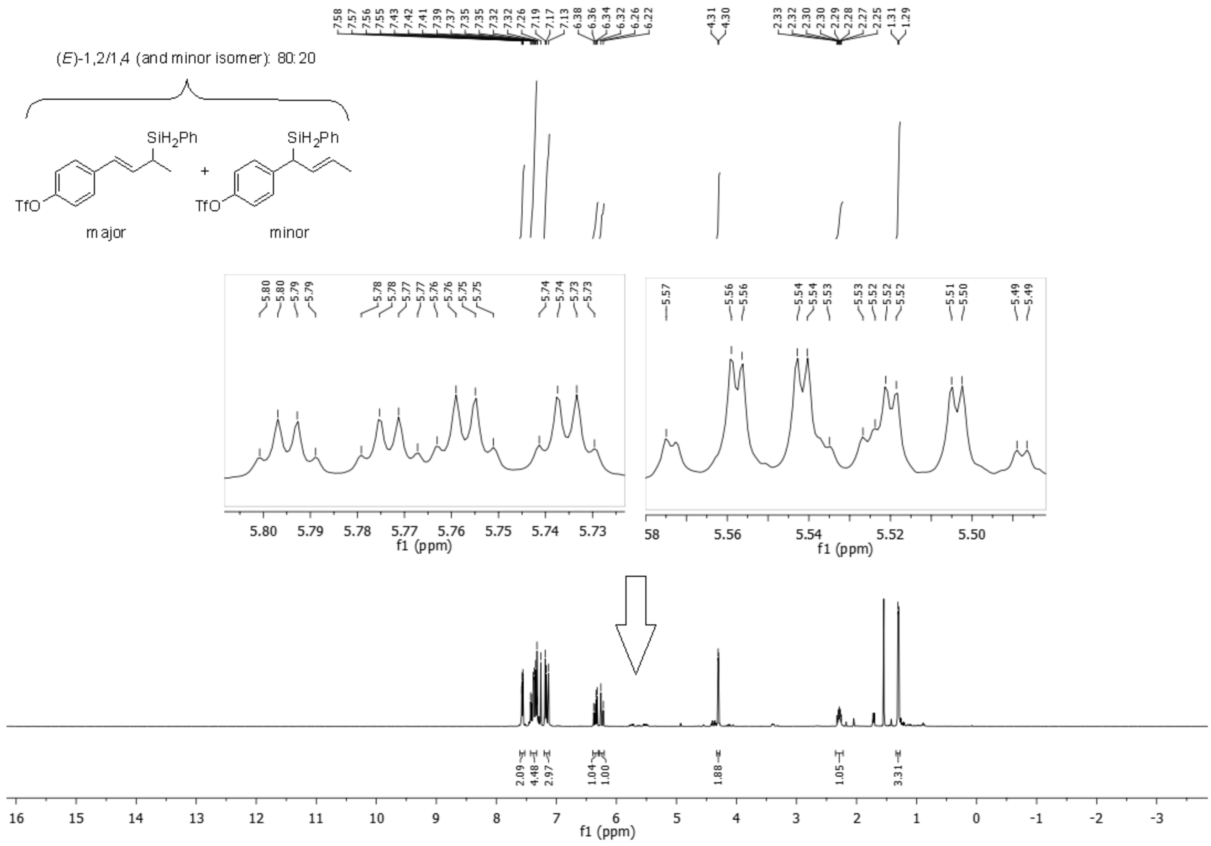
(E)-1,2/1,4 (and minor isomer): 80:20
SiH2Ph
TfO
major
minor
f1 (ppm)

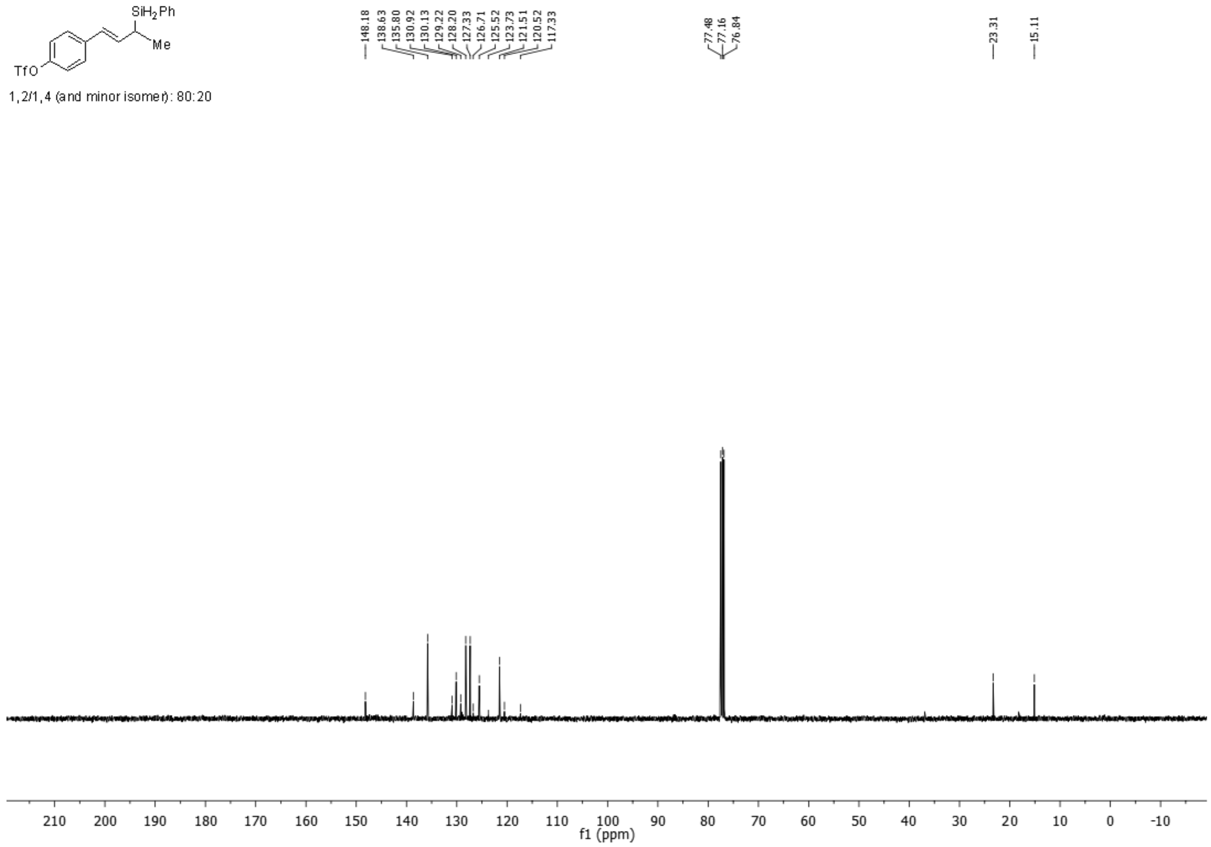
SiH2Ph
Me
TfO
1,2/1,4 (and minor isomer): 80:20
f1 (ppm)

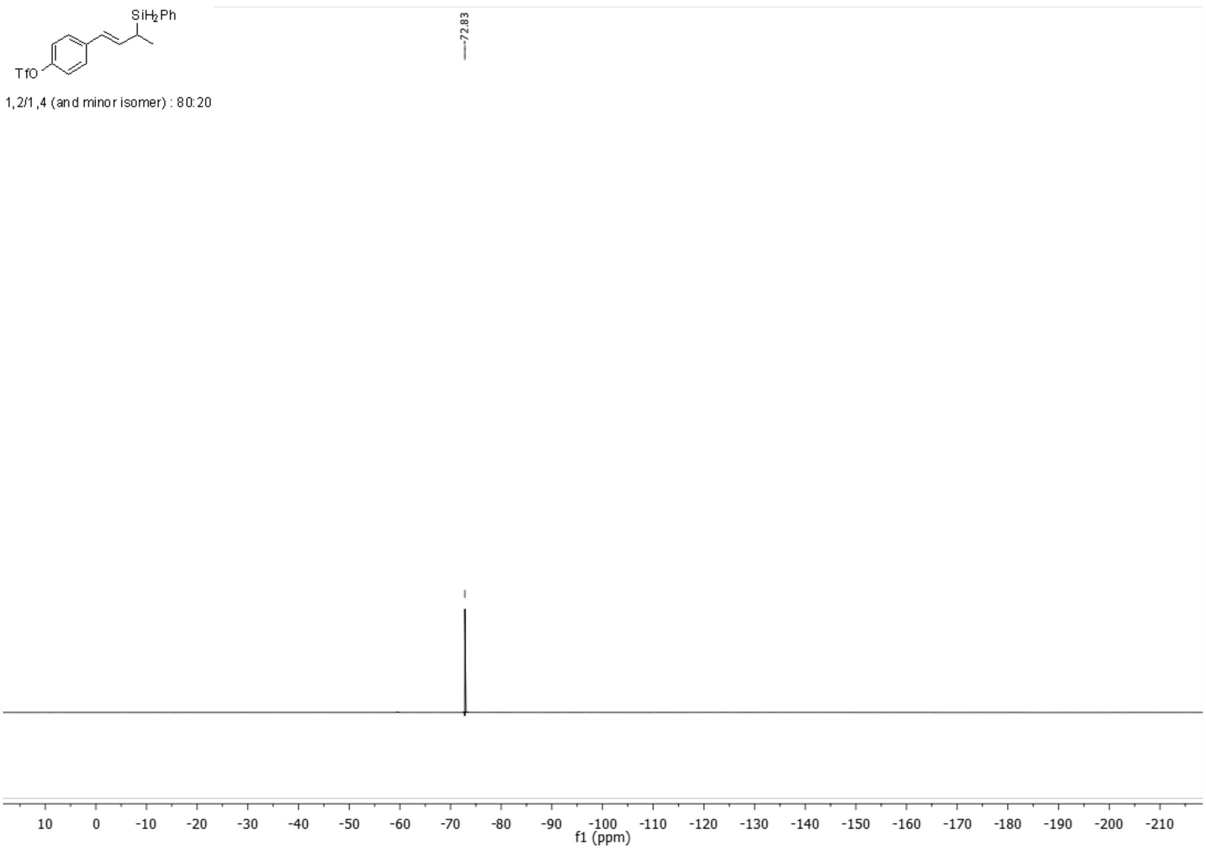
SiH2Ph
TfO
1,2/1,4 (and minor isomer) : 80:20
-72.83
10 0 -10 -20 -30 -40 -50 -60 -70 -80 -90 -100 -110 -120 -130 -140 -150 -160 -170 -180 -190 -200 -210
f1 (ppm)

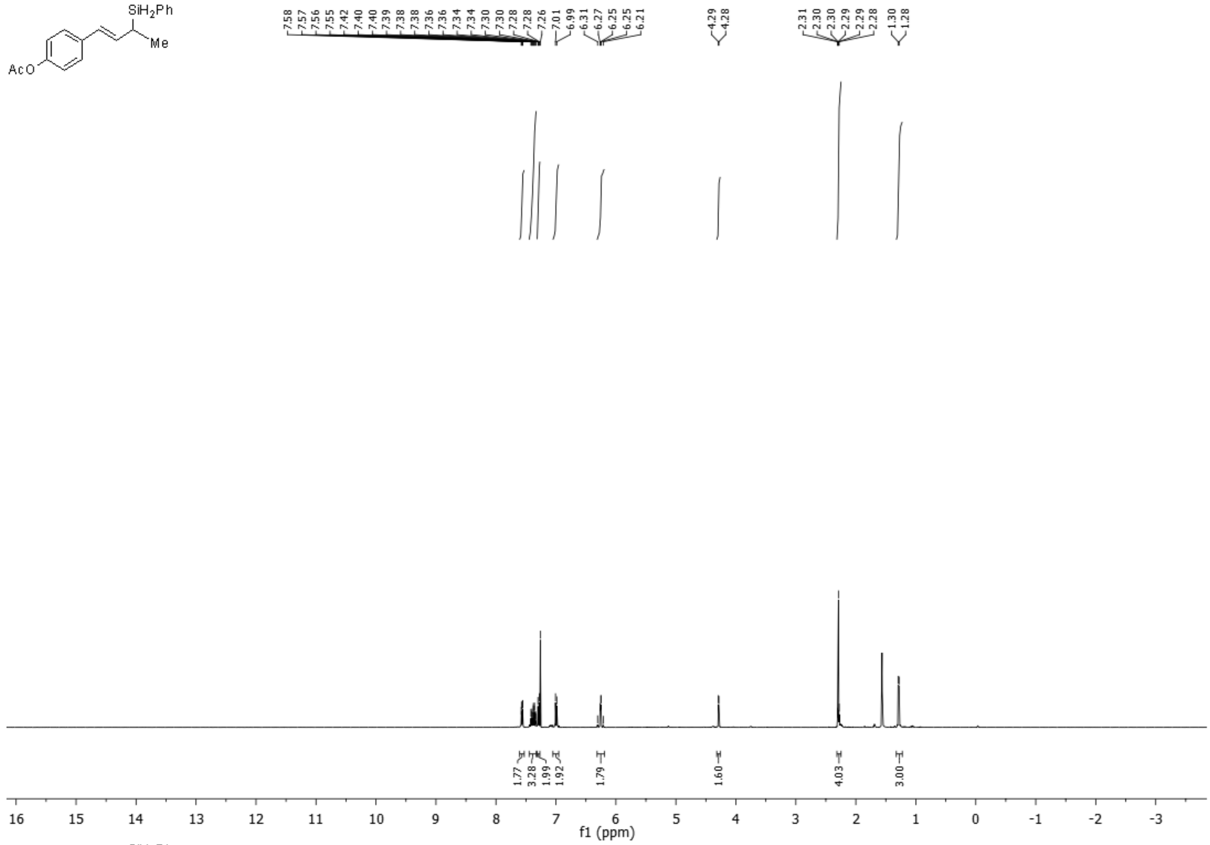
SiH2Ph
Me
AcO
7.58 7.57 7.56 7.55 7.42 7.40 7.39 7.38 7.38 7.36 7.36 7.34 7.34 7.30 7.30 7.28 7.28 7.26 7.01 6.99 6.31 6.27 6.25 6.25 6.21
4.29 4.28
2.31 2.30 2.30 2.29 2.29 2.28 1.30 1.28
1.77 3.28 1.99 1.92 1.79 1.60 4.03 3.00
f1 (ppm)

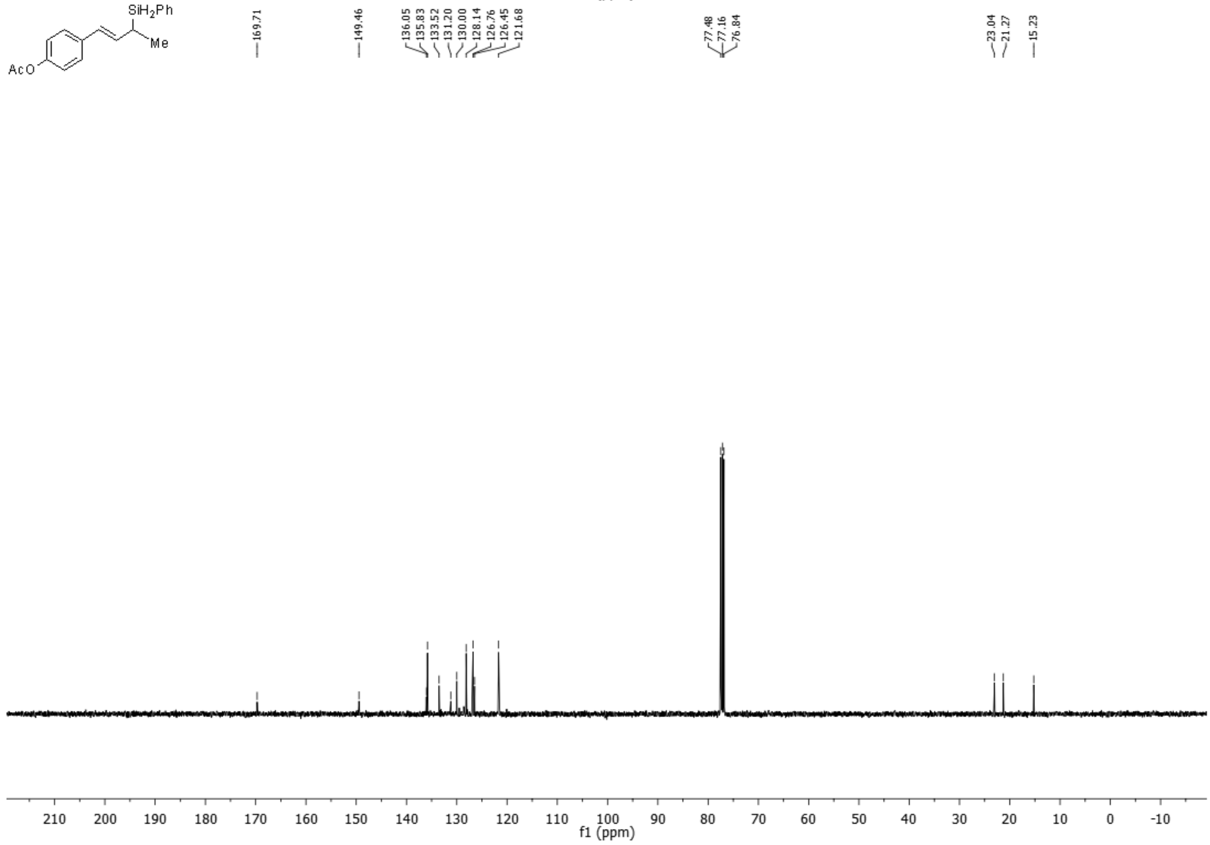
SiH2Ph
Me
AcO
169.71
149.46
136.05 135.83 133.52 131.20 130.00 128.14 126.76 126.45 121.68
77.48 77.16 76.84
23.04 21.27
15.23
f1 (ppm)

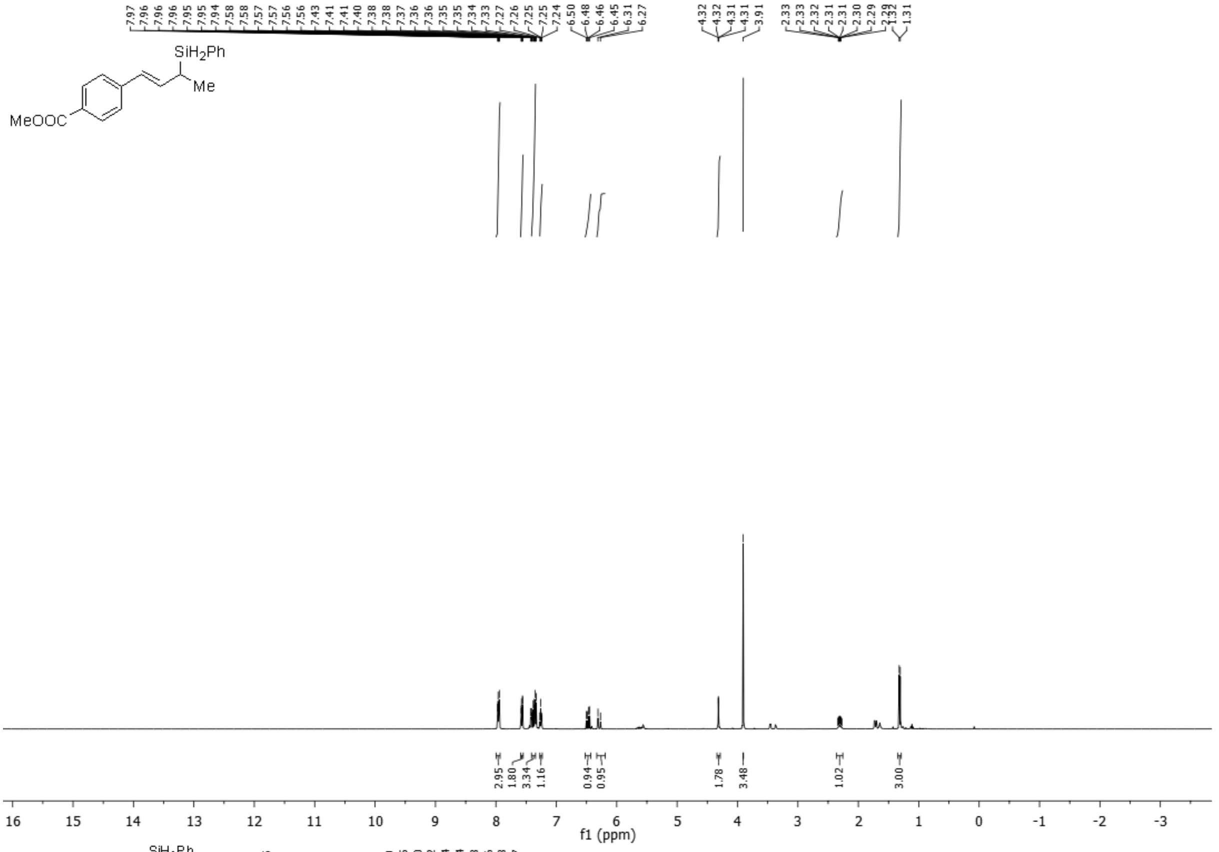
SiH2Ph
Me
MeOOC
7.97 7.96 7.96 7.95 7.95 7.94 7.58 7.58 7.57 7.57 7.56 7.43 7.41 7.40 7.38 7.38 7.37 7.36 7.36 7.35 7.35 7.34 7.33 7.27 7.26 7.25 7.25 7.24 6.50 6.48 6.46 6.45 6.31 6.27 4.32 4.32 4.31 4.31 3.91 2.33 2.33 2.32 2.31 2.31 2.30 2.29 2.29 1.32 1.31
2.95 1.80 3.34 1.16 0.94 0.95 1.78 3.48 1.02 3.00
f1 (ppm)

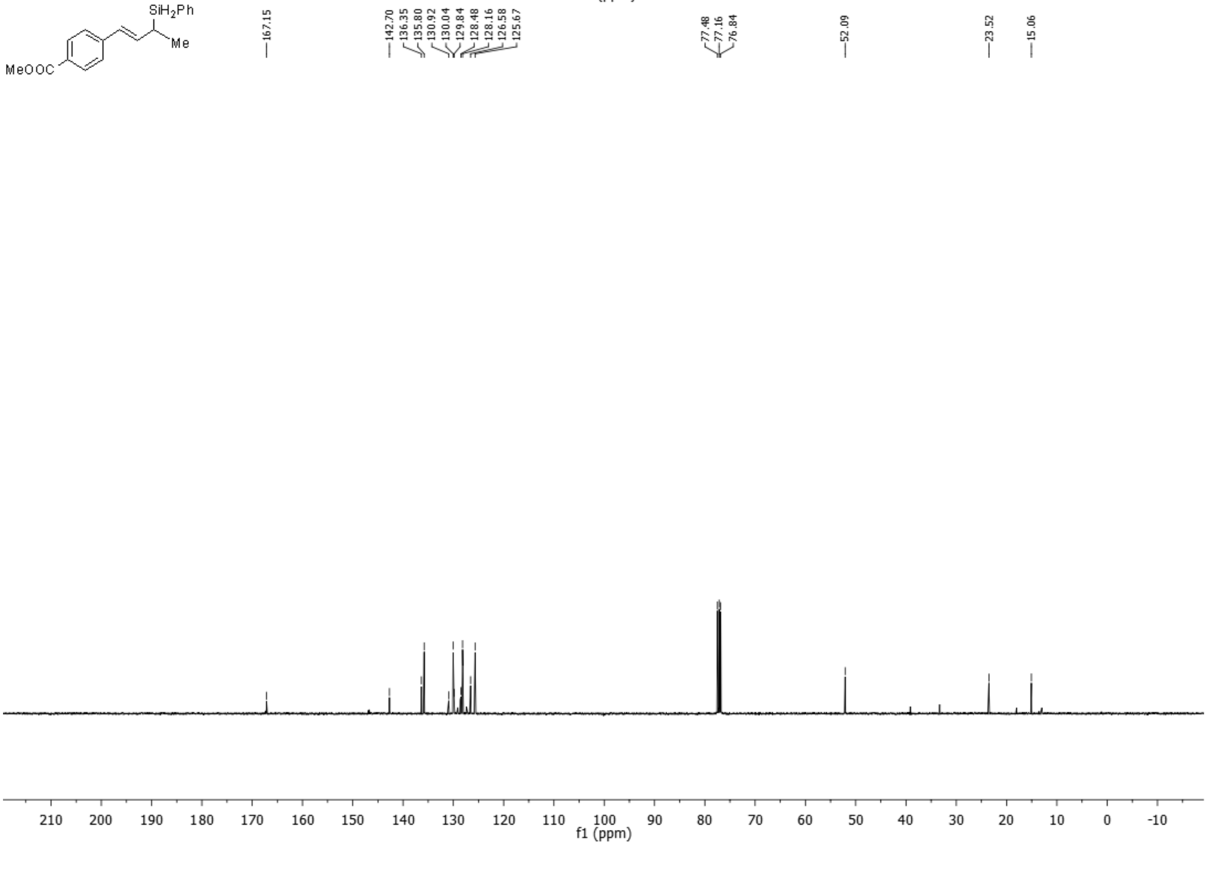
SiH2Ph
Me
MeOOC
167.15
142.70 136.35 135.80 130.92 130.04 129.84 128.48 128.16 126.58 125.67
77.48 77.16 76.84
52.09
23.52
15.06
f1 (ppm)

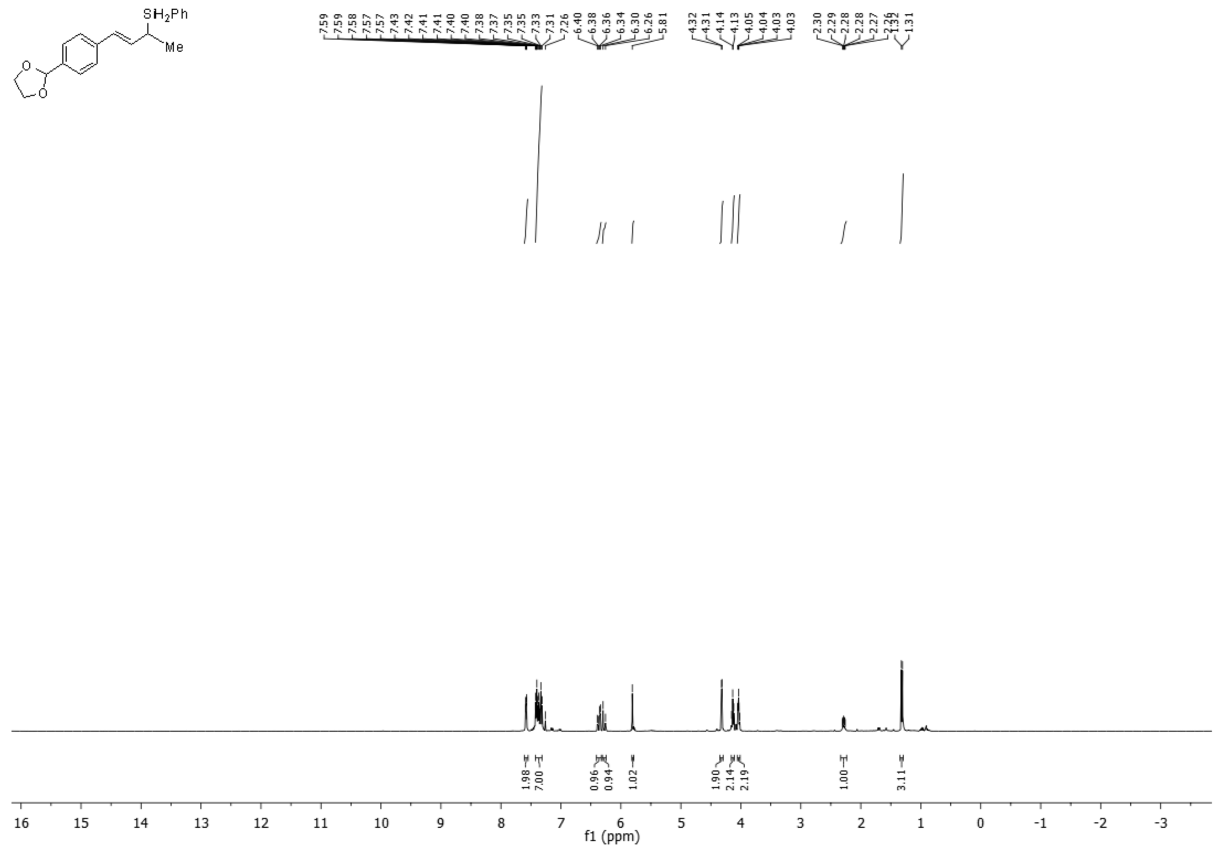

SPh
Me
O
O
7.59 7.59 7.58 7.57 7.57 7.43 7.42 7.41 7.41 7.40 7.40 7.38 7.37 7.35 7.35 7.33 7.31 7.26 6.40 6.38 6.36 6.34 6.30 6.26 5.81
4.32 4.31 4.14 4.13 4.05 4.04 4.03 4.03
2.30 2.29 2.28 2.28 2.27 2.26 1.32 1.31
1.98 7.00 0.96 0.94 1.02 1.90 2.14 2.19 1.00 3.11
f1 (ppm)


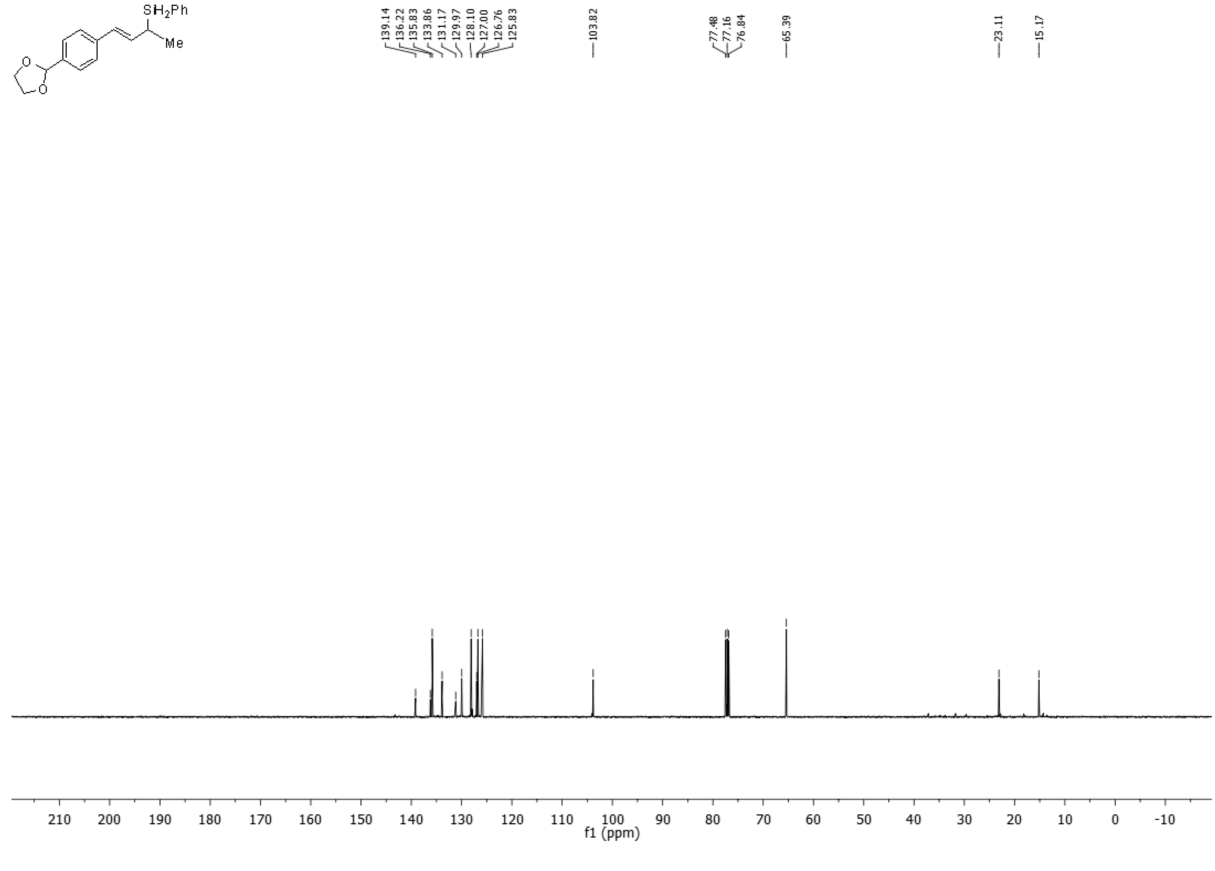

SPh
Me
O
O
139.14 136.22 135.83 133.86 131.17 129.97 128.10 127.00 126.76 125.83
103.82
77.48 77.16 76.84
65.39
23.11
15.17
f1 (ppm)

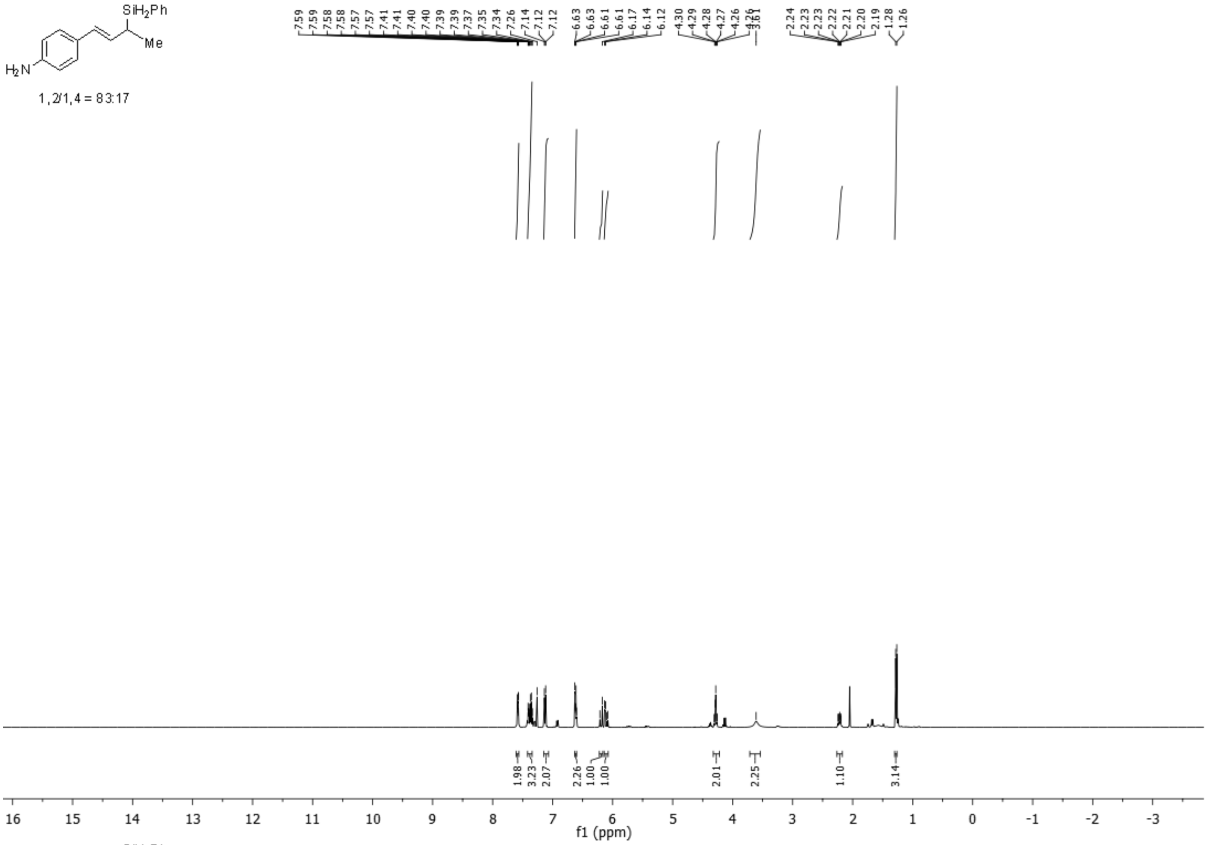


1,2/1,4 = 83:17

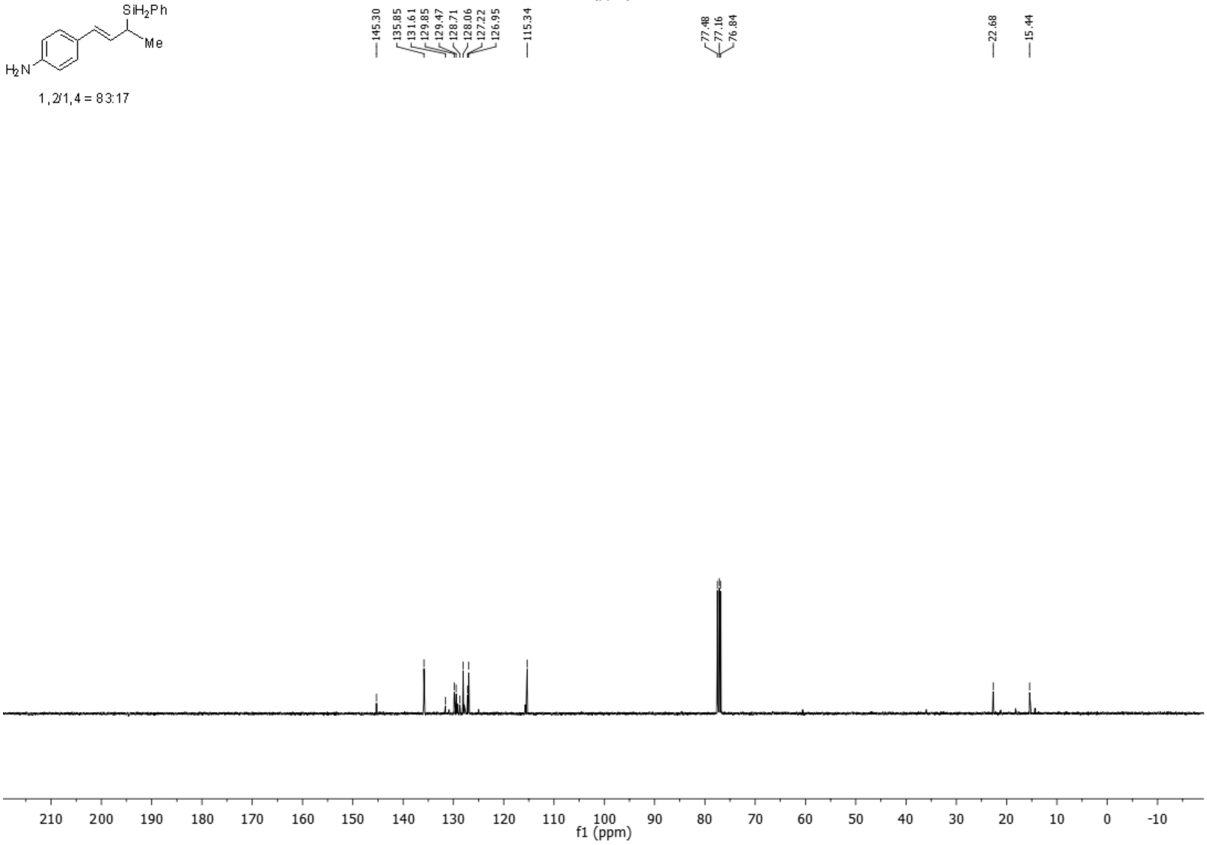


1,2/1,4 = 83:17

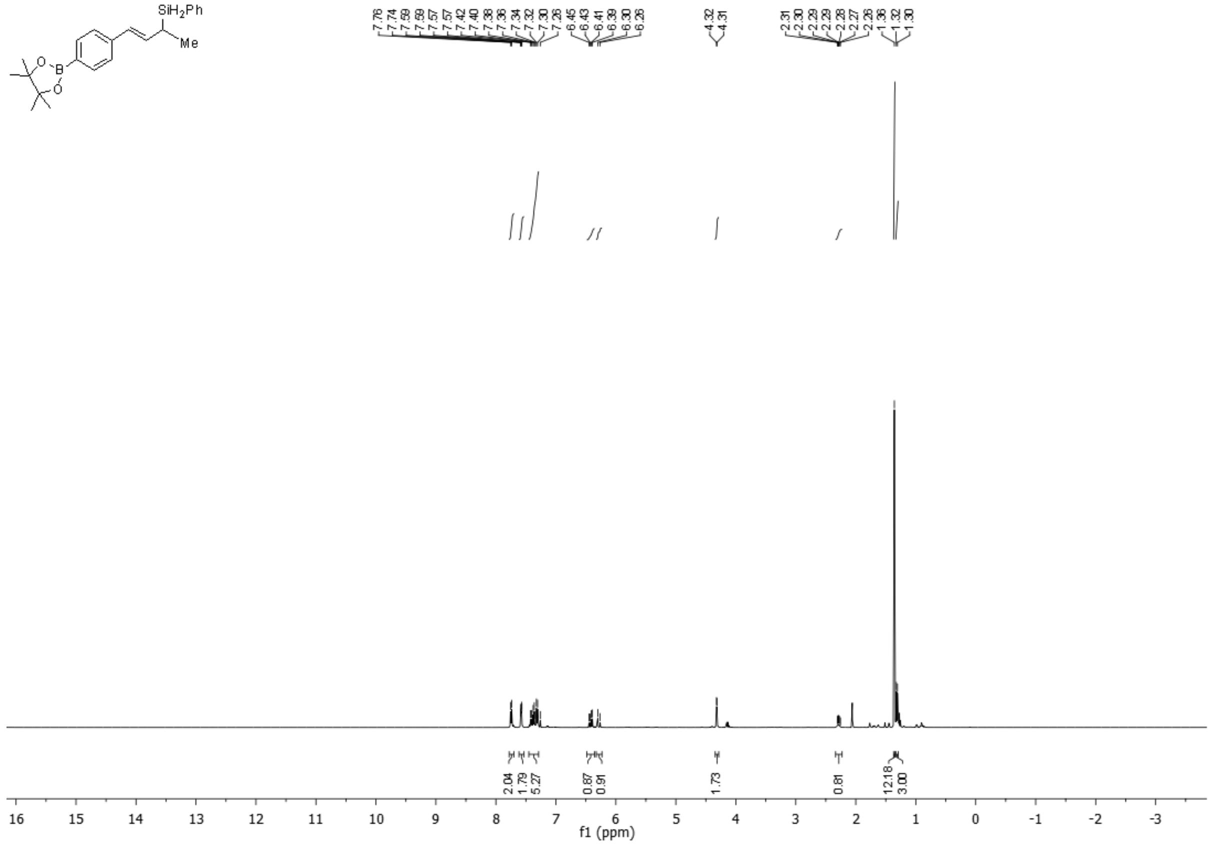

SiH2Ph
Me
B
O
O
2.04
1.79
5.27
0.87
0.91
1.73
0.81
12.18
3.00
f1 (ppm)


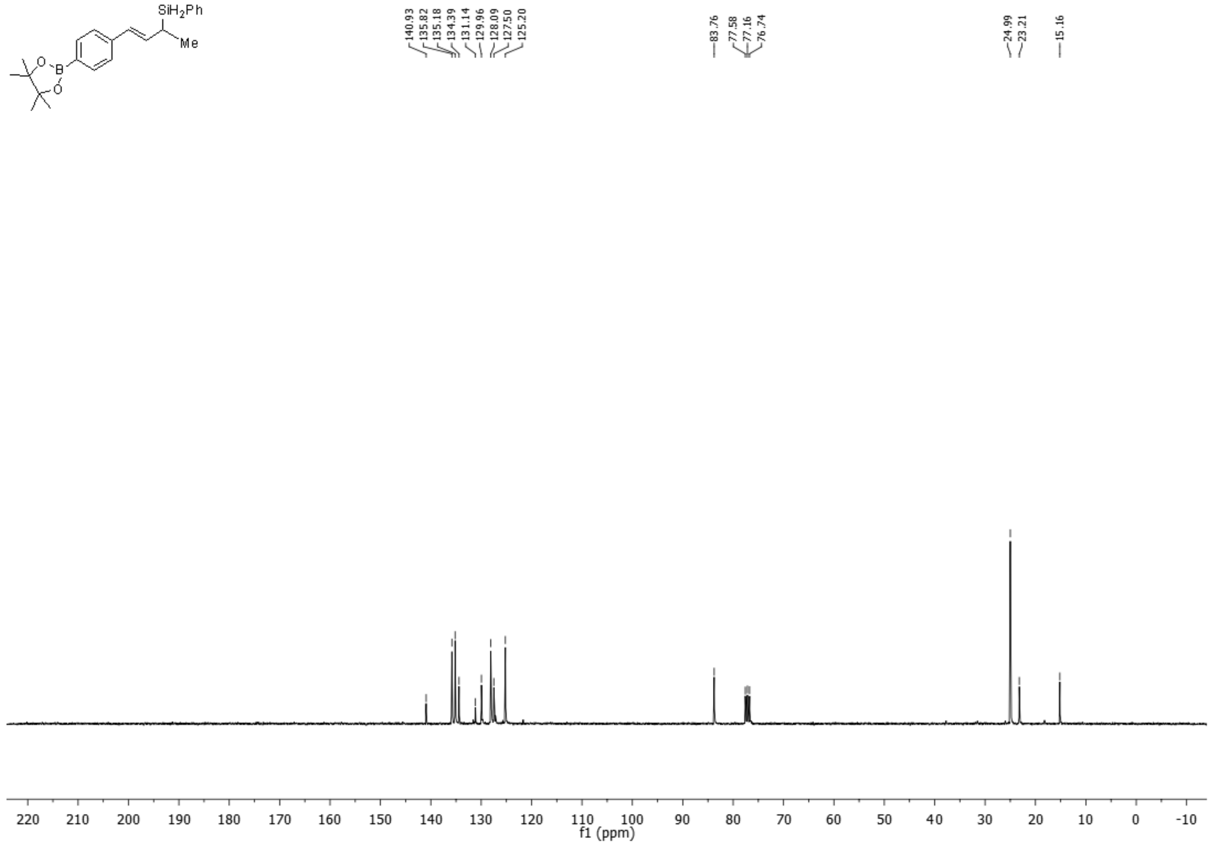

SiH2Ph
Me
B
O
O
140.93
135.82
135.18
134.39
131.14
129.96
128.09
127.50
125.20
83.76
77.58
77.16
76.74
24.99
23.21
15.16
f1 (ppm)

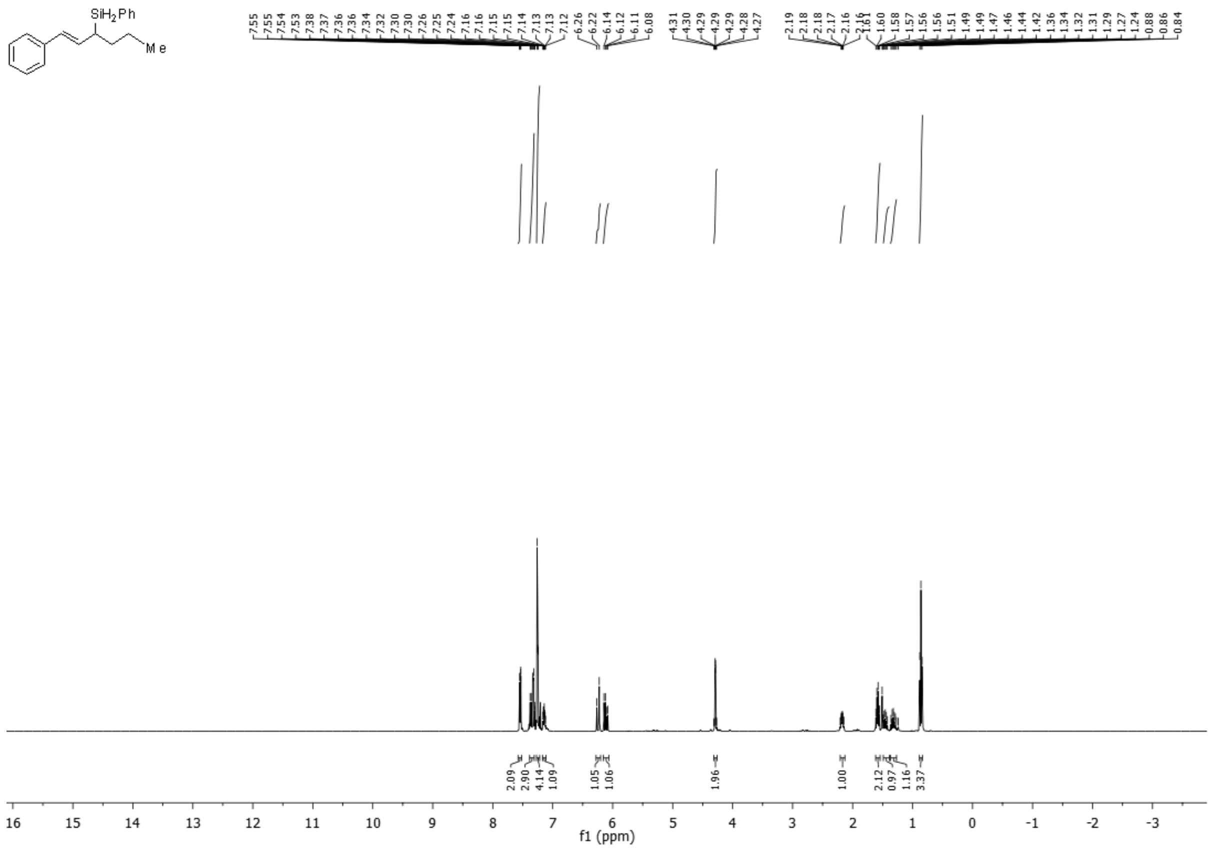
SiH2Ph
Me
f1 (ppm)

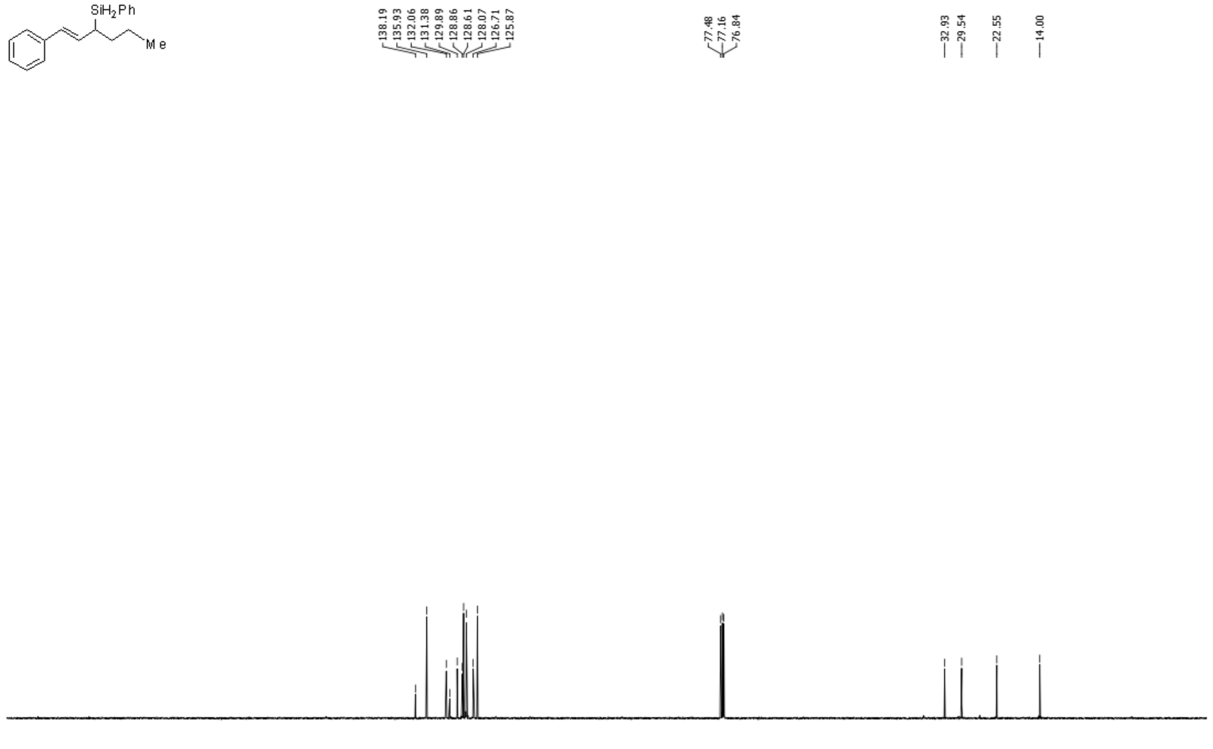
SiH2Ph
Me
f1 (ppm)

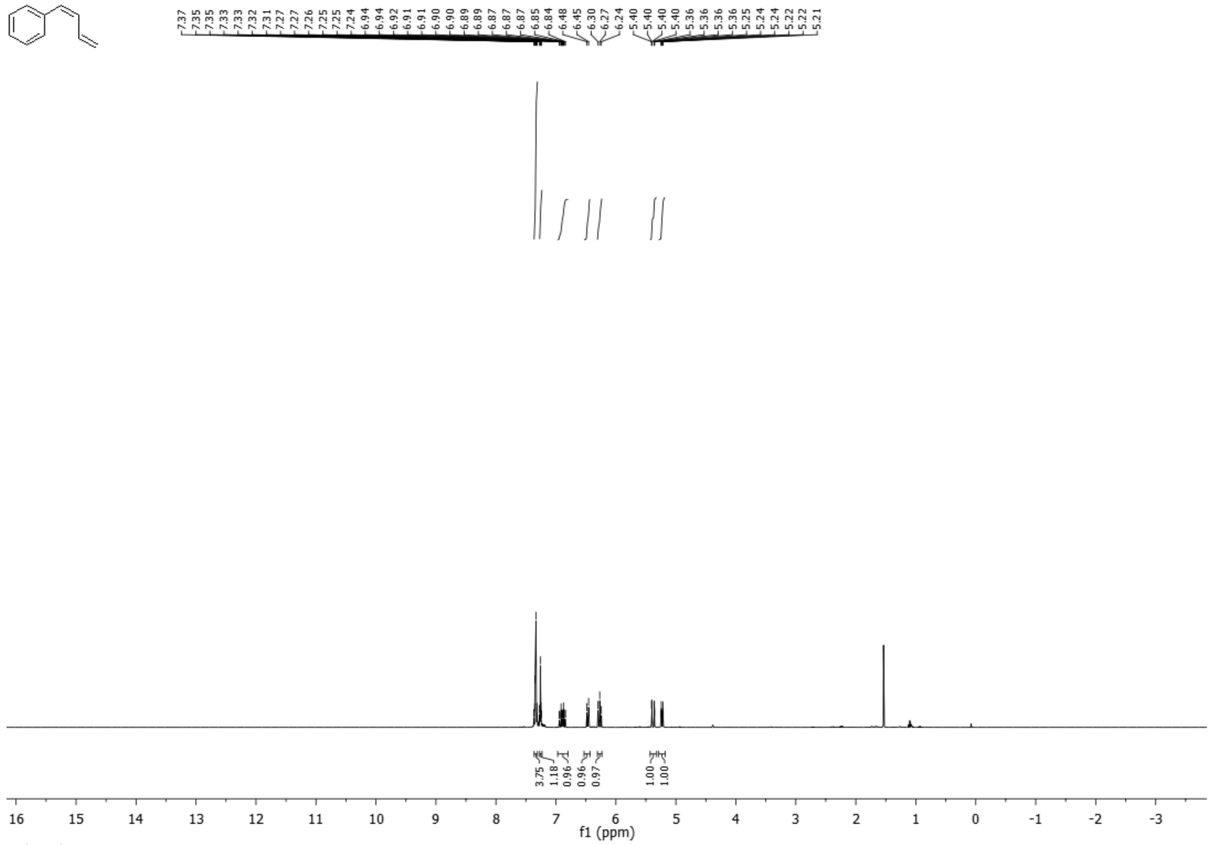
7.37 7.35 7.35 7.33 7.33 7.32 7.31 7.27 7.27 7.26 7.25 7.25 7.24 6.94 6.94 6.92 6.91 6.91 6.90 6.90 6.89 6.89 6.87 6.87 6.85 6.84 6.48 6.45 6.30 6.27 6.24 5.40 5.40 5.40 5.40 5.36 5.36 5.36 5.36 5.25 5.24 5.24 5.22 5.22 5.21
3.75 1.18 0.96 0.96 0.97 1.00 1.00
16 15 14 13 12 11 10 9 8 7 6 5 4 3 2 1 0 -1 -2 -3
f1 (ppm)

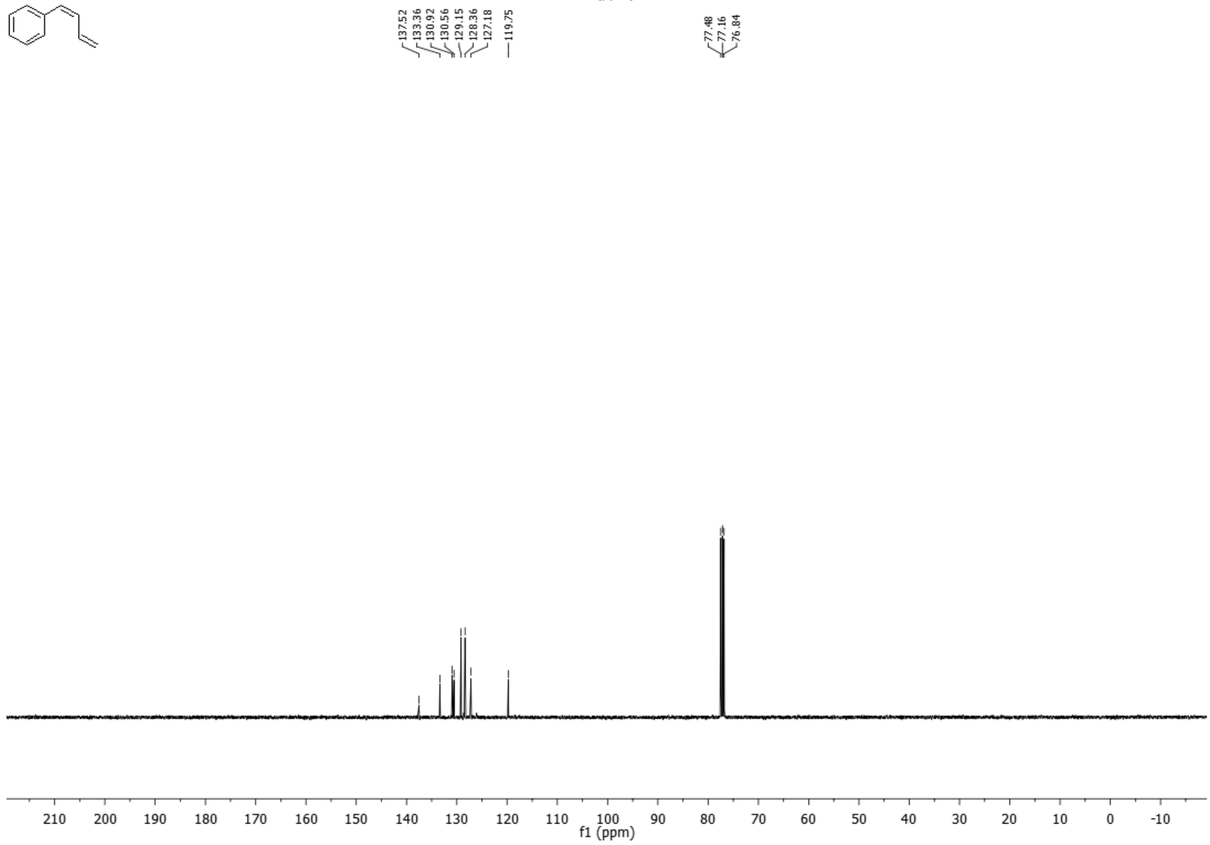
137.52 133.36 130.92 130.56 129.15 128.36 127.18 119.75
77.48 77.16 76.84
210 200 190 180 170 160 150 140 130 120 110 100 90 80 70 60 50 40 30 20 10 0 -10
f1 (ppm)

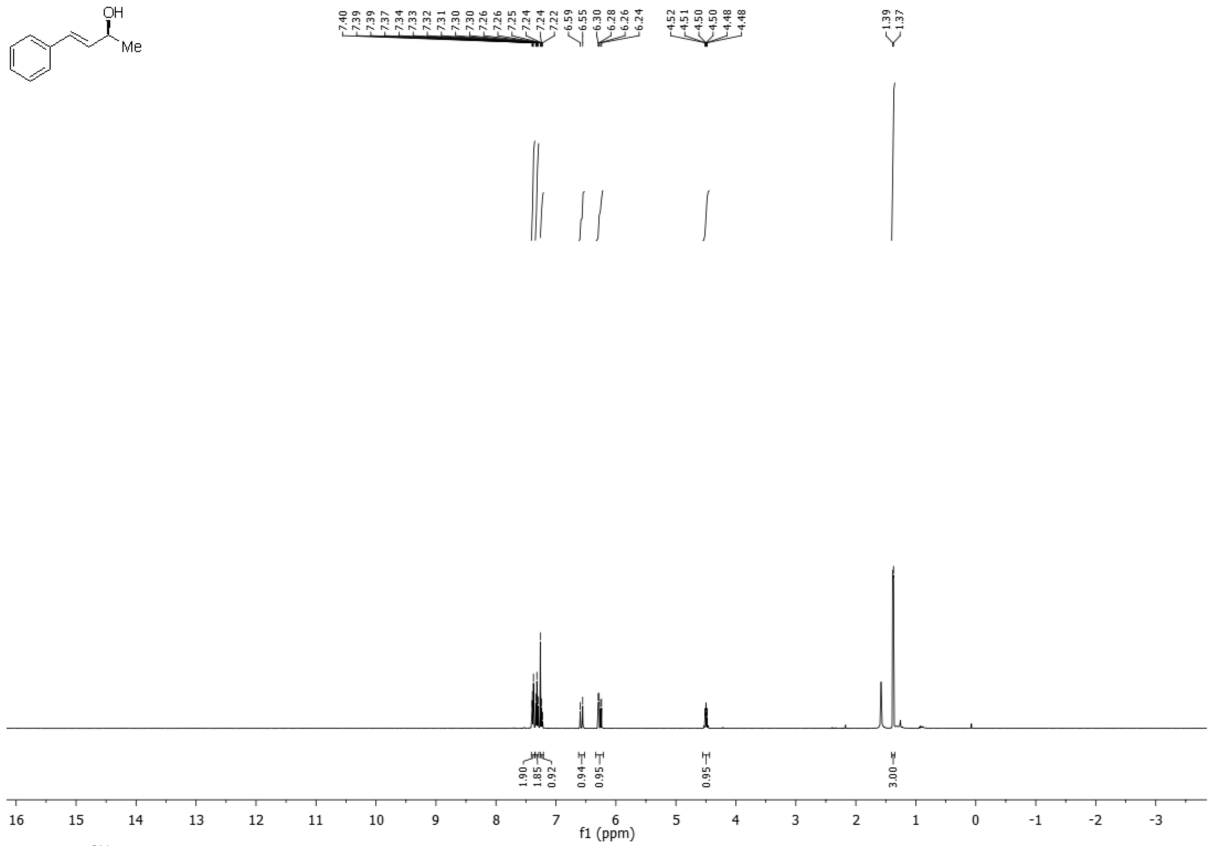
OH
Me
7.40
7.39
7.39
7.37
7.34
7.33
7.32
7.31
7.30
7.30
7.26
7.26
7.25
7.24
7.24
7.22
6.59
6.55
6.30
6.28
6.26
6.24
4.52
4.51
4.50
4.50
4.48
4.48
1.39
1.37
1.90
1.85
0.92
0.94
0.95
0.95
3.00
f1 (ppm)

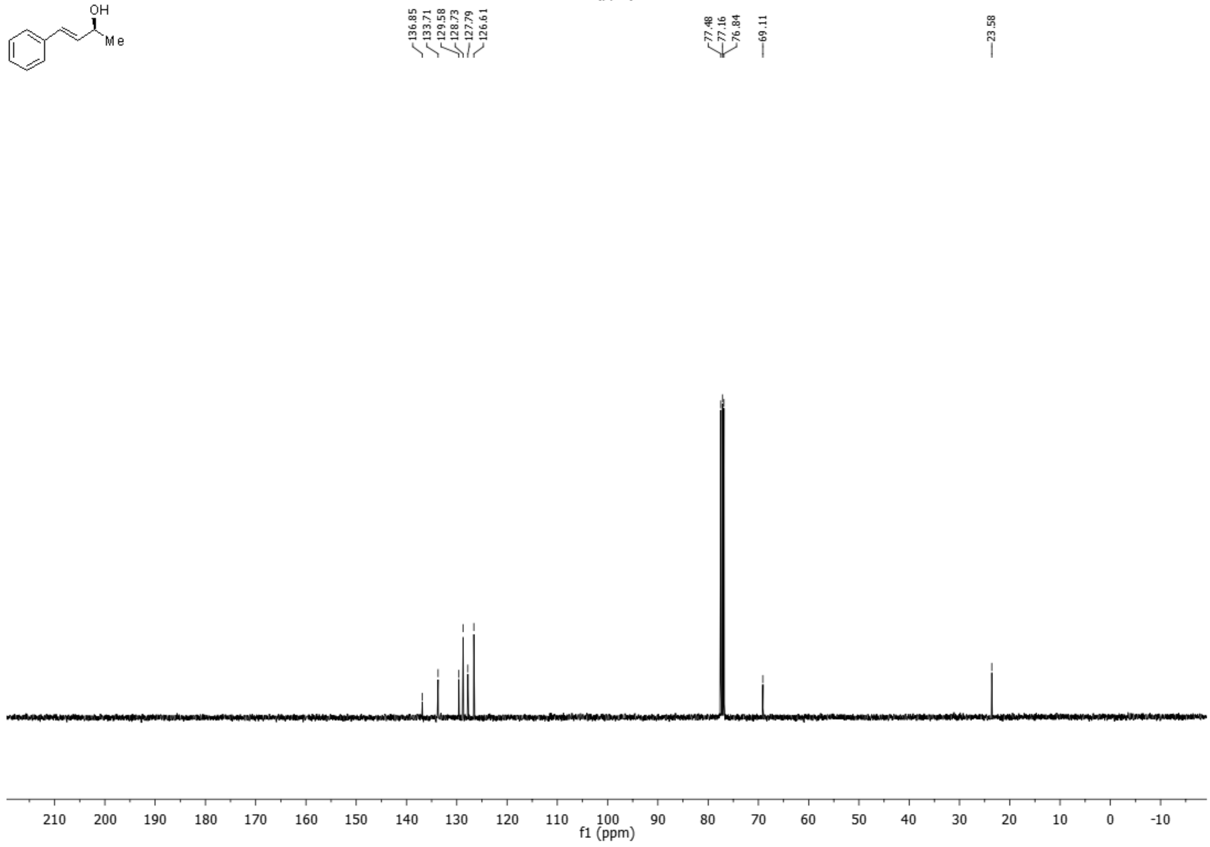
OH
Me
136.85
133.71
129.58
128.73
127.79
126.61
77.48
77.16
76.84
69.11
23.58
f1 (ppm)

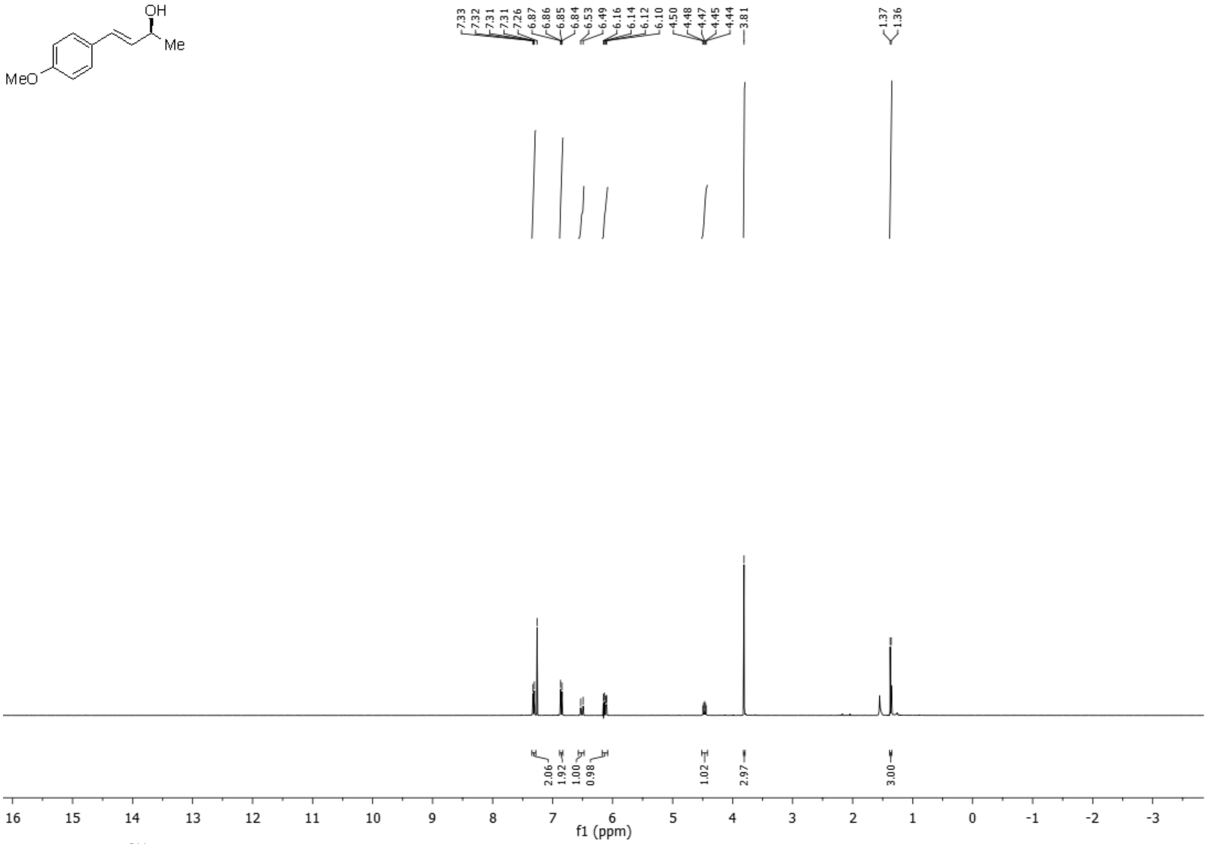
OH
Me
MeO
7.33
7.32
7.31
7.31
7.26
6.87
6.86
6.85
6.84
6.53
6.49
6.16
6.14
6.12
6.10
4.50
4.48
4.47
4.45
4.44
3.81
1.37
1.36
2.06
1.92
1.00
0.98
1.02
2.97
3.00
f1 (ppm)

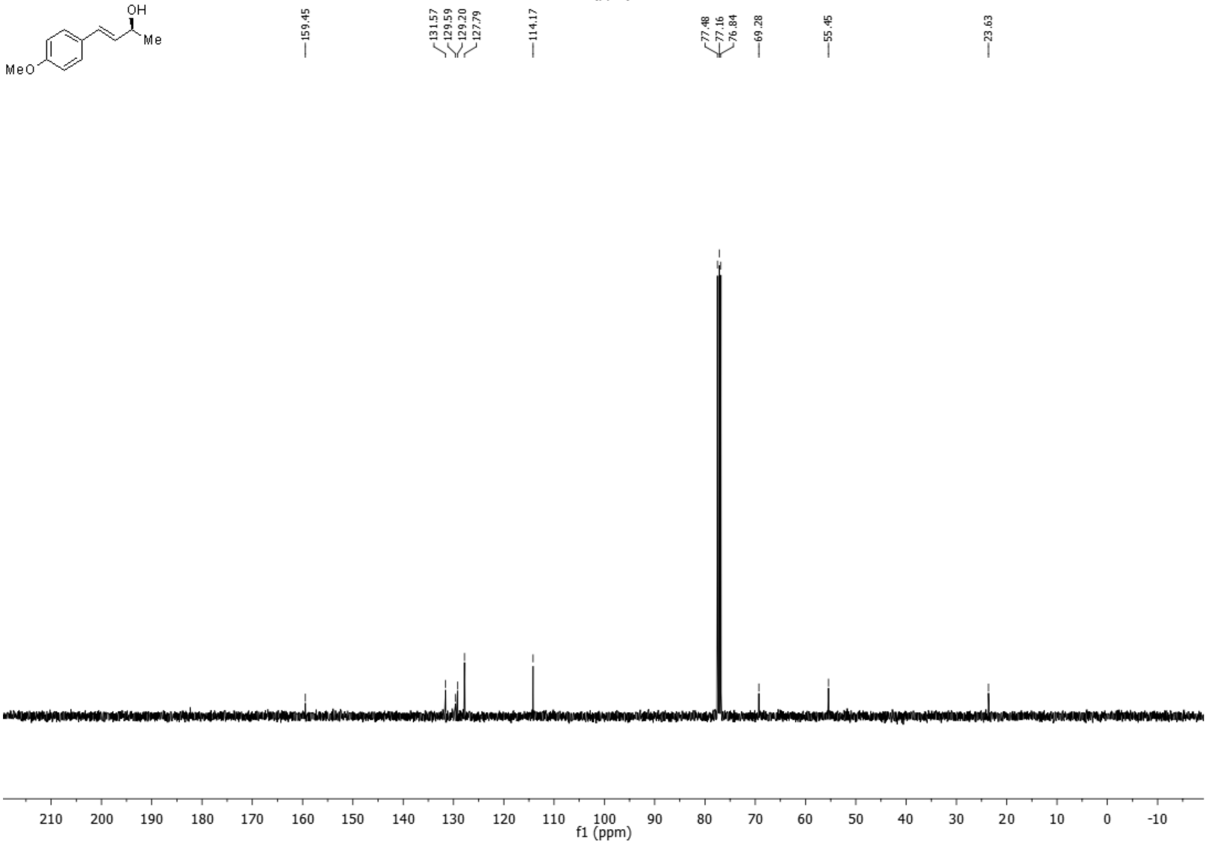
OH
Me
MeO
159.45
131.57
129.59
129.20
127.79
114.17
77.48
77.16
76.84
69.28
55.45
23.63
f1 (ppm)
